# A scoping review of the proximal humerus fracture literature

**DOI:** 10.1186/s12891-015-0564-8

**Published:** 2015-05-10

**Authors:** Gerard P Slobogean, Herman Johal, Kelly A Lefaivre, Norma J MacIntyre, Sheila Sprague, Taryn Scott, Pierre Guy, Peter A Cripton, Michael McKee, Mohit Bhandari

**Affiliations:** Division of Orthopaedic Surgery, Department of Surgery, Master University, 293 Wellington Street North, Suite 110, Hamilton, ON Canada; Department of Orthopaedics, University of British Columbia, 3114-910 West 10th Avenue, Vancouver, BC Canada; Division of Orthopaedic Trauma, Department of Orthopaedics, R Adams Cowley Shock Trauma Center, University of Maryland, 22 S. Greene Street, Baltimore, MD USA; School of Rehabilitation Science, McMaster University, 1400 Main Street West, Hamilton, ON Canada; Department of Clinical Epidemiology and Biostatistics, McMaster University, 293 Wellington Street North, Suite 110, Hamilton, ON Canada; Department of Mechanical Engineering, University of British Columbia, 2054-6250 Applied Science Lane, Vancouver, BC Canada; Division of Orthopaedic Surgery, Department of Surgery, University of Toronto, 149 College Street, Toronto, ON Canada

**Keywords:** Proximal humerus fracture, Scoping review, Orthopaedic trauma, Fragility fracture

## Abstract

**Background:**

Proximal humerus fractures are a common fragility fracture that significantly affects the independence of older adults. The outcomes of these fractures are frequently disappointing and previous systematic reviews are unable to guide clinical practice. Through an integrated knowledge user collaboration, we sought to map the breadth of literature available to guide the management of proximal humerus fractures.

**Methods:**

We utilized a scoping review technique because of its novel ability to map research activity and identify knowledge gaps in fields with diverse treatments. Through multiple electronic database searches, we identified a comprehensive body of proximal humerus fracture literature that was classified into eight research themes. Meta-data from each study were abstracted and descriptive statistics were used to summarize the results.

**Results:**

1,051 studies met our inclusion criteria with the majority of research being performed in Europe (64%). The included literature consists primarily of surgical treatment studies (67%) and biomechanical fracture models (10%). Nearly half of all clinical studies are uncontrolled case series of a single treatment (48%). Non-randomized comparative studies represented 12% of the literature and only 3% of the studies were randomized controlled trials. Finally, studies with a primary outcome examining the effectiveness of non-operative treatment or using a prognostic study design were also uncommon (4% and 6%, respectively).

**Conclusions:**

The current study provides a comprehensive summary of the existing proximal humerus fracture literature using a thematic framework developed by a multi-disciplinary collaboration. Several knowledge gaps have been identified and have generated a roadmap for future research priorities.

**Electronic supplementary material:**

The online version of this article (doi:10.1186/s12891-015-0564-8) contains supplementary material, which is available to authorized users.

## Background

Fragility fractures are a common injury with a significant economic impact on all health care systems. It is estimated that at least 1 in 3 women and 1 in 5 men will suffer a fragility fracture in their lifetime [1], and the cost of treating osteoporotic fractures in Canada is more than $2.3 billion dollars per year (2010 Canadian dollars) [2]. Proximal humerus fractures comprise a significant proportion of all fragility fractures and typically occur in elderly adults as a result of minimal trauma [3,4]. Recent estimates suggest these injuries are responsible for 185,000 visits to emergency departments in the United States per year [5]. In a retrospective study conducted at three Ontario community hospitals, proximal humerus fractures accounted for 20% of all fragility fractures seen in the outpatient fracture clinics [4]. In a larger population based study of 1,027 proximal humerus fractures, the vast majority of injuries occurred in active adults older than 60 years, with the greatest incidence occurring in women ages 80 to 89 years [3]. These authors also noted that more than 90% of the patients with proximal humerus fractures lived at home and over 80% performed their own shopping and housework. As a result, these fractures have the potential to significantly affect the independence and quality of life of older adults.

The acute treatment options for proximal humerus fractures are numerous and are typically guided by the fracture pattern and patient’s functional demands. The most commonly used methods include non-operative management with a sling, surgical fixation, or shoulder arthroplasty. Following the initial treatment, the post-injury rehabilitation is also subject to numerous variations in the physiotherapy protocol [6]. Unfortunately, the increasing number of treatment options combined with a lack of comparative clinical studies has made it difficult for clinicians to select the optimum management of proximal humerus fractures. Furthermore, regardless of the treatment selected many clinical studies report disappointing functional outcomes including residual shoulder pain, limitations in shoulder motion, and decreased quality of life.

Recognizing the controversy in treatment and the poor functional outcomes of proximal humerus fractures, we organized a group of community members and clinicians interested in improving the care of these injuries. The purpose of this study was to determine “What literature is available to guide the acute management of proximal humerus fractures?” We hypothesized that the existing literature could be mapped into distinct themes to identify strengths and limitations in each area of research, and that these results could then be used to develop future research priorities.

## Methods

### Overview

Most syntheses of existing medical literature use meta-analysis techniques to quantitatively pool published data. Several authors have attempted to quantitatively synthesize various research questions within the proximal humerus fracture literature; however, the lack of comparative trials and substantial study heterogeneity has led to multiple reviews that are unable to provide useful clinical recommendations [7,8].

The lack of clinical trials has repeatedly been the limiting factor for proximal humerus fracture systematic reviews and meta-analyses because relevant randomized controlled trials represent less than 1% of the proximal humerus fracture literature indexed in MEDLINE. As a result, the overwhelming majority of literature on this injury has not been summarized. Given the recurrent challenges of conducting meta-analyses involving the treatment of proximal humerus fractures [7] we recognized that novel knowledge synthesis techniques would be required in order to utilize a larger portion of relevant literature.

A scoping review is an increasingly popular literature review method that allows researchers to summarize a range of evidence in order to describe the breadth and depth of a field [9]. Unlike systematic reviews a scoping review typically addresses broader research questions where many different interventions or study designs might be relevant [10]. The treatment literature for proximal humerus fractures is rapidly expanding with emerging techniques and new implants. With diverse proximal humerus fracture treatment options and a substantial lack of clinical trials, performing a scoping review to map the extent, range, and nature of available research was the most appropriate synthesis methodology.

No human subjects were involved in this research; therefore, neither research ethics committee approval nor informed consent was required.

### Knowledge user collaboration

**Figure 1:**
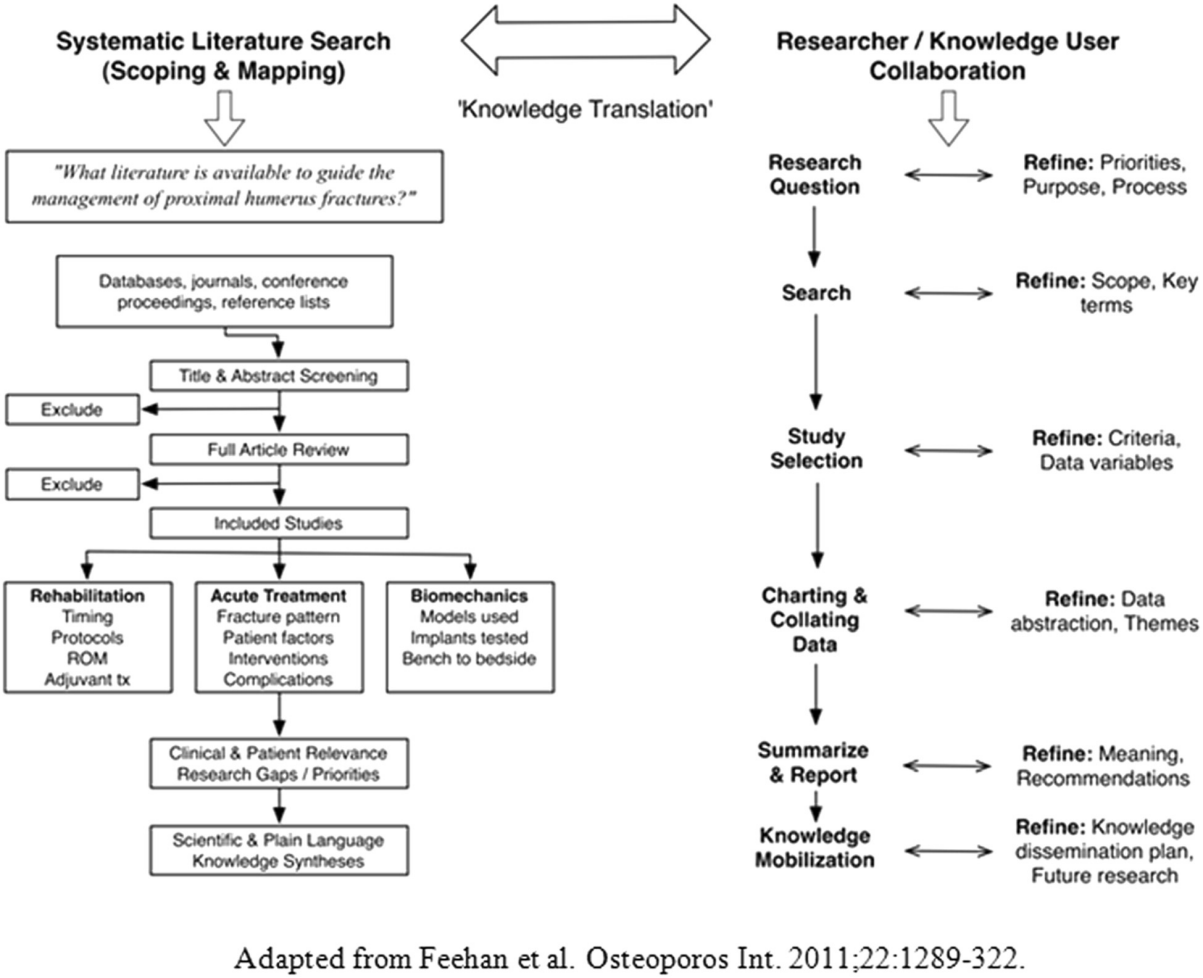
Scoping review overview.

Our research goals and methodology were directed by a collaboration of orthopaedic surgeons, physiotherapists, engineers, and patient advocacy group representatives interested in improving the care of proximal humerus fracture patients. This group of diverse participants is collectively described as the project’s Knowledge Users because they are “individuals likely able to use the knowledge generated through [this] research to make informed decisions about health policies, programs, and/or practices” [11]. Using the scoping review framework proposed by Arksey and O’Malley [10], we adopted an integrated research process that ensured the knowledge users input throughout all six stages of the review’s methodology (Figure 1). Understanding the current state of the proximal humerus fracture literature, in particular, areas for potential evidence-based recommendations and future research priorities, was defined as the primary purpose of the review.

### Literature search

In consultation with a biomedical librarian, we developed a sensitive search strategy to identify all types of publications involving proximal humerus fractures. Several search strategies and sources were used to identify relevant studies. Using a combination of keywords and medical subject heading (MeSH) terms related to proximal humerus fractures, we searched the following electronic databases: MEDLINE, Embase, Cumulative Index of Nursing and Allied Health Literature (CINAHL), Cochrane Database of Systematic Reviews (CDSR), Proquest, Web of Science, Society of Automotive Engineers (SAE) digital library, and Transportation Research Board’s Transport Research International Documentation (TRID) database. All searches were performed in October 2012, and no language or date restrictions were employed. The details of the search strategy are outlined in Table 1.

**Table 1:** **Literature search strategy**

**Database**	**Search strategy**
***MEDLINE*** Oct 30, 2012	1. exp *humeral fractures/ (4514)
	2. exp *shoulder fractures/ (1816)
	3. (humer: adj15 fracture*).mp. (8582)
	4. or/1-3 (9143)
	5. proximal.mp. (153405)
	6. 4 and 5 (1918)
***EMBASE*** Oct 30, 2012	1. exp *humeral fractures/ (5255)
	2. exp *shoulder fractures/ (262)
	3. (humer: adj15 fracture*).mp. (10822)
	4. or/1-3 (10937)
	5. proximal.mp. (182778)
	6. 4 and 5 (2417)
***CINAHL*** Oct 30, 2012	1. MM “Humeral Fractures”
	2. proximal humerus fracture*
	3. proximal humeral fracture*
	4. or/1-3 = 568 citations
***CDSR & CENTRAL*** Oct 30, 2012	1. (humer: adj15 fracture*).mp (180)
	2. proximal.mp (3977)
	3. 1 and 2 (63)
***WEB OF SCIENCE*** Oct 30, 2012	“humeral fracture*” OR “humerus fracture*” OR “shoulder fracture*” and AND’ing it with proximal 402 citations
***PROQUEST DISSERTATIONS FILE*** Nov 1, 2012	“proximal humerus fracture” or “proximal humeral fracture” 20 dissertations
***SAE Digital Library*** November, 2012	humer*
***TRID*** November, 2012	trid.trb.org humer*

### Study selection

Titles from all database searches were compiled into a literature review program and an independent review process was performed for all identified studies. Each potentially eligible study was reviewed in duplicate by 2 of 3 Orthopaedic surgeons (GPS, HJ, KAL) with experience in data abstraction and literature syntheses. Study eligibility criteria were outlined in response to our knowledge users’ needs and after a preliminary review of the available literature. Briefly, studies were included if they involved the acute treatment of proximal humerus fractures, utilized a clinically-relevant fracture model, or involved a research question directly relevant to the management of these injuries. Examples of the latter included biomechanical studies that tested surgical implants or radiographic studies describing the classification of these injuries. Studies were excluded if they involved pediatric fractures, pathologic fractures, or the sequelae of non-acute fractures (such as fracture malunion, non-union, or humeral head osteonecrosis). In addition, studies focusing on the medical management of osteoporosis or case reports with fewer than 10 research participants were also excluded. Finally, review articles that were general to shoulder trauma or to all aspects of proximal humerus fractures were excluded; whereas, review articles on a specific treatment or fracture pattern were included.

### Literature themes

Prior to commencing the scoping review, we proposed a thematic framework that would be used to broadly map the research areas of the included studies. These themes were selected based on their relevance to the diverse knowledge needs of our collaborators. These themes were further refined and finalized during the iterative review process that included monthly teleconferences with our knowledge users. Ultimately, eight themes were selected: 1) operative treatment, 2) non-operative treatment, 3) biomechanics & basic science, 4) rehabilitation, 5) prognostic & epidemiology, 6) radiology & fracture classification, 7) anatomy, and 8) miscellaneous. Each included study was assigned a single theme based on its primary research question, recognizing that some studies had relevant secondary themes that would not be captured.

### Data abstraction

In addition to being assigned a primary research theme, important characteristics from each included study were abstracted to understand the characteristics of the literature. These study variables included the year of publication, geographic region of the study centre, language of publication, study design, study perspective, and sample size. All data were obtained from the study’s abstract or full-text publication. Non-English publications were partially translated as necessary to complete the data abstraction.

### Statistical analysis

Descriptive statistics were used to summarize all data. For continuous data, the mean and standard deviation or median and interquartile range (IQR) were reported based on the data’s distribution. Counts and proportions were used to describe all other data. No inferential statistical testing was performed.

## Results

### Citation retrieval

**Figure 2:**
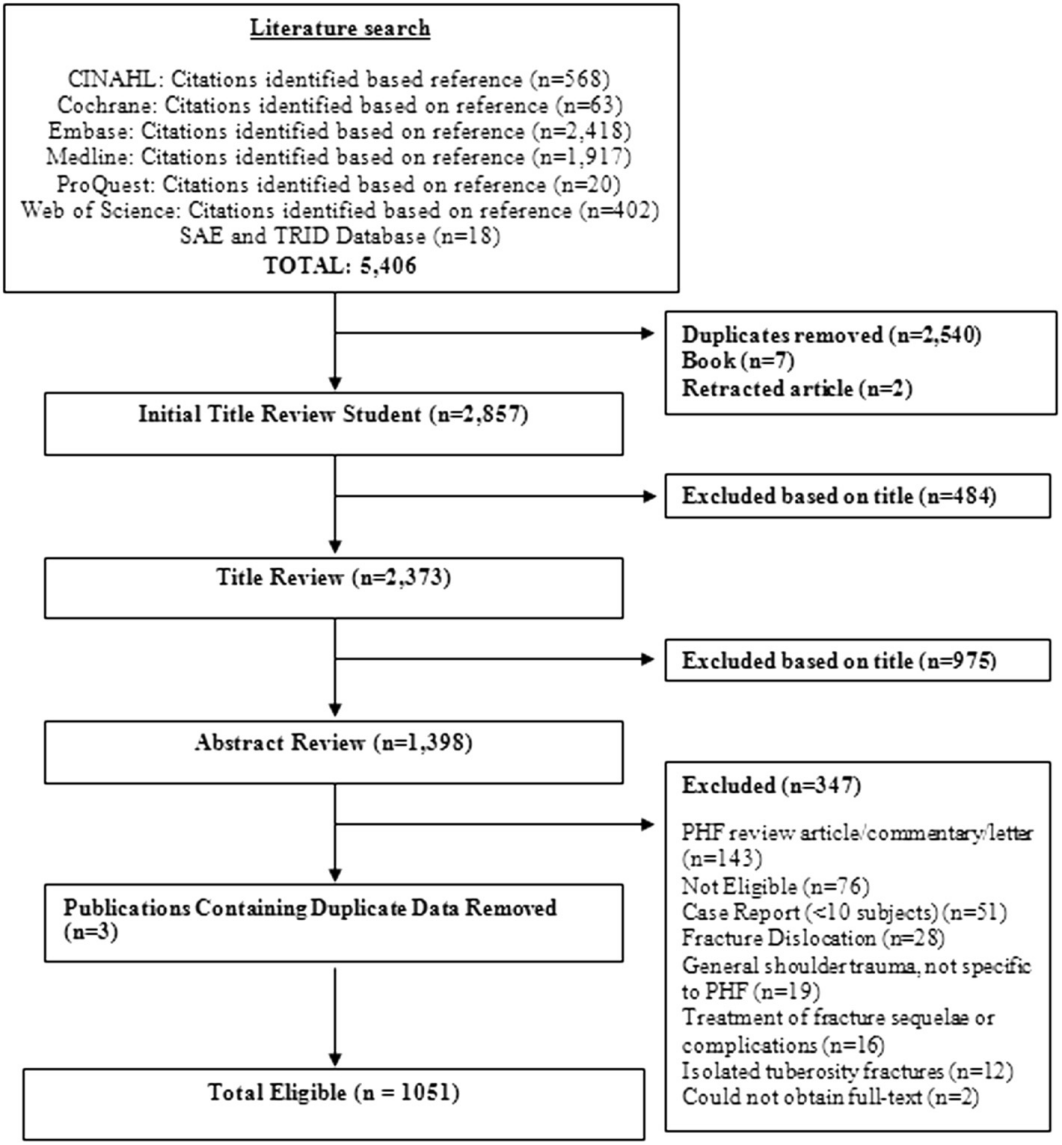
Literature search and screening flow chart.

The search strategy identified a total of 5,406 citations, of which 2,540 duplicates, 7 book titles, and 2 retracted publications were removed. An additional 1,459 titles were removed because they clearly did not meet our eligibility criteria. As a result, the abstracts of 1,398 publications were reviewed for further eligibility, and the final data set was comprised of 1,051 included studies (Figure 2 and Additional file 1).

**Figure 3:**
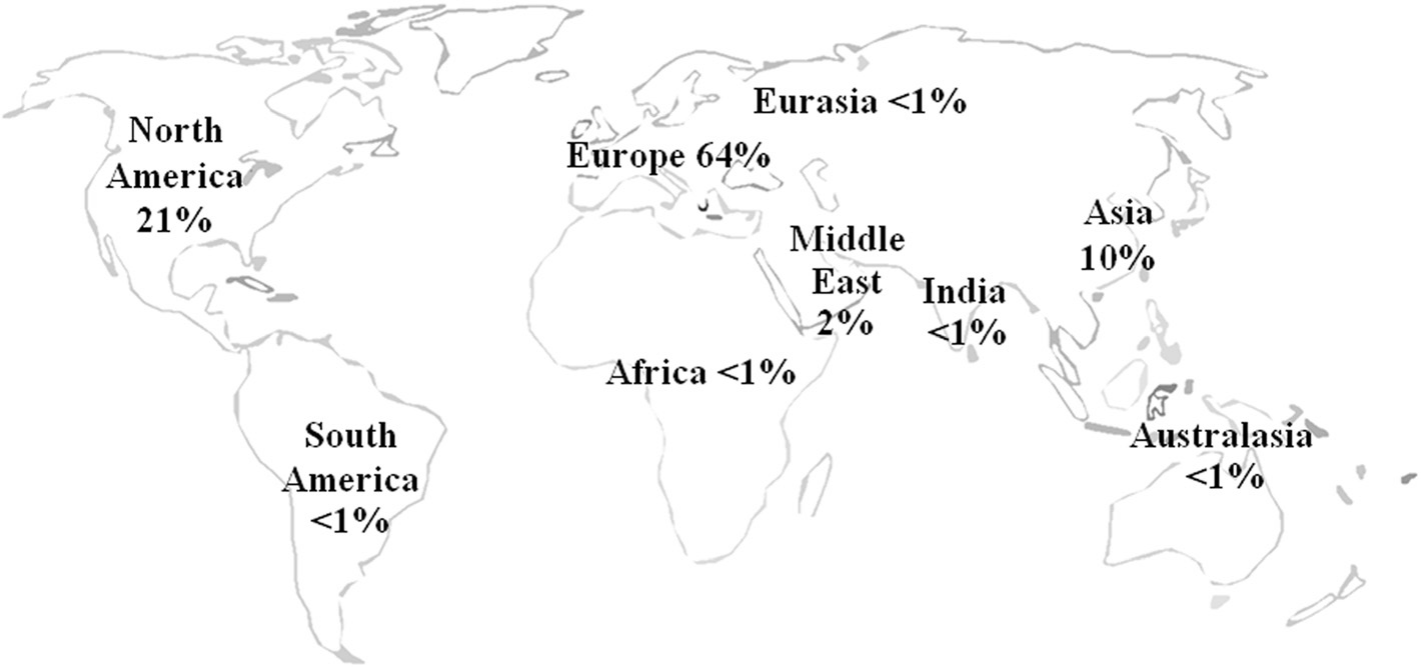
Location of research.

**Figure 4:**
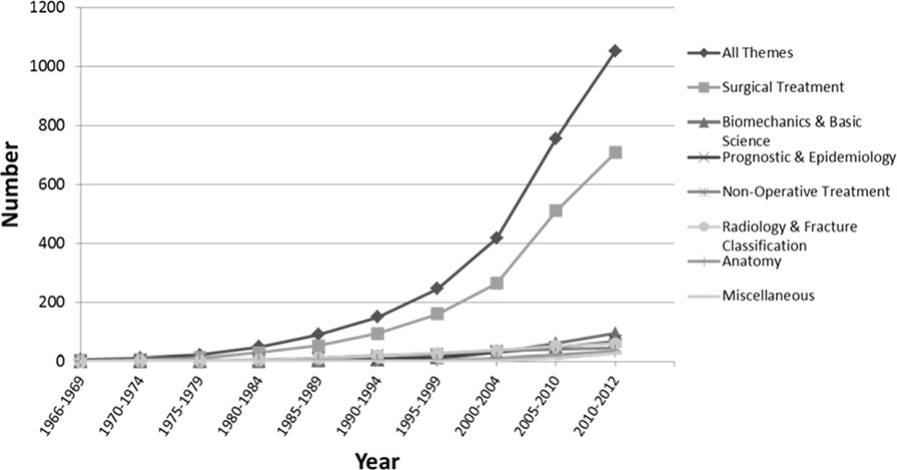
Cumulative number of publications by five year Increments and theme.

Substantial diversity was observed in the geographic location of study investigators and publication language (Table 2). The final data set included publications from all geographic regions; however, a predominance of European-led studies (64% of all titles) was observed (Figure 3). Included studies were published in 21 different languages, with English (72%), German (13%), and Chinese (5%) being the most common (Table 2). Publications from 208 different journals were included. Finally, an exponential increase in publications was observed in more recent years (Figure 4).

**Table 2:** **Study demographics**

**Characteristic**	**Frequency**
	**N (%)**
	**(N = 1,051)**
*Geographic location of study*	
Europe	673 (64.0)
North America	218 (20.7)
Asia	106 (10.1)
Middle East	21 (2.0)
India	8 (0.8)
Australasia	5 (0.5)
South America/Central America	5 (0.5)
Eurasia	3 (0.3)
Africa	2 (0.2)
International collaborations	10 (1.0)
*Publication language*	
English	752 (71.6)
German	134 (12.7)
Chinese	51 (4.9)
French	29 (2.8)
Italian	25 (2/4)
Czech	15 (1.4)
Polish	7 (0.7)
Russian	7 (0.7)
Turkish	6 (0.6)
Spanish	5 (0.5)
Danish	4 (0.4)
Greek	3 (0.3)
Portuguese	3 (0.3)
Slovak	3 (0.3)
Bulgarian	1 (0.1)
Croatian	1 (0.1)
Farsi	1 (0.1)
Hungarian	1 (0.1)
Japanese	1 (0.1)
Romanian	1 (0.1)
Serbian	1 (0.1)
*Study design*	
Case series	509 (48.4)
Comparative study	125 (11.9)
Basic science	123 (11.7)
Review	116 (11.0)
Surgical technique	56 (5.3)
Randomized controlled trial	33 (3.1)
Reliability	30 (2.9)
Incidence/Prevalence	20 (1.9)
Control	12 (1.1)
Protocol	5 (0.5)
Economic analysis	1 (0.1)
Survey	1 (0.1)
Other	9 (0.9)
Unknown	11 (1.0)
*Study perspective*	
Retrospective	411 (39.1)
Prospective	259 (24.6)
Not applicable	339 (32.3)
Unable to classify	42 (4.0)

### Study design

Several different study designs were identified among the included literature. Nearly half of the included proximal humerus fracture research was comprised of uncontrolled case series of a single treatment (n = 509, 48%) (Table 2). Non-randomized comparative studies represented 12% of the literature, and only 3% of the studies were randomized controlled trials. Although general review articles on the broad topic of shoulder trauma or proximal humerus fractures were excluded, review articles focusing on a single treatment or technique were included, and 116 studies (11%) fit this description. A similar proportion of basic science studies was also included (n = 123, 12%). Other study designs were used for non-therapeutic studies (<5%), such as surveys, reliability studies, and economic analyses. The perspective of the research question was assessed as prospective, retrospective, not applicable, or unable to assess. One third (32%) of the included were classified as not applicable and 4% were unable to be assessed. Of the clinical studies that could be classified as either prospective or retrospective (n = 670), 61% of these studies were retrospective and 39% were prospective designs.

### Study themes

**Figure 5:**
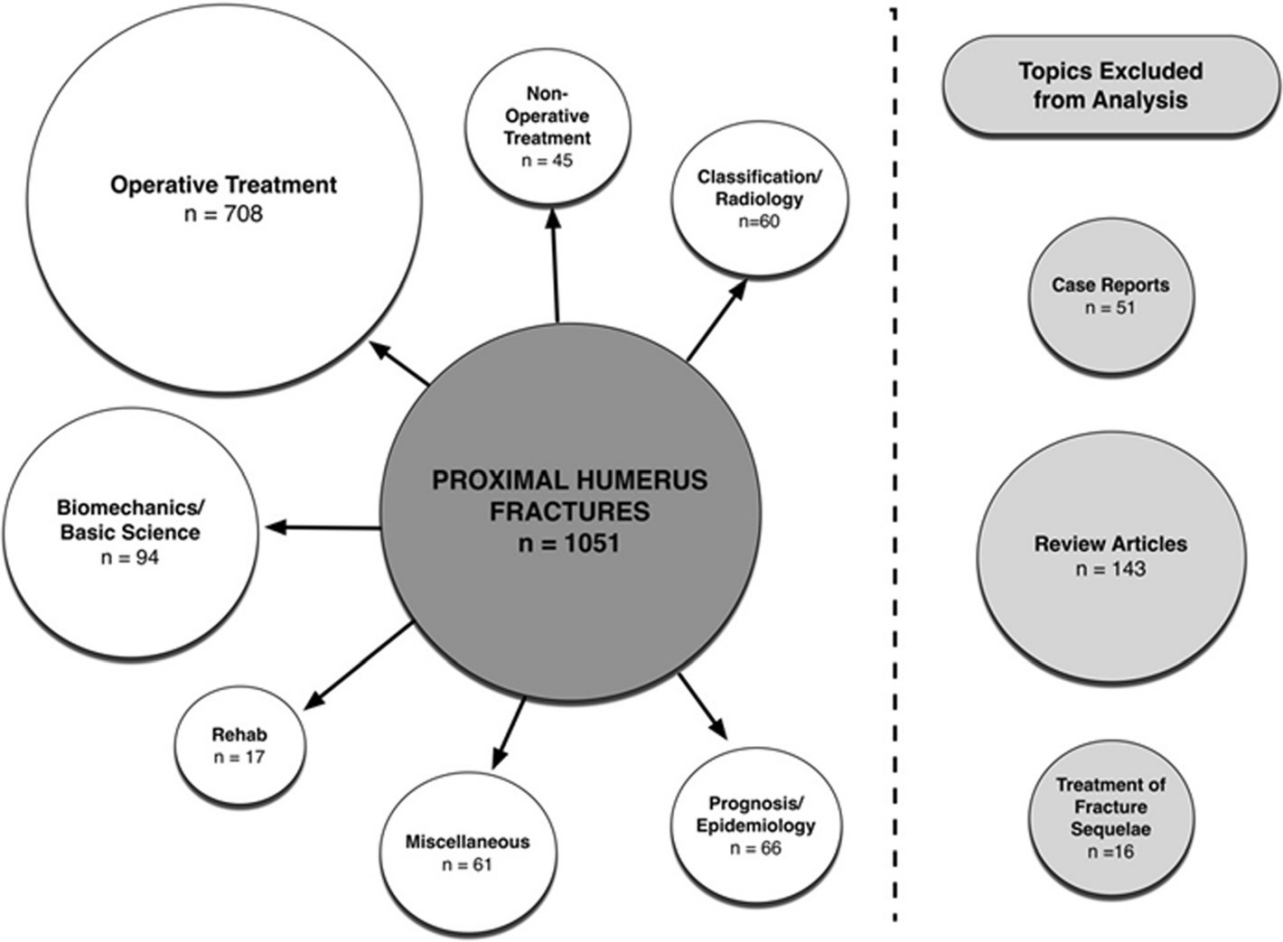
Proportion of proximal humerus fracture literature by theme.

Using the study themes created by our knowledge-user collaboration, each study was categorized in duplicate by two members of the data abstraction team. Moderate agreement was observed for determining a study’s theme, with a kappa statistic of 0.56. Figure 5 displays the concept map and proportions of publications per study theme.

Studies regarding the surgical treatment of proximal humerus fractures comprised over 65% of the included literature (n = 708). Biomechanical/basic science was the next most common study theme, but only comprised approximately 10% of the literature (n = 94). Interestingly, studies with a primary outcome examining the effectiveness of non-operative treatment or using a prognostic or epidemiological study design were uncommon (4% (n = 45) and 6% (n = 66) of total literature, respectively). Although excluded from the analysis, we also found that a large portion of the literature was comprised of general review articles, commentaries, and letters (n = 143) and case reports (n = 51).

### Theme specific results

The surgical theme contained the largest body of literature. Several observations were made about these studies. Most studies contained relatively small sample sizes, with a median of 35.0 patients (IQR 23.0 – 63.0) (Table 3). As seen in the overall body of literature, most studies were retrospective (47.6%, n = 337) and from European centers (63.8%, n = 452); furthermore, only 16% were comparative study designs (n = 113). Overall, randomized controlled trials comprised 3% of the surgical treatment literature (n = 21).

**Table 3:** **Sample size by study theme**

**Theme**	**Mean sample size**	**Median sample size**	**Inter quartile range**
Prognostic & Epidemiology	2,648.6	141	67.5-450.5
Miscellaneous	1,167.7	57	36-118.5
Non-operative	111.1	64.5	46.7-109.2
Radiology & Fracture classification	68.2	15.5	5-47.5
Rehabilitation	52.2	46.5	31-78.5
Surgical treatment	52.1	35	23-63
Anatomy	35.1	23	12-40
Biomechanics and Basic science	27.5	20	12-30

Within the biomechanics and basic science theme most studies were focused on biomechanical research questions. Of the 94 studies, 70% involved testing surgical implants in a cadaveric or artificial bone fracture model. Most of the implant testing was performed with plate fixation and often tested new implant designs. Eighty percent of these studies compared more than one treatment strategy, which facilitates comparative conclusions to be made; however, the heterogeneity in model designs can often make “bench to bedside” interpretations challenging.

The prognosis/epidemiology theme was comprised of studies with a primary research question aimed at the effect of a patient or injury characteristic on an outcome of interest. These studies had the largest sample sizes among all the themes with a median sample size of 141 patients (IQR 67.5 – 450.5); this is more than double the size of the themes with the next largest samples (Table 3). Larger sample sizes for this type of research question are important given their observational study designs. Similar to other themes, the majority of publications were from European centers and the data are limited by primarily retrospective studies.

Studies that focused on the non-operative management of proximal humerus fractures had much smaller sample sizes than the prognostic study designs; despite this, the sample size of non-operative studies was nearly twice as large as the surgical theme publications (median sample size 64.5 vs 35.0, respectively). The most noticeable difference between the surgical and non-operative studies is the proportional increase in studies published per year for each theme. Of the total number of surgical papers published, 45% were published in the last five years; whereas only 20% of the papers within the non-operative theme were published in the past 5 years.

Several other important theme-specific results were observed. The proximal humerus fracture specific rehabilitation literature, although small (n = 17), contains the largest proportion of randomized controlled trials (35%). This highlights an area of proximal humerus fracture management that is likely capable of making strong evidence-based treatment recommendations. The fracture classification and radiology theme primarily contained inter-observer reliability studies (40%) while the miscellaneous category contained a diverse sample of study designs including economic analyses, surveys, and clinical trial protocols.

## Discussion

This study represents the most comprehensive proximal humerus fracture literature review performed to date and uses a novel scoping review technique to map the entire breadth of relevant literature. Eight literature themes were proposed, and this thematic framework guided the literature analysis. Overall, we identified that over 65% of the literature consists of surgical treatment papers. Biomechanical studies and topic-specific review articles were the next most common themes in the literature (approximately 10% each). More importantly, we identified very few non-operative studies or prognostic studies that could provide appropriate treatment guidance, and the majority of studies were performed at European centers. Our key findings are:

- 1,051 eligible studies were identified.
- Studies from around the globe were identified and included.
- Approximately two thirds of the studies were conducted in Europe.
- Studies published in 21 different languages were included.
- Publications from 208 journals were included.
- Approximately half of the included studies are uncontrolled case series.
- Non-randomized comparative studies represented 12% of the literature.
- Only 3% of the studies were randomized controlled trials.
- The majority of the studies are retrospective in nature.
- Over two thirds of the included studies addressed surgical treatment.
- Few non-operative studies or prognostic studies that could provide important treatment guidance were identified.
- The proximal humerus fracture specific rehabilitation literature, although small, contains the largest proportion of randomized controlled trials.

The scoping review methodology was chosen for this review because the lack of clinical trials has repeatedly limited the utility of systematic reviews and meta-analyses since randomized controlled trials represent less than 1% of the proximal humerus fracture studies indexed in MEDLINE. As a result, the overwhelming majority of literature on this topic has not been summarized. Given the recurrent challenges of conducting a meta-analysis, [7] and the breadth of methodologies used in this field of research, a scoping review allowed us to map key areas of management and identify research gaps in the existing literature.

Similar to performing a focused systematic review, the search strategy and retrieval techniques for this scoping review required rigorous methodology and substantial personnel resources. A scoping review manages a much larger volume of citations than most focused systematic reviews. This was certainly seen in our review with individual study data abstracted from 1,051 citations in over 21 different languages. However, even with such a large variety in study theme, design, and language, we were able to abstract 97% of all data points.

Although scoping reviews are not common in the orthopaedic surgery literature, their use has been promoted by several government funding agencies and have been successfully employed in many other areas of medicine. Feehan et al. [12] performed a comprehensive scoping review of exercise prescription for older adults following a fragility fracture. A similar process of integrated knowledge translation was employed and a large group of academic researchers, physiotherapists, and consumer collaborators were involved in each step of the study. This iterative process ensures that all stakeholders can provide input on how to refine the research question, search strategy, and interpretation of study results in order to meet their individual knowledge needs. In the scoping review by Feehan and colleagues, they were able to identify important trends in the literature regarding hip fracture outcomes, as well as gaps in the literature particularly surrounding vertebral and upper extremity fragility fractures.

Our study extends the Feehan review by mapping the breadth of the proximal humerus fracture treatment literature. Since the treatment literature for proximal humerus fractures is rapidly expanding with several emerging techniques and implants, we chose to include all studies relevant to treating these common fragility fractures. Beyond gaining an understanding of the strengths and weaknesses of the current literature, this knowledge-user driven scoping review allowed us to identify other important knowledge-user needs to improve patient-centered care. For example, our orthopaedic surgeon knowledge users identified a need for more prospective studies, both prognostic and randomized controlled trials to better inform patient decision making. Similarly, our patient group representatives also identified a need for providing patients with lay summaries that can be used to empower a shared treatment decision process and to provide patients with more information regarding their rehabilitation and expected recovery. Finally, our biomechanical researchers identified a need to develop injury models that are more clinically relevant and that correspond to the challenging cases that clinicians face in their practice (i.e. comminuted 3- or 4-part fractures).

The data captured in the present review is capable of addressing many of these knowledge-user needs. Several strengths of the current proximal humerus fracture literature were identified. The largest portion of the literature involves surgical studies, and this is important given it is the intervention with the greatest patient risk. In addition, the surgical literature captured in the scoping review represents the available evidence for all operative interventions and can be used to advise patients, inform clinicians, and guide future research; despite this strength, the vast amount of surgical publications is also a reflection that surgical treatment strategies remain to be improved. Other strengths were found in the rehabilitation literature, which contained the highest proportion of randomized controlled trials within a theme (35%). Although this body of literature is quite small, readers can recognize the potential for strong evidence-based recommendations for the therapy of proximal humerus fracture patients. Now that we have a “map” of the management areas with the strongest available evidence, focused systematic reviews and possibly meta-analyses can be performed in order to guide treatment decisions.

Several other important observations regarding the existing literature were made. There is a relative lack of studies focusing on the outcomes of non-operative management and this is a noticeable weakness given that the overwhelming majority of proximal humerus fractures are treated without surgery (Table 4). Furthermore, prognostic studies that aim to determine important patient and injury characteristics that affect proximal humerus fracture outcomes are also significantly lacking. These results are likely worsened by our protocol to classify all studies into one of eight themes based on the study’s primary research objective. Several studies contained additional data that could have been classified into a different theme, and this information was not captured since there was substantial variability in the secondary research questions. As a result, this likely has underestimated the prevalence of some non-operative and prognostic data available. Finally, it is important to mention that our knowledge user group represented several clinical, research, and patient perspectives; however, it still lacked representation from key specialties such as endocrinology, rheumatology, and family medicine. Our knowledge user group has since grown to include representation from these fields in our subsequent research.

**Table 4:** **Future research solutions of identified gaps in the proximal humerus fracture literature**

**Gaps in the research literature**		**Future research solution**
• There is a lack of accessible knowledge for patients that can be used to empower a shared treatment decision process and provide patients with more information regarding their rehabilitation and expected recovery.	⇨	• Create and appropriately evaluate patient decision aids to translate the current knowledge into tools that patients can use to participate in their treatment decisions and better understand their injury and expected outcome.
• There is a great deal of literature on proximal humerus fractures, but a lack of systematic reviews addressing focused clinical questions.	⇨	• Focused systematic reviews to answer specific clinical questions.
• There is a lack of studies focusing on:		
1. Non-operative management, especially given that the overwhelming majority of proximal humerus fractures are treated without surgery.	⇨	• Conduct a series of planning meetings between clinicians, methodologists, and other knowledge users in order to prioritize and design future research studies that will address these gaps in knowledge for the treatment of proximal humerus fractures.
2. Prospective studies (both cohort studies and randomized controlled trials), as these study designs are key to informing patient decision making.		
3. Prognostic studies that aim to determine important patient and injury characteristics that affect proximal humerus fracture outcomes.		

## Conclusions

Overall, the results of the current study provide a comprehensive summary of the existing proximal humerus fracture literature using a thematic framework developed by a multi-disciplinary knowledge user collaboration. The key strengths and weaknesses identified have formed the roadmap for several future research directions (Table 4): 1) Using the existing proximal humerus fracture literature, we intend to create patient decision aids to translate the current knowledge into tools that patients can use to participate in their treatment decisions and to better understand their injury and expected outcome; 2) In areas identified with robust published data, we intend to perform focused systematic reviews to answer relevant clinical questions; 3) Biomechanical and basic science studies will be designed to develop models that address more clinically relevant fracture patterns; and 4) The scoping review summaries will be used as the focus of a series of planning meetings between clinicians, methodologists, and other knowledge users to prioritize and design future randomized controlled trials for the treatment of proximal humerus fractures.

## Additional file

Additional file 1:
**Listing of included studies.**

## Abbreviations

CDSR: Cochrane Database of Systematic Reviews
CINAHL: Cumulative Index of Nursing and Allied Health Literature
IQR: Interquartile Range
MeSH: Medical subject heading
SAE: Society of Automotive Engineers
TRID: Transport Research International Documentation
