# Supplementary material for: A scoping review of the proximal humerus fracture literature
Source: BMC Musculoskelet Disord. 2015 May 10;16:112. doi: 10.1186/s12891-015-0564-8 (PMC4464621; doi:10.1186/s12891-015-0564-8)
Supplement: Additional file 1: — Listing of included studies. [file 12891_2015_564_MOESM1_ESM.docx]

Additional file 1:

1. Chung H, Wai K, Chih T. Shoulder joint replacement of proximal humeral fractures. Zhonghua wai ke za zhi. 2007;45(20):1395.
2. Neer Score. J Orthop Trauma. 2006; 20(8): S128.
3. Abrutyn DA, Dines DM. Secure tuberosity fixation in shoulder arthroplasty for fractures. Tech Should Elb Surg. 2004; 5(4):177.
4. Accetta R, Aziz H, Mineo G. Locked plating: Clinical considerations. Tech Orthop. 2007; 22(3):156.
5. Ackermann C, Lam Q, Linder P, Kull C. Problems in classification of fractures of the proximal humerus. [Z Unfallchir Versicherungsmed Berufskr.](http://www.ncbi.nlm.nih.gov.libaccess.lib.mcmaster.ca/pubmed/3107242) 1986;79(4):209.
6. Acklin Y, Stoffel K, Sommer C. Clinical outcome after minimal invasive plate osteosynthesis for proximal humerus fractures. Br J Surg. 2011; 98:11.
7. Acklin YP, Jenni R, Walliser M, Sommer C. Minimal invasive PHILOS (R)-plate osteosynthesis of proximal humerus fractures Br J Surg. 2007; 94(6): 769.
8. Acklin YP, Sommer C. Plate fixation of proximal humerus fractures using the minimally invasive anterolateral delta split approach. Operative Orthopadie und Traumatologie. 2012. 24(1): 61.
9. Acklin YP, Jenni R, Walliser M, Sommer C. Minimal Invasive PHILOS (R)-Plate Osteosynthesis in Proximal Humeral Fractures. Eur J Trauma Emerg Surg. 2009; 35(1): 35.
10. Adedapo AO, Ikpeme JO. The results of internal fixation of three- and four-part proximal humeral fractures with the Polarus nail. Injury. 2001;32(2):115.
11. Agel J, Jones CB, Sonzone AG, Camuso M, Henley MB. Treatment of proximal humeral fractures with Polarus nail fixation. J Shoulder Elbow Surg. 2004; 13(2): 191.
12. Aggarwal S, Bali K, Dhillon MS, Kumar V, Mootha AK. Displaced proximal humeral fractures: an Indian experience with locking plates. J Orthop Surg Res. 2010; 5:60.
13. Agorastides I, Sinopidis C, Meligy M, Yin Q, Brownson P, Frostick SP. Early versus late mobilization after hemiarthroplasty for proximal humeral fractures. J Shoulder Elbow Surg. 2007; 16(3 Suppl): S33.
14. Agudelo J, Schurmann M, Stahel P, Helwig P, Morgan SJ, Zechel W, Bahrs C, Parekh A, Ziran B, Williams A, Smith W. Analysis of efficacy and failure in proximal humerus fractures treated with locking plates. J Orthop Trauma. 2007; 21(10):676.
15. Ahovuo J, Paavolainen P, Bjorkenheim JM. Fractures of the proximal humerus involving the intertubercular groove. Acta Radiol. 1989; 30(4):373.
16. Aksu N, Aslan O, Kara AN, Isiklar ZU. Simultaneous repair of chronic full-thickness rotator cuff tears during fixation of proximal humerus fractures and clinical results. Acta Orthop Traumatol Turc. 2010; 44(3):173.
17. Aksu N, Gogus A, Kara AN, Isiklar ZU. Complications encountered in proximal humerus fractures treated with locking plate fixation. Acta Orthop Traumatol Turc. 2010; 44(2): 89.
18. Alao U, Leonard M, Mokotedi L, Glynn A, Dolan M, Fleming P. The Use of Locking Plates in Proximal Humeral Fractures: Comparison of Outcome by Patient Age and Fracture Pattern. Ir J Med Sci. 2010; 179: S154.
19. Albritton MJ, Barnes CJ, Basamania CJ, Karas SG. Relationship of the axillary nerve to the proximal screws of a flexible humeral nail system: an anatomic study. J Orthop Trauma. 2003; 17(6):411.
20. Alexa O, Puha B, Veliceasa B, Popia I. Percutaneous pinning for proximal humerus fractures. Rev Med Chir Soc Med Nat Iasi. 2007; 111(1): 184.
21. Alt V, Persoons D. The BEHAC nail--a new intramedullary implant for managing 2-stage fractures of the humerus. Unfallchirurg. 2002; 105(5); 471.
22. Altay T, Karapinar L, Kaya A, Ozturk H. (2005). Treatment of two-part proximal humeral fractures with external fixators. TJTES. 2005;11(2); 153.
23. Altman GT, Gallo RA, Molinero KG, Muffly MT, Mascarenhas L. Minimally invasive plate osteosynthesis for proximal humerus fractures: functional results of treatment. Am J Orthop. 2011; 40(3); E40.
24. Amar MF, Almoubaker S, Chbani B, Benabid M, Lahrach K, Marzouki A, Boutayeb F. Kapandji pining in the treatment of proximal humerus fractures. Journal de Traumatologie du Sport. 2010: 27(4): 167.
25. Amirfeyz R, Sarangi P. Shoulder hemiarthroplasty for fracture with a conservative rehabilitation regime. Arch Orthop Trauma Surg. 2008;128(9): 985.
26. Ocana JA, Garcia MG, Herraez EM, Arias GR, Martin AP. Fractures of the proximal humerus. Review of therapeutic alternatives. Revista de Ortopedia y Traumatologia. 1997; 41(6):605.
27. Angehrn F, Schultheiss HR. (1986). Percutaneous drill wire osteosynthesis of fractures of the proximal humerus. Zeitschrift fur Unfallchirurgie .1986;79(3):155.
28. Angibaud L, Zuckerman JD, Flurin PH, Roche C, Wright T. Reconstructing proximal humeral fractures using the bicipital groove as a landmark. Clin Orthop Relat Res. 2007; 458:168.
29. Anjum SN, Butt MS. Treatment of comminuted proximal humerus fractures with shoulder hemiarthroplasty in elderly patients. Acta Orthop Belg. 2005; 71(4):388.
30. Antoljak T, Hancevic J, Zagar Z, Haiman M, Nikolic V, Hudec M, Davila S, Barila-Antoljak N. Influence of bone properties in originating proximal humeral fractures. Period Biol. 1993; 95(1):213.
31. Antuna SA, Sperling JW, Cofield RH. Shoulder hemiarthroplasty for acute fractures of the proximal humerus: a minimum five-year follow-up. J Shoulder Elbow Surg. 2008; 17(2): 202.
32. Aprato A, Ferro L, Maderni A, Sisto R, Masse A. Internal fixation of displaced proximal humeral fractures with Locking Compression Plate. Minerva Ortopedica e Traumatologica. 2005; 56(6): 483.
33. Aschauer E, Resch H, Hubner C. Percutaneous osteosynthesis of humeral head fractures. Operative Orthopadie und Traumatologie. 2007; 19(3): 276.
34. Assom M, Blonna D, Fantino G, Bruzzone M, Rossi R, Castoldi F. Hemiarthroplasty in the treatment of proximal humeral fractures. Minerva Ortopedica e Traumatologica . 2006; 57(4):313.
35. Assom M, Fumero S, Castoldi F, Blonna D. Hemiarthroplasty in the treatment of proximal humeral fractures. Minerva Ortopedica e Traumatologica. 2005; 56(4): 315.
36. Astrand J. Proximal humerus fractures in patients between 50 and 65 years cause inferior subjective outcome. Stronger indication for active surgical treatment? Calcif Tissue Int. 2007; 80: S115
37. Atalar AC, Demirhan M,Uysal M, Seyahi A. Treatment of Neer type 4 impacted valgus fractures of the proximal humerus with open reduction, elevation, and grafting. Acta Orthopaedica et Traumatologica Turcica. 2007;41(2):113.
38. Athwal GS, MacDermid JC, Goel DP. Metaversion can reliably predict humeral head version: a computed tomography-based validation study. J Shoulder Elbow Surg. 2010;19(8):1145.
39. Audren JL. Osteosynthesis of fractures of thr proximal end of the humerus by the screw-pin technique. J Chir.1992;129(10):444.
40. Babhulkar A, Shyam AK, Sancheti PK, Shah K, Rocha S. Hemiarthroplasty for comminuted proximal humeral fractures. J Orthop Surg. 2011;19(2):194.
41. Babst R, Brunner F. Plating in proximal humeral fractures Eur. J Trauma Emerg Surg. 2007;33(4):345.
42. Badman B, Frankle M, Keating C, Henderson L, Brooks J, Mighell M. Results of proximal humeral locked plating with supplemental suture fixation of rotator cuff. J Shoulder Elbow Surg. 2011; 20(4):616.
43. Badman BL, Mighell M. Fixed-angle locked plating of two-, three-, and four-part proximal humerus fractures. J Am Acad Orthop Surg. 2008;16(5):294.
44. Bae JH, Oh JK, Chon CS, Oh CW, Hwang JH, Yoon YC. The biomechanical performance of locking plate fixation with intramedullary fibular strut graft augmentation in the treatment of unstable fractures of the proximal humerus. JBJS (Br). 2011;93(7):937.
45. Bahrs C, Oehm J, Rolauffs B, Eingartner C, Weise K, Dietz K, Helwig P. T-plate osteosynthesis - An obsolete osteosynthesis procedure for proximal humeral fractures? Middle-term clinical and radiological results. Z Orthop Unfallchir. 2007; 145(2):186.
46. Bahrs C, Rolauffs B, Stuby F, Dietz K, Weise K, Helwig P. Effect of proximal humeral fractures on the age-specific prevalence of rotator cuff tears. J Trauma-Injury Infect Crit Care. 2010;69(4):901.
47. Bahrs C, Badke A, Rolauffs B, Weise K, Zipplies S, Dietz K,Eingartner C. Long-term results after non-plate head-preserving fixation of proximal humeral fractures. Int Orthop. 2010;34(6):883.
48. Bahrs C, Rolauffs B, Dietz K, Eingartner C, Weise K. Clinical and radiological evaluation of minimally displaced proximal humeral fractures. Arch Orthop Trauma Surg. 2010; 130(5):673.
49. Bahrs C, Rolauffs B, Suedkamp NP, Schmal H, Eingartner C, Dietz K, Pereira PL, Weise K, Lingenfelter E, Helwig P. Indications for computed tomography (CT-) diagnostics in proximal humeral fractures: a comparative study of plain radiography and computed tomography. BMC Musculoskelet Disord. 2009;10:33.
50. Bahrs C, Schmal H, Lingenfelter E, Rolauffs B, Weise K, Dietz K, Helwig P. Inter- and intraobserver reliability of the MTM-classification for proximal humeral fractures: A prospective study. BMC Musculoskelet Disord. 2008;9:21.
51. Bai L, Wang TB, Zhang PX, Wang J, Xu HL, Xue F, Chen JH, Dang Y, Yang M, Xiong J, Fu ZG, Zhang DY, Zhang HB, Wang G, Shen HL, Wang GL, Wu XB, Jiang BG. Three kind of scoring system for proximal humeral fractures in patients with postoperative functional review of evaluation: a multicenter study. Chung-Hua Wai Ko Tsa Chih. 2012; 50(4):318.
52. Baker P, Nanda R, Goodchild L, Finn P, Rangan A. A comparison of the Constant and Oxford shoulder scores in patients with conservatively treated proximal humeral fractures. J Shoulder Elbow Surg. 2008;17(1):37.
53. Balg F, Boulianne M, Boileau P. Bicipital groove orientation: considerations for the retroversion of a prosthesis in fractures of the proximal humerus. J Shoulder Elbow Surg. 2006; 15(2):195.
54. Ballmer FT, Hertel R. Indications and results of shoulder prosthetics in complex proximal humerus fractures. Universitatsklinik fur Orthopadische Chirurgie. 1998;55(3):197.
55. Baltensweiler J. Cluster nailing of the proximal humeral metaphysis. Schweizerische Rundschau fur Medizin Praxis. 1984;73(4):111.
56. Bao HW, Wang Q. Anatomical research on proximal humeral fracture treated with humeral head replacement. Chung-Hua i Hsueh Tsa Chih. 2010; 90(45):3217.
57. Baranovic S, Bencic I, Durdevic D, Maldini B, Milosevic M. A comparison of interscalene block anaesthesia and general anaesthesia in patients with proximal humerus fracture. Periodicum Biologorum. 2011; 113:31.
58. Barlow JD, Sanchez-Sotelo J, Torchia M. Proximal humerus fractures in the elderly can be reliably fixed with a "hybrid" locked-plating technique. CORR. 2011; 469(12): 3281.
59. Bartonicek J, Stehlik J, Dlouhy M. Modified semitubular blade plate for fixation of proximal humerus fractures part I: surgical technique. Acta Chir Orthop Traumatol Cech. 1995;62(4):207.
60. Bartsch S, Hullmann S, Hillrichs B, Giers R, Echtermeyer V. Osteosynthesis of dislocated proximal humerus fractures by an angular stable T-plate. Progress in treatment of this problematical fracture? Implant, technical procedures and first experiences. Aktuelle Traumatol. 2001; 31(2): 64.
61. Barvencik F, Gebauer M, Beil FT, Vettorazzi E, Mumme M, Rupprecht M, Pogoda P, Wegscheider K, Rueger JM, Pueschel K, Amling M. Age- and sex-related changes of humeral head microarchitecture: histomorphometric analysis of 60 human specimens. J Orthop Res. 2010; 28(1):18.
62. Bastian JD, Hertel R. Osteosynthesis and hemiarthroplasty of fractures of the proximal humerus: outcomes in a consecutive case series. J Shoulder Elbow Surg. 2009;18(2):216.
63. Bastian JD, Hertel R. Initial post-fracture humeral head ischemia does not predict development of necrosis. J Shoulder Elbow Surg. 2008;17(1):2.
64. Bastlova P, Krobot A, Mikova M, Skoumal P, Freiwald J. Strategy of Rehabilitation after Fractures of Proximal Humerus. Rehabilitace a Fyzikalni Lekarstvi. 2004; 11(1): 3.
65. Bathis H, Tingart M, Bouillon B, Tiling T. Surgical treatment of proximal humeral fractures. Is the T-plate still adequate osteosynthesis procedure? Zentralbl Chir. 2001;126(3):211.
66. Battistella F, Oldani M, Tajana MS, Prestamburgo D.Treatment of proximal humerus fractures with locking plate by minimally invasive technique. J Orthop Trauma. 2011;12:S15.
67. Bauer O, Horvath I, Kelemen P, Hepp B. Follow-up our patients with proximal humeral fractures between 2000 and 2005. Orv Hetil. 2009;150(49):2237.
68. Baumgartner D, Nolan BM, Mathys R, Lorenzetti SR, Stussi E. Review of fixation techniques for the four-part fractured proximal humerus in hemiarthroplasty. J Orthop Surg Res. 2011;6:36.
69. Baumgartner D, Lorenzetti SR, Mathys R, Gasser B, Stuessi E. Refixation stability in shoulder hemiarthroplasty in case of four-part proximal humeral fracture. Med Biol Eng Comput. 2009; 47(5):515.
70. Becker R, Pap G, Machner A, Neumann WH. Strength and motion after hemiarthroplasty in displaced four-fragment fracture of the proximal humerus: 27 patients followed for 1-6 years. Acta Orthop Scand. 2002;73(1):44.
71. Bell E, Leung BC, Spratt KF, Koval KJ, Weinstein JD, Goodman DC, Tosteson ANA. Trends and variation in incidence, surgical treatment, and repeat surgery of proximal humeral fractures in the elderly. JBJS. 2011; 93(2):121.
72. Bellato E, Scelsi M, Marini E, Mortera S, Marenco S, Blonna D, Assom M, Castoldi F. Complex Proximal Humeral Fractures: Hemiarthroplasty Versus Percutaneous Osteosynthesis. Minerva Ortop Traumatol. 2011;62(1): 19.
73. Bengard MJ, Gardner MJ. Screw depth sounding in proximal humerus fractures to avoid iatrogenic intra-articular penetration. J Orthop Trauma. 2011; 25(10):630.
74. Bengert O. The elastic esmarch bandage, an appropriate means in the treatment of proximal fractures of the humerus? Orthopadische Praxis. 1990; 26(3):193.
75. Bernard J, Charalambides C, Aderinto J, Mok D. Early failure of intramedullary nailing for proximal humeral fractures. Injury. 2000; 31(10): 789.
76. Bernstein J, Adler LM, Blank JE, Dalsey RM, Williams GR, Iannotti JP. Evaluation of the Neer system of classification of proximal humeral fractures with computerized tomographic scans and plain radiographs. JBJS. 1996; 78(9):1371.
77. Bertoft ES, Lundh I, Ringqvist I. Physiotherapy after fracture of the proximal end of the humerus. Comparison between two methods. Scand J Rehabil Med. 1984;16(1):11.
78. Besch L, Daniels-Wredenhagen M, Mueller M, Varoga D, Hilgert E, Seekamp A. Hemiarthroplasty of the shoulder after four-part fracture of the humeral head: a long-term analysis of 34 cases. J Trauma. 2009;66(1):211.
79. Bhandari M, Matthys G, McKee MD, Evidence-Based Orthopaedic Trauma Working Group. Four part fractures of the proximal humerus. J Orthop Trauma. 2004;18(2):126.
80. Bhatia DN. van Rooyen KS, du Toit DF, de Beer JF. Surgical treatment of comminuted, displaced fractures of the greater tuberosity of the proximal humerus: a new technique of double-row suture-anchor fixation and long-term results. Injury. 2006; 37(10):946.
81. Bian PT, Bian J. Treatment of surgical neck fracture of the humerus with percutaneous reduction of proximal part. Zhongguo Gushang. 2008; 21(1):68.
82. Biasibetti A, Maderni A, Aprato A, Sisto R, Masse A. Fixation of displaced proximal humeral fractures with locking compression plate. Minerva Ortopedica e Traumatologica. 2006;57(4):305.
83. Bicknell RT, DeLude JA, Kedgley AE, Ferreira LM, Dunning CE, King GJ, Faber KJ, Johnson JA, Drosdowech DS. Early experience with computer-assisted shoulder hemiarthroplasty for fractures of the proximal humerus: development of a novel technique and an in vitro comparison with traditional methods. J Shoulder Elbow Surg. 2007;16(3 Suppl): S117.
84. Bigliani LU, McCluskey GM. Prosthetic replacement in acute fractures of the proximal humerus. Semin Arthroplasty. 1990;1(2):129.
85. Bigorre N, Talha A, Cronier P, Hubert L, Toulemonde JL, Massin P. A prospective study of a new locking plate for proximal humeral fracture. Injury. 2009;40(2): 192.
86. Bishop J, Flatow EL. An alternative to replacement for complex proximal humerus fractures. Semin Arthroplasty. 2003;14(1):17.
87. Bjorkenheim JM, Pajarinen J, Savolainen V. Internal fixation of proximal humeral fractures with a locking compression plate - A retrospective evaluation of 72 patients followed for a minimum of 1 year. Acta Orthop Scand. 2004;75(6): 741.
88. Blonna D, Fantino G, Assom M, Dettoni F, Rossi R, Castoldi F. The treatment of the proximal humeral fractures with percutaneous Kirschner wires. Minerva Ortopedica e Traumatologica. 2006; 57(4):381.
89. Blonna D, Castoldi F, Scelsi M, Rossi R, Falcone G, Assom M. The hybrid technique: Potential reduction in complications related to pins mobilization in the treatment of proximal humeral fractures J.Shoulder Elbow Surg. 2010; 19(8):1218.
90. Blonna D, Rossi R, Fantino G, Maiello A, Assom M, Castoldi F. The impacted varus (A2.2) proximal humeral fracture in elderly patients: Is minimal fixation justified? A case control study J.Shoulder Elbow Surg. 2009;18(4):545.
91. Blum J, Hansen M, Rommens PM. Angle-stable intramedullary nailing of proximal humerus fractures with the PHN (proximal humeral nail). Operative Orthopadie und Traumatologie. 2009;21(3):296.
92. Blum J, Hessmann MH, Rommens PM. Humeral nailing with spiral blade in the treatment of proximal diaphyseal and metaphyseal fractures. Physikalische Medizin Rehabilitationsmedizin Kurortmedizin. 2005; 15(2):90.
93. Blum J, Hessmann MH, Rommens PM. Treatment of proximal metaphyseal fractures of the humerus with interlocked nailing and a spiral blade - Early experience with a new implant system. Aktuelle Traumatol. 2003; 33(1):7.
94. Blum J, Rommens PM. Proximal interlocking of humeral intramedullary nails and risk of axillary nerve injury. Unfallchirurg. 2002;105(1):9.
95. Blum J, Hansen M, Mueller M, Rommens PM, Matuschka H, Olmeda A, Radziejowski MJ, Merbold W, Nijs S, Angeloni R. Proximal Humeral Fractures and Intramedullary Nailing: Experience with a New Nail System. Eur J Trauma Emerg Surg. 2009 35(5):489.
96. Bogner R, Hubner C, Matis N, Auffarth A, Lederer S, Resch H. Minimally-invasive treatment of three- and four-part fractures of the proximal humerus in elderly patients. JBJS (Br). 2008;90(12):1602.
97. Boileau P, Caligaris-Cordero B, Payeur F, Tinsi L, Argenson C. Prognostic factors during rehabilitation after shoulder prostheses for fracture. Revue de Chirurgie Orthopedique et Reparatrice de l Appareil Moteur. 1999; 85(2):106.
98. Boileau P, Krishnan SG, Tinsi L, Walch G, Coste JS, Mole D. Tuberosity malposition and migration: reasons for poor outcomes after hemiarthroplasty for displaced fractures of the proximal humerus. J Shoulder Elbow Surg. 2002;11(5):401.
99. Boileau P, Pennington SD, Alami G. Proximal humeral fractures in younger patients: fixation techniques and arthroplasty. J Shoulder Elbow Surg. 2011;20(2 Suppl):S47.
100. Bonaspetti G, Fenu R, Bono S, Veneziani S, Pazzaglia UE. Treatment of proximal metadiaphyseal humeral fractures with Marchetti-Vicenzi intramedullar elastic nail. Minerva Ortopedica e Traumatologica. 2001;52(4-5):195.
101. Bondi R, Ceccarelli E, Campi S, Padua R. Shoulder arthroplasty for complex proximal humeral fractures. J Orthop Trauma. 2005;6(1):57.
102. Bosch U, Fremerey RW, Skutek M, Lobenhoffer P, Tscherne H. Hemi-arthroplasty--primary or secondary measure for 3- and 4-fragment fractures of the proximal humerus in the elderly? Unfallchirurg. 1996;99(9):656.
103. Bosch, Skutek M, Fremerey RW, Tscherne H. Outcome after primary and secondary hemiarthroplasty in elderly patients with fractures of the proximal humerus. J Shoulder Elbow Surg. 1998;7(5):479.
104. Boselli KJ, Bigliani LU. Four-part fractures require replacement: Once is enough-affirms. Semin Arthroplasty. 2010;21(3):155.
105. Boughebri O, Havet E, Sanguina M, Daumas L, Jacob P, Zerkly B, Heissler P. Treatment of proximal humeral fractures by Telegraph (R) nail: prospective study of 34 cases. Rev Chir Orthop Reparatrice Appareil Moteur. 2007; 93(4):325.
106. Braman JP, Flatow EL. How to transition to percutaneous pinning for proximal humerus fractures. Tech Should Elb Surg. 2005;6(3):171.
107. Braman JP, Flatow EL. Decision making in difficult proximal humerus fractures: When to fix, pin, or replace. Semin Arthroplasty. 2004;15(4 Spec.Iss):215.
108. Brems JJ. Shoulder arthroplasty in the face of acute fracture: puzzle pieces. J Arthroplasty. 2002;17(4 Suppl 1):32.
109. Brianza S, Plecko M, Gueorguiev B, Windolf M, Schwieger K. Biomechanical evaluation of a new fixation technique for internal fixation of three-part proximal humerus fractures in a novel cadaveric model. Clin Biomech. 2010;25(9):886.
110. Brianza S, Roderer G, Schiuma D, Schwyn R, Scola A, Gebhard F, Tami AE. Where do locking screws purchase in the humeral head? Injury. 2012;43(6):850.
111. Brien H, Noftall F, MacMaster S, Cummings T, Landells C, Rockwood P. Neer's classification system: a critical appraisal. J Trauma Injury Infect Crit Care . 1995;38(2):257.
112. Bries AD, Bell RH. Four-Part Fractures of the Proximal Humerus: Open Reduction and Internal Fixation versus Hemiarthroplasty. Semin Arthroplasty. 2008;19(1):45.
113. Brooks CH, Revell WJ, Heatley FW. Vascularity of the humeral head after proximal humeral fractures. An anatomical cadaver study. JBJS (Br). 1993;75(1):132.
114. Brorson S, Bagger J, Sylvest A, HrÃ³bjartsson H. Improved interobserver variation after training of doctors in the Neer system: a randomised trial. JBJS (Br). 2002;84B(7):950.
115. Brorson S, Bagger J, Sylvest A, Hrobjartsson A. Diagnosing displaced four-part fractures of the proximal humerus: A review of observer studies. Int Orthop. 2009; 33(2): 323.
116. Brorson S, Bagger J, Sylvest A, Hrobjartsson A. Low agreement among 24 doctors using the Neer-classification; only moderate agreement on displacement, even between specialists. Int Orthop. 2002;26(5):271.
117. Brorson S, Frich LH, Winther A, Hrobjartsson A. Locking plate osteosynthesis in displaced 4-part fractures of the proximal humerus. Acta Orthopaedica. 2011; 82(4):475.
118. Brorson S, Hrobjartsson A. Training improves agreement among doctors using the Neer system for proximal humeral fractures in a systematic review. J Clin Epidemiol. 2008;61(1):7.
119. Brorson S, Olsen BS, Frich LH, Jensen SL, Johannsen HV, Sorensen AK, Hrobjartsson A. Effect of osteosynthesis, primary hemiarthroplasty, and non-surgical management for displaced four-part fractures of the proximal humerus in elderly: a multi-centre, randomised clinical trial. Trials. 2009; 10:51.
120. Brorson S, Rasmussen JV, Frich LH, Olsen BS, Hrobjartsson A. Benefits and harms of locking plate osteosynthesis in intraarticular (OTA Type C) fractures of the proximal humerus: a systematic review. Injury. 2012; 43(7):999.
121. Bruder A, Taylor NF, Dodd KJ, Shields N. Exercise reduces impairment and improves activity in people after some upper limb fractures: a systematic review. J Physiother. 2011;57(2):71.
122. Brunner A, Honigmann P, Treumann T, Babst R. The impact of stereo-visualisation of three-dimensional CT datasets on the inter- and intraobserver reliability of the AO/OTA and Neer classifications in the assessment of fractures of the proximal humerus. JBJS (Br). 2009; 91(6):766.
123. Brunner A, Thormann S, Babst R. Minimally invasive plating osteosynthesis of proximal humeral shaft fractures with long PHILOS plates. Operative Orthopadie und Traumatologie. 2012;24(4-5):302.
124. Brunner A, Thormann S, Babst R. Minimally invasive percutaneous plating of proximal humeral shaft fractures with the Proximal Humerus Internal Locking System (PHILOS). J Shoulder Elbow Surg. 2012; 21(8):1056.
125. Brunner A, Weller K, Thormann S, Jockel JA, Babst R. Closed reduction and minimally invasive percutaneous fixation of proximal humerus fractures using the Humerusblock. J Orthop Trauma. 2010;24(7):407.
126. Brunner A, Resch H, Babst R, Kathrein S, Fierlbeck J, Niederberger A, Schmoelz W. The Humerusblock NG: a new concept for stabilization of proximal humeral fractures and its biomechanical evaluation. Arch Orthop Trauma Surg. 2012;132(7):985.
127. Brunner F, Sommer C, Bahrs C, Heuwinkel R, Hafner C, Rillmann P, Kohut G, Ekelund A, Muller M, Audige L, Babst R. Open reduction and internal fixation of proximal humerus fractures using a proximal humeral locked plate: a prospective multicenter analysis. J Orthop Trauma. 2009;23(3):163.
128. Budassi P, Stroppa S.Early results in osteosynthesis of fractures of the proximal humerus with a radiolucent plate of Peek. J Orthop Trauma. 2011;12:S99.
129. Bufquin T, Hersan A, Hubert L, Massin P. Reverse shoulder arthroplasty for the treatment of three- and four-part fractures of the proximal humerus in the elderly: a prospective review of 43 cases with a short-term follow-up. JBJS (Br). 2007;89(4):516.
130. Bungaro P, Pascarella R, Rollo G, Gennaro GL, Ercolani C. Osteosynthesis with percutaneous wiring in fractures of the proximal humerus. Chirurgia Degli Organi di Movimento. 1998; 83(4):381.
131. Burke NG, Kennedy J, Green C, Dodds MK, Mullett H. Locking Plate Fixation for Proximal Humerus Fractures. Orthopedics. 2012;35(2):E250.
132. Burkhead JR. Use of porous-coated modular prosthesis in the treatment of complex fractures of the proximal humerus. Tech Orthop. 1993;8(3):184.
133. Burri C, Rueter A, Spier W. Endoprostheses for the shoulder joint. Aktuelle Traumatol. 1977;7(3):155.
134. Burton J, Wells G, Watters A, Schilders E, Venkateswaran B. Early experience with the PlantTan Fixator Plate for 2 and 3 part fractures of the proximal humerus. Injury. 2005;36(10):1190.
135. Cai M, Tao K, Yang C, Li S.Internal Fixation Versus Shoulder Hemiarthroplasty for Displaced 4-part Proximal Humeral Fractures in Elderly Patients. Orthopedics. 2012;35(9):E1340.
136. Caira SF, Melanotte PL. Hemiarthroplasty of the shoulder in complex fractures of the proximal humerus. Minerva Ortopedica e Traumatologica. 1997;48(7-8):319.
137. Calvisi V, Morico G, Nasi M, Caruccio P, Romanini L. Current trends in conservative treatment of fractures of the proximal humerus. Ital J Orthop Traumatol.1991;17(2):179.
138. Calvo E, Morcillo D, Foruria AM, Redondo-Santamaria E, Osorio-Picorne F, Caeiro JR. Nondisplaced proximal humeral fractures: High incidence among outpatient-treated osteoporotic fractures and severe impact on upper extremity function and patient subjective health perception. J Shoulder Elbow Surg. 2011; 20(5):795.
139. Calvo E, de Miguel I, de la Cruz JJ, Lopez-Martin N. Percutaneous fixation of displaced proximal humeral fractures: Indications based on the correlation between clinical and radiographic results. J Shoulder Elbow Surg. 2007;16(6):774.
140. Carbone S, Tangari M, Gumina S, Postacchini R, Campi A, Postacchini F. Percutaneous pinning of three- or four-part fractures of the proximal humerus in elderly patients in poor general condition: MIROS[REGISTERED] versus traditional pinning. Int Orthop. 2012; 36(6):1267.
141. Carlan D, Pratt J, Patterson JM, Weiland AJ, Boyer MI, Gelberman RH. The radial nerve in the brachium: an anatomic study in human cadavers. J Hand Surg. 2007; 32(8):1177.
142. Carrera EF, Nicolao FA, Netto NA, Carvalho RL, dos Reis FB, Giordani EJ. A mechanical comparison between conventional and modified angular plates for proximal humeral fractures. J Shoulder Elbow Surg. 2008; 17(4):631.
143. Casals A, Amat J, Frigola M, Rodriguez-Cheu LE, Torrens C, Gines A. Monitoring and robotizing shoulder arthroplasty for training and optimization of suturing techniques. Int J Comput Assist Radiol Surg. 2008;3(1-2):61.
144. Castagno AA, Shuman WP, Kilcoyne RF, Haynor DR, Morris ME, Matsen FA. Complex fractures of the proximal humerus: role of CT in treatment. Radiology. 1987;165(3):759.
145. Castelli PG, Boschetti A, Bagnasacco A. Fractures of the proximal epiphysis of the humerus. Treatment with polarus nail. Minerva Ortopedica e Traumatologica. 2002;53(2):60.
146. Castoldi F, Bonasia DE, Blonna D, Rossi R, Dettoni F, Assom M, Sankey A, Halewood C, Amis AA. The Stability of Percutaneous Fixation of Proximal Humeral Fractures. JBJS. 2010;92A:90.
147. Castricini R, De Benedetto M, Pirani P, Panfoli N, Pace N. Shoulder hemiarthroplasty for fractures of the proximal humerus. Musculoskelet Surg. 2011;95(Suppl 1):S49.
148. Cazeneuve JF, Cristofari DJ. The reverse shoulder prosthesis in the treatment of fractures of the proximal humerus in the elderly. JBJS (Br). 2010; 92(4):535.
149. Cazeneuve JF, Cristofari DJ. Delta III reverse shoulder arthroplasty: radiological outcome for acute complex fractures of the proximal humerus in elderly patients. Orthop Traumatol Surg Res. 2009; 95(5):325.
150. Cazeneuve JF, Cristofari DJ. Grammont reversed prosthesis for acute complex fracture of the proximal humerus in an elderly population with 5 to 12 years follow-up. Revue de Chirurgie Orthopedique et Reparatrice de l Appareil Moteur. 2006; 92(6):543.
151. Cazeneuve JF, Hassan Y, Kermad F, Brunel A. Delta III reverse-ball-and-socket total shoulder prosthesis for acute complex fractures of the proximal humerus in elderly population. Eur J Orthop Surg Traumatol. 2008;18(2):81.
152. Cenna E, Comba D, Graziano E, Anselmi P, Morino L. Shoulder endoprosthesis in complex proximal humeral fractures. Minerva Ortopedica e Traumatologica. 1998;49(6):189.
153. Ceretti M, Pappalardo S, Fanelli M. Humeral fractures osteosynthesis in osteoporotic patient: TGF versus K wires fixation. J Orthop Trauma. 2011;12:S19.
154. Chaix O, Le Balc'H T, Mazas F. Fractures of the proximal humerus. Classification and treatment indications. Ann Chir.1984;38(3):220.
155. Chandler RW. Displaced proximal humeral fractures treated with a locked intramedullary device. Tech Orthop. 1993; 8(3):192.
156. Charalambous CP, Siddique I, Valluripalli K, Kovacevic M, Panose P, Srinivasan M, Marynissen H. Proximal humeral internal locking system (PHILOS) for the treatment of proximal humeral fractures. Arch Orthop Trauma Surg. 2007; 127(3):205.
157. Chen CY, Chao EK, Tu YK, Ueng SW, Shih CH. Closed management and percutaneous fixation of unstable proximal humerus fractures. J Trauma Injury Infect Crit Care. 1998;45(6):1039.
158. Chen Q, An ZQ, Zeng BF. Progress in artificial humeral head replacement for complex proximal humerus fractures. CRTER. 2007;11(34):6841.
159. Chen YF, Zhu NF, Zhang CQ, Wang L, Wei HF, Lu Y. The relevance of the anatomical basis of fracture for the subsequent treatment of the anterior humeral circumflex artery and the axillary nerve. Int Orthop. 2012; 36(4):783.
160. Cheng B, Lin JP, Chen ZR, Yao ZJ, Zhang C. Therapy of comminuted fracture of proximal humerus with locking proximal humerus plate. Fudan University Journal of Medical Sciences. 2005; 32(1):92.
161. Cheung EV. Locked plating for proximal humeral fractures. Curr Orthop Pract. 2008;19(5): 535.
162. Cheung S, Fitzpatrick M, Lee TQ. Effects of shoulder position on axillary nerve positions during the split lateral deltoid approach. J Shoulder Elbow Surg. 2009;18(5):748.
163. Chiavola M, Ciclamini D, Zoccola K, Masse A. Treatment of proximal humerus fractures with shoulder prosthesis. Minerva Ortopedica e Traumatologica. 2003;54(1):13.
164. Cho CH, Jung GH, Song KS. Tension suture fixation using 2 washers for proximal humeral fractures. Orthopedics. 2012;35(3):202.
165. Chow M, Begum F, Beaupre LA, Carey JP, Adeeb S, Bouliane MJ. Proximal humeral fracture fixation: locking plate construct +/- intramedullary fibular allograft. J Shoulder Elbow Surg. 2012;21(7):894.
166. Chu SP, Kelsey JL, Keegan TH, Sternfeld B, Prill M, Quesenberry CP,Sidney s. Risk factors for proximal humerus fracture. Am J Epidemiol.2004;160(4):360.
167. Chudik SC, Weinhold P, Dahners LE. Fixed-angle plate fixation in simulated fractures of the proximal humerus: a biomechanical study of a new device. J Shoulder Elbow Surg. 2003;12(6):578.
168. Chun JM, Groh GI, Rockwood CA. Two-part fractures of the proximal humerus. J Shoulder Elbow Surg. 1994;3(5):273.
169. Cikes A, Winter M, Trojani C, Boileau P. Hemiarthroplasty for displaced proximal humerus fractures, preliminary results of two types of implants. Swiss Med Wkly. 2009;139(23-24):14S.
170. Philippe Clavert P, Adam P, Bevort A, Bonnomet F, Kempf JF. Pitfalls and complications with locking plate for proximal humerus fracture. J Shoulder Elbow Surg. 2010;19(4):489.
171. Clifford PC. Fractures of the neck of the humerus: a review of the late results. Injury. 1980;12(2):91.
172. Coleman A, Clifft J. The effect of shoulder immobilization on balance in community-dwelling older adults. J Geriatr Phys Ther. 2010;33(3):118.
173. Coleman SH, Craig EV. Hemiarthroplasty for complex fractures of the proximal humerus: surgical technique and results with the Atlas trimodular prosthesis. Am J Orthop Surg. 2002;31(1 Suppl):11.
174. Colombini A, Lombardi G, Galliera E, Dogliotti G, Randelli P, Meerssemann A, Mineo G, Cabitza P, Corsi MM. Plasma and drainage fluid levels of soluble receptor activator of nuclear factor-kB (sRANK), soluble receptor activator of nuclear factor-kB ligand (sRANKL) and osteoprotegerin (OPG) during proximal humerus fracture healing. Int Orthop. 2011;35(5):777.
175. Compito CA, Self EB, Bigliani LU. Arthroplasty and acute shoulder trauma. Reasons for success and failure. Clin Orthop Relat Res. 1994;307:27.
176. Connor PM, D'Alessandro DF. Role of hemiarthroplasty for proximal humeral fractures. J South Orthop Assoc. 1995;4(1):9.
177. Connor PM, Flatow EL. Complications of internal fixation of proximal humeral fractures. Instr Course Lect. 1997;46:25.
178. Copuroglu C, Gurbuz H, Eskin D. Treatment of proximal humerus fractures with external fixation. Trak Univ Tip. Fak Derg. 2008;25(1):1.
179. Cornell CN, Ayalon O. Evidence for success with locking plates for fragility fractures. HSS Journal. 2011;7(2):164.
180. Cornell CN. Tension-band wiring supplemented by lag-screw fixation of proximal humerus fractures: a modified technique. Orthop Rev.1994; Suppl:19.
181. Cornell CN, Levine D, Pagnani MJ. Internal fixation of proximal humerus fractures using the screw-tension band technique. J Orthop Trauma. 1994;8(1):23.
182. Court-Brown CM, Cattermole H, McQueen MM. Impacted valgus fractures (B1.1) of the proximal humerus. The results of non-operative treatment. JBJS (Br). 2002;84(4):504.
183. Court-Brown CM, Garg A, McQueen MM. The translated two-part fracture of the proximal humerus. Epidemiology and outcome in the older patient. JBJS (Br). 2001;83(6):799.
184. Court-Brown CM, Garg A, McQueen MM. The epidemiology of proximal humeral fractures. Acta Orthop Scand. 2001;72(4):365.
185. Court-Brown CM, McQueen MM. The impacted varus (A2.2) proximal humeral fracture: prediction of outcome and results of nonoperative treatment in 99 patients. Acta Orthop Scand. 2004;75(6):736.
186. Court-Brown CM, McQueen MM. The relationship between fractures and increasing age with reference to the proximal humerus. Curr Orthop. 2002;16(3):213.
187. Craig EV. Prosthetic replacement for four-part fractures of the proximal humerus. Tech Orthop. 1989;3(4):70.
188. Crosby LA, Finnan RP, Anderson CG, Gozdanovic J, Miller MW. Tetracycline labeling as a measure of humeral head viability after 3-or 4-part proximal humerus fracture. J Shoulder Elbow Surg. 2009;18(6):851.
189. Cuenca J, Garcia-Erce JA, Munoz M, Martinez AA, Peguero A, Herrera A. Implementation of a restrictive transfusion protocol reduces the need for allogeneic blood transfusion in proximal humerus fracture repair without affecting patient outcome: Preliminary observations. Transfus Altern Transfus Med. 2008;10(1):17.
190. Cuny C, Darbelley L, Touchard O, Irrazi M, Beau P, Berrichi A, Empereur F. Proximal 4-part humerus fractures treated by antegrade nailing with self-stabilizing screws: 31 cases. Revue de Chirurgie Orthopedique et Reparatrice de l Appareil Moteur. 2003;89(6):507.
191. Cuny C, Goetzmann T, Irrazi MB, Berrichi A, Dedome D, Mainard D. Development of the telegraph nail for proximal humeral fractures. Injury Conference. 2012;43:S5.
192. Cuny C, Pfeffer F, Irrazi M, Chammas M, Empereur F, Berrichi A, Metais P, Beau P. A new locking nail for proximal humerus fractures: the Telegraph nail, technique and preliminary results. Revue de Chirurgie Orthopedique et Reparatrice de l Appareil Moteur. 2002;88(1):62.
193. Cuny C, Scarlat MM, Irrazi M, Beau P, Wenger V, Ionescu N, Berrichi A. The Telegraph nail for proximal humeral fractures: a prospective four-year study. J Shoulder Elbow Surg. 2008;17(4):539.
194. Cuny C, Irrazi M, Neyton L, Berrichi A, Beau P. Telegraph nail used for internal fixation of proximal humerus fracture. Eur J Orthop Surg Traumatol. 2000;10(1):21.
195. Cuomo F, Flatow EL, Maday MG, Miller SR, McIlveen SJ, Bigliani LU. Open reduction and internal fixation of two- and three-part displaced surgical neck fractures of the proximal humerus. J Shoulder Elbow Surg. 1992;1(6):287.
196. Cuomo F, Zuckerman JD. Open reduction and internal fixation of two- and three- part proximal humerus fractures. Tech Othrop. 1994;9(2):141.
197. Dahners LE. Internal fixation of proximal humeral fractures. J South Orthop Assoc. 1995;4(1):3.
198. Dall'Oca C, Maluta T, Bartolozzi P. Cement augumentation method for proximal epiphyseal humeral fractures in osteoporothic elderly patients treated by intramedullary nailing and plates and screws: A 1-year follow-up. J Orthop Trauma. 2011;12:S10.
199. Darder A, Darder A, Sanchis V, Gastaldi E, Gomar F. Four-part displaced proximal humeral fractures: operative treatment using Kirschner wires and a tension band. J Orthop Trauma. 1993;7(6):497.
200. Dawson FA. Four-part fracture dislocation of the proximal humerus: an arthroscopic approach. Arthroscopy. 2003;19(6):662.
201. De Andrade RP, Pires PR, Pereira JAR. Blocked flexible nails: A new method for the surgical treatment of proximal humerus fractures. Revista Brasileira de Ortopedia. 1998;33(9):695.
202. De la Hoz Marin J, Cortes PH, Sanchez JT. Surgical treatment of three-part proximal humeral fractures. Acta Orthop Belg. 2011; 67(3):226.
203. De Tullio V, Orsi R, Barisone P, Celenza M. Long term follow-up of surgical treatment of proximal humeral fractures. Minerva Ortopedica e Traumatologica. 1994; 45(10):437.
204. De Wilde LF, Berghs BM, Beutler T, Ferguson SJ, Verdonk RC. A new prosthetic design for proximal humeral fractures: Reconstructing the glenohumeral unit. J Shoulder Elbow Surg. 2004;3(4):373.
205. DeLuise A, Sadeghi C, Bhandari M, Tornetta P. Correlation of angulation on initial and healed radiographic views of surgical humerus neck fractures Orthopedics. 2008;31(6):548.
206. Demirhan M, Kilicoglu O, Altinel L, Eralp L, Akalin Y. Prognostic factors in prosthetic replacement for acute proximal humerus fractures. J Orthop Trauma. 2003;17(3): 181.
207. den Hartog D, de Haan J, Schep NW, Tuinebreijer WE. Primary shoulder arthroplasty versus conservative treatment for comminuted proximal humeral fractures: a systematic literature review.Open Orthop J. 2010;4:87.
208. Den Hartog D, Van Lieshout EM, Tuinebreijer WE, Polinder S, Van Beeck EF, Breederveld RS, Bronkhorst MW, Eerenberg JP, Rhemrev S, Roerdink WH, Schraa G, Van der Vis HM, Van Thiel TP, Patka P, Nijs S, Schep NW. Primary hemiarthroplasty versus conservative treatment for comminuted fractures of the proximal humerus in the elderly (ProCon): a multicenter randomized controlled trial. BMC Musculoskelet Disord. 2010;11:97.
209. Des Marchais JE, Benazet JP. Evaluation of Neer's hemi-arthroplasty in the treatment of humeral fractures. Can J Surg. 1983;26(5):469.
210. Di Ieso T, Maiello V, Fausto S, Quaranta M, Levantesi MT, Gentili C, Grippaudo N, Cavaciocchi E, Tenze G, Gagliardi S, Ciacca T. Locoregional anesthesia in elective humerus and shoulder surgery: The role of associated general anesthesia. Region Anesth Pain Med (Conf.). 2012;37(5 SUPPL. 1):E220.
211. Dietrich M, Meier C, Lattmann T, Zingg U, Gruninger P, Platz A. Complex fracture of the proximal humerus in the elderly. Locking plate osteosynthesis vs hemiarthroplasty. Chirurg. 2008;79(3):231.
212. Dietrich M, Meier C, Zeller D, Grueninger P, Berbig R, Platz A. Primary hemiarthroplasty for proximal humeral fractures in the elderly: Long-term functional outcome and social implications. Eur J Trauma Emerg Surg. 2007;33(5):512.
213. Dietz O, Broos P, Nijs S. Suture fixation versus cable cerclage of the tuberosities in shoulder arthroplasty-clinical and radiologic results. Arch Orthop Trauma Surg. 2012;132(6):793.
214. Dietz O, Hartmann F, Schwarz T, Nowak TE, Enders A, Kuhn S, Hofmann A, Rommens PM. Retrograde nailing versus locking plate osteosynthesis of proximal humeral fractures: a biomechanical study. J Shoulder Elbow Surg. 2012;21(5):618.
215. Diklic ID, Ganic ZD, Stevanovic VB, Crnobaric AS, Glisic M, Blagojevic ZB. Fixation of the tuberculum in multifragment fractures of the proximal humerus treated with hemiarthroplasty. Acta Chir Iugosl. 2010;57(1):31.
216. Dimakopoulos P, Panagopoulos A, Kasimatis G. Transosseous suture fixation of proximal humeral fractures. Surgical technique. JBJS. 2009;91(Suppl 2 Pt 1):8.
217. Dimakopoulos P, Potamitis N, Lambiris E. Hemiarthroplasty in the treatment of comminuted intraarticular fractures of the proximal humerus. Clin Orthop Relat Res. 1997;341:7.
218. Dimakopoulos P, Panagopoulos A, Kasimatis G. Transosseous suture fixation of proximal humeral fractures. JBJS. 2007;89A(8):1700.
219. Dines DM, Moynihan DP. Reverse Shoulder Replacement: Forward Thinking in Acute Fractures-Opposes. Semin Arthroplasty. 2008;19(1):5.
220. Dines DM, Warren RF. Modular shoulder hemiarthroplasty for acute fractures. Surgical considerations. Clin Orthop Relat Res. 1994;307:18.
221. Dines DM, Warren RF, Craig EV, Lee D, Dines JS. Intramedullary fracture positioning sleeve for proper placement of hemiarthroplasty in fractures of the proximal humerus. Tech Should Elb Surg . 2007;8(2):69.
222. Ding SL, Guo ZD, Wang ZL. Percutaneous pinning for the treatment of proximal fractures of humerus. Zhongguo Gushang. 2009; 22(5):363.
223. Dobele S, Eichhorn S, Lenich A, Trapp OM, Schreiber U, Stockle U. Morphological analysis of a novel poly-axial intramedullary nailing concept for the treatment of proximal humerus fractures. Injury (Conf). 2012;43:S5.
224. Doetsch AM, Faber J, Lynnerup N, Watjen I, Bliddal H, Danneskiold-Samsoe B. The effect of calcium and vitamin D-3 supplementation on the healing of the proximal humerus fracture: A randomized placebo-controlled study. Calcif Tissue Int. 2004;75(3):183.
225. Doursounian L. Re: "Comparative experimental study of 3 systems of osteosynthesis for fractures of the proximal humerus”. Rev Chir Orthop Reparatrice Appar Mot. 2000;86(4):414.
226. Doursounian L, Grimberg J, Cazeau C, Jos E, Touzard RC. A new internal fixation technique for fractures of the proximal humerus--the Bilboquet device: a report on 26 cases. J Shoulder Elbow Surg. 2000; 9(4):279.
227. Doursounian L, Grimberg J, Cazeau C, Touzard RC. A new method of osteosynthesis in proximal humeral fractures: a new internal fixation device. Apropos of 17 cases followed over more than 2 years. Revue de Chirurgie Orthopedique et Reparatrice de l Appareil Moteur. 1996;82(8):743.
228. Doursounian L, Kilinc A, Cherrier B, Nourissat G. Complex proximal humeral fractures: A prospective study of 22 cases treated using the "Bilboquet" device. Orthop Traumatol Surg Res. 2011;97(1):58.
229. Duda GN, Epari DR, Babst R, Lambert SM, Matthys R, Sudkamp NP. Mechanical evaluation of a new minimally invasive device for stabilization of proximal humeral fractures in elderly patients: a cadaver study. Acta Orthopaedica. 2007;78(3):430.
230. Dudko S, Wojciechowski P, Kusz D, Wrobel W. Inferior subluxation of humerus after surgery of fractures of proximal humerus. Chir Narzadow Ruchu Ortop Pol. 2002;67(2):175.
231. Duralde XA, Leddy LR. The results of ORIF of displaced unstable proximal humeral fractures using a locking plate. J Shoulder Elbow Surg. 2010;19(4):480.
232. Durigan A, Barbieri CH, Mazzer N, Shimano AC. Two-part surgical neck fractures of the humerus: mechanical analysis of the fixation with four Shanz-type threaded pins in four different assemblies. J Shoulder Elbow Surg. 2005;14(1):96.
233. Ebraheim N, Wong FY, Biyani A. Percutaneous pinning of the proximal humerus. Am J Orthop. 1996;25(7):500.
234. Ebraheim NA, Patil V, Husain A. Mini-external fixation of two- and three-part proximal humerus fractures. Acta Orthop Belg. 2007;73(4):437.
235. Edelmann K, Obruba P, Kopp L, Cihlar J, Celko AM. Comparison of functional outcomes in angle-stable osteosynthesis of comminuted fractures of the proximal humerus with those in percutaneous kirschner-wire fixation. a prospective study of mid-term results. Acta Chir Orthop Traumatol Cech. 2011;78(4):314.
236. Edelson G, Kelly I, Vigder F, Reis ND. A three-dimensional classification for fractures of the proximal humerus. JBJS (Br). 2004;86(3):413.
237. Edelson G, Safuri H, Salami J, Vigder F, Militianu D. Natural history of complex fractures of the proximal humerus using a three-dimensional classification system. J Shoulder Elbow Surg. 2008;17(3):399.
238. Gordon Edelson G, Saffuri H, Obid E, Vigder F. The three-dimensional anatomy of proximal humeral fractures. J Shoulder Elbow Surg. 2009;18(4):535.
239. Edwards SL, Wilson NA, Zhang LQ, Flores S, Merk BR. Two-part surgical neck fractures of the proximal part of the humerus. A biomechanical evaluation of two fixation techniques. JBJS. 2006;88(10):2258.
240. Egol KA, Ong CC, Walsh M, Jazrawi LM, Tejwani NC, Zuckerman JD. Early complications in proximal humerus fractures (OTA Types 11) treated with locked plates. J Orthop Trauma. 2008;22(3):159.
241. Egol KA, Sugi MT, Ong CC, Montero N, Davidovitch R, Zuckerman JD. Fracture site augmentation with calcium phosphate cement reduces screw penetration after open reduction-internal fixation of proximal humeral fractures. J Shoulder Elbow Surg. 2012;21(6):741.
242. Ehlinger M, Gicquel P, Clavert P, Bonnomet F, Kempf JF. A new implant for proximal humeral fracture: experimental study of the basket plate. Rev Chir Orthop Reparatrice Appareil Moteur. 2004;90(1):16.
243. Eichhorn S, Grandl M, Trapp OM, Schreiber U. The PoliAx-Nail; evaluation of a novel poly-axial and angle-stable intramedullary nailing concept. Injury (Conf). 2011;42:S21.
244. Eid A, Osman M, Fekry HE. Percutaneous fixation with Schanz screws for displaced two- and three- part fractures of the proximal humerus in patients above fifty years of age. Int J Shoulder Surg. 2011;5(2):38.
245. Einsiedel T, Becker C, Stengel D, Schmelz A, Kramer M, Daexle M, Lechner F, Kinzl L, Gebhard F. Do injuries of the upper extremity in geriatric patients end up in helplessness? A prospective study for the outcome of distal radius and proximal humerus fractures in individuals over 65. Z Gerontol Geriatr. 2006;39(6):451.
246. El-Alfy BS. Results of the percutaneous pinning of proximal humerus fractures with a modified palm tree technique. Int Orthop. 2011;35(9):1343.
247. El-Sayed MM. Surgical management of complex humerus head fractures. Orthop Rev. 2010;2(2):e14.
248. Erhardt JB, Stoffel K, Kampshoff J, Badur N, Yates P, Kuster MS. The position and number of screws influence screw perforation of the humeral head in modern locking plates: a cadaver study. J Orthop Trauma. 2012;26(10):e188.
249. Erhardt JB, Roderer G, Grob K, Forster TN, Stoffel K, Kuster MS. Early results in the treatment of proximal humeral fractures with a polyaxial locking plate. Arch Orthop Trauma Surg. 2009;129(10):1367.
250. Erhart J, Heinz T, Schuster R, Greitbauer M, Vecsei V. Stabilization of proximal humeral fractures with the intramedullary dynamic titanium helix wire. Wien Klin Wochenschr. 2002;114(19-20):859.
251. Esen E, Dogramaci Y, Gultekin S, Deveci MA, Suluova F, Kanatli U, Bolukbasi S. Factors affecting results of patients with humeral proximal end fractures undergoing primary hemiarthroplasty: a retrospective study in 42 patients. Injury. 2009;40(12):1336.
252. Esen E, Dogramaci Y, Komurcu M, Kanatli U, Bolukbasi S, Atahan AO. Biomechanical comparison of fixation of two-part osteoporotic neck fracture of the proximal humerus using uni-planar and multi-planar Kirschner wire. Eklem hastaliklari ve cerrahisi. 2009;20(2):114.
253. Esser RD. Treatment of three- and four-part fractures of the proximal humerus with a modified cloverleaf plate. J Orthop Trauma. 1994;8(1):15.
254. Esser RD. Open reduction and internal fixation of three- and four-part fractures of the proximal humerus. Clin Orthop Relat Res. 1994;299:244.
255. Falborg B, Palm H, Fenger AM, Anderson K, Jensen CH. Outcome of cemented Neer II hemiarthroplasty in displaced humeral head fractures. Acta Orthop Belg. 2008;74(1):7.
256. Falis M, Czarnecki P, Romanowski L, Wcislek J. Displaced proximal humerus fractures; comparison non-operative vs. operative treatment using intramedullary nailing targon PH. Injury (Conf). 2011;42:S2.
257. Fallatah S, Dervin GF, Brunet JA, Conway AF, Hrushowy H. Functional outcome after proximal humeral fractures treated with hemiarthroplasty. Can J Surg. 2008;51(5):361.
258. Fan Y, Wang S, Luo Y. Effectiveness comparison of operative and non-operative treatment for complex proximal humeral fractures in elderly patients. Chung-Kuo Hsiu Fu Chung Chien Wai Ko Tsa Chih. 2012;26(9):1029.
259. Fan Z, Zhou L, Cao Z. Nursing care of patients with proximal humerus fracture treated with micro-invasive locking internal fixation [Chinese]. Chinese Nurs Res. 2009;23(7):1917.
260. Fankhauser F, Boldin C, Schippinger G, Haunschmid C, Szyszkowitz R. A new locking plate for unstable fractures of the proximal humerus. Clin Orthop Relat Res. 2005;430:176.
261. Fankhauser F, Schippinger G, Weber K, Heinz S, Quehenberger F, Boldin C, Bratschitsch G, Szyszkowitz R, Georg L, Friedrich A. Cadaveric-biomechanical evaluation of bone-implant construct of proximal humerus fractures (Neer type 3). J Trauma-Injury Infect Crit Care. 2003;55(2):345.
262. Fankhauser F, Weber K, Schippinger G, Sablatnog H, Boldin CH, Bratschitsch G, Leb G, Szyszkowitz R. The influence of bone density on the genesis of fractures and the stability after osteosynthesis of proximal humerus fractures. Journal fur Mineralstoffwechsel. 2002;9(1):28.
263. Faraj D, Kooistra BW, v.d. Stappen WAH, Werre AJ. Results of 131 consecutive operated patients with a displaced proximal humerus fracture: an analysis with more than two years follow-up. Eur J Orthop Surg Traumatol. 2011;21(1):7.
264. Farmer KW, Wright TW. Three- and Four-Part Proximal Humerus Fractures: Open Reduction and Internal Fixation Versus Arthroplasty. J Hand Surg Am Vol. 2010;35A(11):1881.
265. Farng E, Zingmond D, Krenek L, Soohoo NF. Factors predicting complication rates after primary shoulder arthroplasty. J Shoulder Elbow Surg. 2011;20(4):557.
266. Fassbender M, Rothenbacher G, Weschler C, Muller CA, Pfister U. Minimally invasive T-plating at the proximal humerus. Zeitschrift fur Orthopadie und Unfallchirurgie. 2008;146(3):318.
267. Fat DL, Kennedy J, Galvin R, O'Brien F, Grath FM, Mullett H. The Hounsfield value for cortical bone geometry in the proximal humerus-an in vitro study. Skeletal Radiol. 2012;41(5):557.
268. Favard L, Vandeubussche E, Rabarin F, Schmider L. Epidemiology of proximal humeral fractures. Rev Chir Orthop Reparatrice Appar Mot. 1998;84(SUPPL. 1):136.
269. Fayad F, Lefevre-Colau MM, Mace Y, Gautheron V, Fermanian J, Roren A, Roby-Brami A, Revel M, Poiraudeau S. Responsiveness of the French version of the Disability of the Arm, Shoulder and Hand questionnaire (F-DASH) in patients with orthopaedic and medical shoulder disorders. Joint, Bone, Spine: Revue du Rhumatisme. 2008;75(5):579.
270. Fazal MA, Haddad FS. Philos plate fixation for displaced proximal humeral fractures. J Orthop Surg. 2009;17(1):15.
271. Fedorov IN. Surgical treatment of fractures in the proximal part of the humerus. Khirurgiia. 1968;44(10):35.
272. Fenichel I, Oran A, Burstein G, Pritsch MP. Percutaneous pinning using threaded pins as a treatment option for unstable two- and three-part fractures of the proximal humerus: a retrospective study. Int Orthop. 2006;30(3):153.
273. Neto AAF, Filho AAF, Filho AZ, Benegas E, Negri JH, de Castro Machado LFM, Oliveira FR. Internal fixation of two and three-part proximal humerus fractures with modified Enders rod associated to suture bands. Revista Brasileira de Ortopedia. 1977; 32(9):707.
274. Fialka C, Stampfl P, Arbes S, Reuter P, Oberleitner G, Vecse V. Primary hemiarthroplasty in four-part fractures of the proximal humerus: randomized trial of two different implant systems. J Shoulder Elbow Surg. 2008;17(2):210.
275. Gallotta GF, Zottola V, Bonacina P. Kerboull's plate in treatment of fractures of the proximal one-third of the humerus. Minerva Ortop. 1985;36(11):857.
276. Fjalestad T, Hole MO, Blucher J, Hovden IA, Stiris MG, Stromsoe K. Rotator cuff tears in proximal humeral fractures: an MRI cohort study in 76 patients. Arch Orthop Trauma Surg. 2010;130(5):575.
277. Fjalestad T, Hole MO, Jorgensen JJ, Stromsoe K, Kristiansen IS. Health and cost consequences of surgical versus conservative treatment for a comminuted proximal humeral fracture in elderly patients. Injury Int J Care Inj. 2010;41(6):599.
278. Fjalestad T, Stromsoe K, Blucher J, Tennoe B. Fractures in the proximal humerus: functional outcome and evaluation of 70 patients treated in hospital. Arch Orthop Trauma Surg. 2005;125(5):310.
279. Fjalestad T, Falch JA, Stromsoe K. Proximal humeral fractures and relation to osteoporosis: a cohort study of 49 hospitalized patients with 50 fractures. Eur J Orthop Surg Traumatol. 2009;19(7):461.
280. Fjalestad T, Hole MO, Hovden IAH, Blucher J. Surgical Treatment With an Angular Stable Plate for Complex Displaced Proximal Humeral Fractures in Elderly Patients: A Randomized Controlled Trial. J Orthop Trauma. 2012;26(2):98.
281. Flach K. Results of treatment of fractures of the proximal end of the humerus by Poelchen's method. Monatsschr Unfallheilkd Versicher Versorg Verkehrsmed. 1969;72(3):124.
282. Flatow EL. Technique of prosthetic replacement for proximal humeral fractures. Tech Orthop. 1994;9(2):154.
283. Flatow EL, Cuomo F, Maday MG, Miller SR, McIlveen SJ, Bigliani LU. Open reduction and internal fixation of two-part displaced fractures of the greater tuberosity of the proximal part of the humerus. JBJS. 1991;73(8):1213.
284. Flatow EL, Klug RA. Humeral Head Replacement: When Half a Loaf Will Do. Semin Arthroplasty. 2007;18(1):42.
285. Fleischer GM, Frohlich P. The operative treatment of the subcapital fracture of the humerus. Beitr Orthop Traumatol. 1981;28(3):147.
286. Fleischer J, Schleyer A, Nassutt R, Grundei H, Grittner U, Hopp SJ. Biomechanical investigation on refixation of tuberosities on shoulder prostheses. Does refixation with different suture materials offer enough stability? Unfallchirurg. 2010; 113(8):641.
287. Fleischmann W, Kinzl L. Philosophy of osteosynthesis in shoulder fractures. Orthopedics. 1993;16(1):59.
288. Florek J, Bednarenko M, Plezia B, Kotela I.Outcomes of surgical treatment of proximal humerus fractures in elderly people. Przeglad lekarski. 2010;67(5):376.
289. Foroohar A, Tosti R, Richmond JM, Gaughan JP, Ilyas AM. Classification and treatment of proximal humerus fractures: inter-observer reliability and agreement across imaging modalities and experience. J Orthop Surg Res. 2011;6:38.
290. Foruria AM, Carrascal MT, Revilla C, Munuera L, Sanchez-Sotelo J. Proximal humerus fracture rotational stability after fixation using a locking plate or a fixed-angle locked nail: the role of implant stiffness. Clin Biomech. 2010;25(4):307.
291. Foruria AM, de Gracia MM, Larson DR, Munuera L, Sanchez-Sotelo J. The pattern of the fracture and displacement of the fragments predict the outcome in proximal humeral fractures. JBJS (Br). 2011;93B(3):378.
292. Frangen TM, Dudda M, Martin D, Arens S, Greif S, Muhr G, Kalicke T. Proximal humeral fractures with angle-stable plate osteosynthesis - Is everything better now? Zentralbl Chir. 2007;132(1):60.
293. Frangen TM, Muller EJ, Dudda M, Arens S, Muhr G, Kalicke T. Proximal humeral fractures in geriatric patients. Is the angle-stable plate osteosynthesis really a breakthrough? Acta Orthop Belg. 2007;73(5):571.
294. Frankle MA, Greenwald DP, Markee BA, Ondrovic LE, Lee WE. Biomechanical effects of malposition of tuberosity fragments on the humeral prosthetic reconstruction for four-part proximal humerus fractures. J Shoulder Elbow Surg. 2011;10(4):321.
295. Frankle MA, Ondrovic LE, Markee BA, Harris ML, Lee WE. Stability of tuberosity reattachment in proximal humeral hemiarthroplasty. J Shoulder Elbow Surg. 2002;11(5):413.
296. Frankle MA, Mighell MA. Techniques and principles of tuberosity fixation for proximal humeral fractures treated with hemiarthroplasty. J Shoulder Elbow Surg. 2004;13(2): 239.
297. Fric V, Pazdirek P, Bartonicek J. Unreamed locking intramedullary nailing of humeral fractures--basic evaluation of a patient group. Acta Chir Orthop Traumatol Cech. 2001;68(6):345.
298. Fric V, Sosna A. Contribution to the problem of classification of fractures of the proximal end of the humerus in adults. Acta Chir Orthop Traumatol Cech. 1995;62(4):196.
299. Frich LH, Sojbjerg JO, Sneppen O. Shoulder arthroplasty in complex acute and chronic proximal humeral fractures. Orthopedics. 1991;14(9):949.
300. Friedl W, Rinner M. Experimental examination of factors influencing the deformation cut out risk in locked nail osteosynthesis of proximal humerus fractures. Injury (Conf). 2012;43:S11.
301. Friess DM, Attia A, Vallier HA. Locking plate fixation for proximal humerus fractures: A comparison with other fixation techniques. Orthopedics. 2008;31(12):1183.
302. Fu ZG, Deng L, Bai L, Chen JH, Zhang PX, Dang Y, Zhang DY, Jiang BG. Complication analysis of proximal humeral fractures treated with locking plate. Beijing da Xue Xue Bao. 2011;43(5):666.
303. Fuchs M, Burchhardt H, Losch A, Sturmer KM. Open reduction and internal fixation of proximal humerus fractures with a cannulated blade plate. Orthop Traumatol. 2002;10(4):268.
304. Fuchs M, Losch A, Sturmer KM. The cannulated blade plate 90 degrees for displaced proximal humeral fractures in elderly patients. Zentralbl Chir. 2003;128(1):22.
305. Fuchtmeier B, Brockner S, Hente R, Maghsudi M, Nerlich M, Prantl L. The treatment of dislocated humeral head fractures with a new proximal intramedullary nail system. Int Orthop. 2008;32(6):759.
306. Fuchtmeier B, May R, Fierlbeck J, Hammer J, Nerlich M. A comparative biomechanical analysis of implants for the stabilization of proximal humerus fractures. Technol Health Care. 2006;14(4-5):261.
307. Funakoshi T, Tago H, Suzuki K, Kurobe Y, Yosida M, Tandai S. Surgical treatment of proximal humeral fractures at Kusiro City General Hospital. Hokkaido J Orthop Traumatol. 2000;42(1):1.
308. Furnstahl P, Szekely G, Gerber C, Hodler J, Snedeker JG, Harders M. Computer assisted reconstruction of complex proximal humerus fractures for preoperative planning. Med Image Anal. 2012;16(3):704.
309. Gaebler C, Mcqueen MM, Court-Brown CM. Minimally displaced proximal humeral fractures. Acta Orthop Scand. 2003;74(5):580.
310. Gaheer RS, Hawkins A. Fixation of 3- and 4-part proximal humerus fractures using the PHILOS plate: mid-term results. Orthopedics 2010;33(9):671.
311. Gahr RH, Imhoff M, Sadr I, Tassler H. Therapy of humerus fractures of the collum chirurgicum. Zeitschrift fur Unfallchirurgie und Versicherungsmedizin. 1990;83(4):206.
312. Galatz LM. Percutaneous Pinning for Surgical Neck Fracture: Method of Choice-Affirms. Semin Arthroplasty. 2007;18(1):19.
313. Gallinet D, Clappaz P, Garbuio P, Tropet Y, Obert L. Three or four parts complex proximal humerus fractures: Hemiarthroplasty versus reverse prosthesis: A comparative study of 40 cases. Orthop Traumatol Surg Res. 2009;95(1):48.
314. Gallo RA, Altman DT, Altman GT. Assessment of rotator cuff tendons after proximal humerus fractures: is preoperative imaging necessary? J Trauma Injury Infect Crit Care. 2009;66(3):951.
315. Gallo RA, Hughes T, Altman G. Tips & techniques. Percutaneous plate fixation of two- and three-part proximal humerus fractures Orthopedics. 2008;31(3):237.
316. Gallo RA, Sciulli R, Daffner RH, Altman DT, Altman GT. Defining the relationship between rotator cuff injury and proximal humerus fractures. Clin Orthop Relat Res. 2007;458:70.
317. Gallo RA, Zeiders GJ, Altman GT. Technical tricks. Two-incision technique for treatment of complex proximal humerus fractures. J Orthop Trauma. 2005;19(10):734.
318. Galvano N, Parlato A, D'Arienzo A, D'Arienzo M. Indications and limits of the Fixator TGF "gex-Fix" in fractures of the proximal end of the humerus. J Orthop Trauma (Conf.) 2011;12:S6.
319. Gardner MJ, Boraiah S, Helfet DL, Lorich DG. The anterolateral acromial approach for fractures of the proximal humerus. J Orthop Trauma. 2008; 22(2):132.
320. Gardner MJ, Boraiah S, Helfet DL, Lorich DG. Indirect medial reduction and strut support of proximal humerus fractures using an endosteal implant. J Orthop Trauma. 2008;22(3):195.
321. Gardner MJ, Griffith MH, Dines JS, Briggs SM, Weiland AJ, Lorich DG. The extended anterolateral acromial approach allows minimally invasive access to the proximal humerus. Clin Orthop Relat Res. 2005;434:123.
322. Gardner MJ, Griffith MH, Dines JS, Lorich DG. A minimally invasive approach for plate fixation of the proximal humerus. Bull Hosp Joint Dis. 2004;62(1-2):18.
323. Gardner MJ, Griffith MH, Lorich DG. Helical plating of the proximal humerus. Injury. 2005;36(10):1197.
324. Gardner MJ, Weil Y, Barker JU, Kelly BT, Helfet DL, Lorich DG. The importance of medial support in locked plating of proximal humerus fractures. J Orthop Trauma. 2007;21(3):185.
325. Gardner MJ, Voos JE, Wanich T, Helfet DL, Lorich DG. Vascular implications of minimally invasive plating of proximal humerus fractures. J Orthop Trauma. 2006;20(9):602.
326. Garnavos C. Humeral nails: when to choose what and how to use. Curr Orthop. 2005;19(4):294.
327. Garnavos C, Lasanianos N. Intramedullary nailing of combined/extended fractures of the humeral head and shaft. J Orthop Trauma. 2010;24(4):199.
328. Garrigues GE, Johnston PS, Pepe MD, Tucker BS, Ramsey ML, Austin LS. Hemiarthroplasty Versus Reverse Total Shoulder Arthroplasty for Acute Proximal Humerus Fractures in Elderly Patients Orthopedics. 2012;35(5):E703.
329. Gaumet G, Boniface O, Wavreille G, Leroy M, Vervoort T, Chantelot C. Proximal humerus fractures using T2 nailing. A review of 38 cases. Chir Main. 2010;29(2):58.
330. Gautier E, Slongo T, Jakob RP. Treatment of subcapital humerus fracture with the Prevot nail. Zeitschrift fur Unfallchirurgie und Versicherungsmedizin. 1992;85(3):145.
331. Gavaskar AS, Muthukumar S, Chowdary N. Biological osteosynthesis of complex proximal humerus fractures: surgical technique and results from a prospective single center trial. Arch Orthop Trauma Surg. 2010;130(5):667.
332. Geiger EV, Maier M, Kelm A, Wutzler S, Seebach C, Marzi I. Functional outcome and complications following PHILOS plate fixation in proximal humeral fractures. Acta Orthop Traumatol Turc. 2010;44(1):1.
333. Georgousis M, Kontogeorgakos V, Kourkouvelas S, Badras S, Georgaklis V, Badras L. Internal fixation of proximal humerus fractures with the Polarus intramedullary nail. Acta Orthop Belg. 2010;76(4):462.
334. Gerber C, Werner CM, Vienne P. Internal fixation of complex fractures of the proximal humerus. JBJS (Br). 2004;86(6):848.
335. Gicquel P, Bonnomet F, Boutemy P, Schlemmer B, Kempf JF. Experimental comparative study of 3 systems of osteosynthesis for proximal humeral fractures. Preliminary study of the mechanical properties of conserved trabecular bone. Rev Chir Orthop Reparatrice Appar Mot. 1999;85(8):811.
336. Gierer P, Simon C, Gradl G, Ewert A, Vasarhelyi A, Beck M, Mittlmeier T. Complex proximal humerus fractures - Management with a humeral head prosthesis? Clinical and radiological results of a prospective study. Orthopade. 2006;35(8):834.
337. Gierer P, Scholz M, Beck M, Schaser KD, Vollmar B, Mittlmeier T, Gradl G. Microcirculatory sequelae of the rotator cuff after antegrade nailing in proximal humerus fracture. Arch Orthop Trauma Surg. 2010;130(5):687.
338. Gillespie RJ, Ramachandran V, Lea ES, Vallier HA. Biomechanical evaluation of 3-part proximal humerus fractures: a cadaveric study. Orthopedics. 2009;32(11):816.
339. Giovale M, Caione G, Dynamidis S, Franchin F. Prosthetic treatment of complex proximal humeral fracture in old patients: Anatomic versus inverted prosthesis. Minerva Ortopedica e Traumatologica. 2006;57(4):389.
340. Giuliani E, Ferrari G, Zuccarello L, Magni G, Montagnani G, Tassi A. Oral morphine vs intravenous midazolam premedication for repair of proximal humerus fractures - Prospective randomized trial preliminary results. Eur J Anaesthesiol. 2011;28:208.
341. Gobel F, Wuthe T, Reichel H. Results of shoulder hemiarthroplasty in patients with acute and old fractures of the proximal humerus. Z Orthop Ihre. 1999;137(1):25.
342. Godeneche A. Shoulder prosthesis for treatment of proximal humerus fractures. Revue du Rhumatisme Monographies. 2010;77(3):203.
343. Goldhahn S, Kralinger F, Rikli D, Marent M, Goldhahn J. Does osteoporosis increase complication risk in surgical fracture treatment? A protocol combining new endpoints for two prospective multicentre open cohort studies. BMC Musculoskelet Disord. 2010;11:256.
344. Goldman RT, Koval KJ, Cuomo F, Gallagher MA, Zuckerman JD. Functional outcome after humeral head replacement for acute three- and four-part proximal humeral fractures. J Shoulder Elbow Surg. 1995;4(2):81.
345. Golec E, Nowak S, Golec J, Jasiak-Tyrkalska B, Jurczak P. Proximal humerus fractures analysis of treatment and rehabilitation outcomes. Chir Narzadow Ruchu Ortop Pol. 2006;71(3):221.
346. Gorecki A, Struzik S. Alloplasty of the humeral joint. Ortopedia Traumatologia Rehabilitacja. 2003;5(1):24.
347. Gorschewsky O, Labler L, Krause F, Schweizer A. Minimal osteosynthesis of proximal humerus fractures with helix wire. Aktuelle Traumatol. 2002;32(1):8.
348. Gorschewsky O, Puetz A, Klakow A, Pitzl M, Neumann W. The treatment of proximal humeral fractures with intramedullary titanium helix wire by 97 patients. Arch Orthop Trauma Surg. 2005;125(10):670.
349. Gotzen L, Bahrs C, Leppek R, Schnabel M. Suggestion for a modular topographic-morphologic classification of proximal humeral fractures: Part 1: Presentation of the classification. Eur J Trauma. 2003;29(1):31.
350. Grabovoi AF, Rodichkin VA. Rehabilitation treatment of injuries of the shoulder joint. Vestnik khirurgii imeni I.I.Grekova. 1988; 141(10):65.
351. Gradl G, Dietze A, Kaab M, Hopfenmuller W, Mittlmeier T. Is locking nailing of humeral head fractures superior to locking plate fixation?. Clin Orthop Relat Res. 2009;467(11):2986.
352. Gradl G, Knobe M, Stoffel M, Prescher A, Dirrichs T, Pape HC. Biomechanical evaluation of locking plate fixation of proximal humeral fractures augmented with calcium phosphate cement. Injury (Conf). 2012;43:S11.
353. Gradl G, Pape HC. Upper extremity periarticular fractures: when should they be fixed and when should they be replaced? J Orthop Trauma. 2011;25(Suppl 2):S86.
354. Gradl G, Dietze A, Arndt D, Beck M, Gierer P, Boersch T, Mittlmeier T. Angular and sliding stable antegrade nailing (Targon PH) for the treatment of proximal humeral fractures. Arch Orthop Trauma Surg. 2007;127(10):937.
355. Granata F, Gandolfo N, Desayeux S. The use of the prosthesis of shoulder in the fractures of proximal epiphysis of the humerus: Our experience on 20 cases. Minerva Ortopedica e Traumatologica. 2006;57(3):87.
356. Grassi FA, Rolla P, Castelli C, Casali M, Giughello A, Cherubino P. Indications for surgical treatment of fractures of the proximal humerus. Minerva Ortopedica e Traumatologica. 1998;49(6):177.
357. Grassi FA, Tajana MS. Partial prosthetic replacement of the shoulder in fractures and fracture-dislocations of the proximal humerus. Chirurgia Degli Organi di Movimento. 2005;90(2):179.
358. Green A, Barnard WL, Limbird RS. Humeral head replacement for acute, four-part proximal humerus fractures. J Shoulder Elbow Surg. 1993;2(5):249.
359. Greiner S, Kaeaeb MJ, Haas NP, Bail HJ. Humeral head necrosis rate at mid-term follow-up after open reduction and angular stable plate fixation for proximal humeral fractures. Injury-Int J Care Inj. 2009;40(2):186.
360. Greiner SH, Diederichs G, Kroening I, Scheibel M, Perka C. Tuberosity position correlates with fatty infiltration of the rotator cuff after hemiarthroplasty for proximal humeral fractures. J Shoulder Elbow Surg. 2009;18(3):431.
361. Greiner SH, Kaeaeb MJ, Kroening I, Scheibel M, Perka C. Reconstruction of humeral length and centering of the prosthetic head in hemiarthroplasty for proximal humeral fractures. J Shoulder Elbow Surg. 2008;17(5):709.
362. Grisch D, Helmy N, Riede U. Inverse Total Shoulder Arthroplasty as primary treatment for complex proximal humerus fractures in elderly people. Swiss Med Wkly. 2010;140(23-24):26S.
363. Gronhagen CM, Abbaszadegan H, Revay SA, Adolphson PY. Medium-term results after primary hemiarthroplasty for comminute proximal humerus fractures: A study of 46 patients followed up for an average of 4.4 years. J Shoulder Elbow Surg. 2007;16(6):766.
364. Grossterlinden L, Ueblacker P, Rueger JM. Arthroscopical findings after antegrade nailing of a proximal humeral fracture. Eur J Trauma Emerg Surg. 2007;33(4):383.
365. Guity MR, Sibdari SY, Espandar R. Functional results after primary shoulder hemiarthroplasty for proximal humerus bone fractures. Tehran Univ Med J. 2010;68(4):231.
366. Guix JMM, Gonzalez AS, Brugalla JV, Carril EC. Proposed protocol for reding images of humeral head fractures. Clin Orthop Relat Res. 2006;448:225.
367. Gumina S, Giannicola G, Albino P, Passaretti D, Cinotti G, Postacchini F. Comparison between two classifications of humeral head fractures: Neer and AO-ASIF. Acta Orthop.Belg. 2011;77(6):751.
368. Gupta AK, Gupta M, Sengar G, Nath R. Functional outcome of closed fractures of proximal humerus managed by Joshi's external stabilizing system. Indian J Orthop. 2012;46(2):216.
369. Guy P, Slobogean GP, McCormack RG. Treatment preferences for displaced three- and four-part proximal humerus fractures. J Orthop Trauma. 2010;24(4): 250.
370. Haapamaki VV, Kiuru MJ, Koskinen SK. Multidetector CT in shoulder fractures. Indian J Orthop. 2004;11(2):89.
371. Habermeyer P, Ebert T. Current status and perspectives of shoulder replacement. Unfallchirurg. 1999;102(9):668.
372. Habermeyer P, Magosch P, Lichtenberg S. Proximal humeral head fractures - Future development of shoulderimplants. Zentralbl Chir. 2002;127(3):207.
373. Habernek H, Schneider R, Popp R, Weinstabl R, Schmid L, Barisani G, Mohr W. Spiral bundle nailing for subcapital humeral fractures: preliminary report on the method of Henning. J Trauma. 1999;46(3):400.
374. Halder SC, Chapman JA, Choudhury G, Wallace WA. Retrograde fixation of fractures of the neck and shaft of the humerus with the 'Halder humeral nail'. Injury. 2001;32(9):695.
375. Handoll H, Brealey S, Rangan A, Torgerson D, Dennis L, Armstrong A, Chuang LH, Cross B, Dumville J, Gardner S, Goodchild L, Hamilton S, Hewitt C, Madhok R, Maffulli N, Micklewright L, Wadsworth V, Wallace A, Williams J, Worthy G. Protocol for the ProFHER (PROximal Fracture of the Humerus: Evaluation by Randomisation) trial: a pragmatic multi-centre randomised controlled trial of surgical versus non-surgical treatment for proximal fracture of the humerus in adults. BMC Musculoskelet Disord. 2009;10:140.
376. Handschin AE, Cardell M, Contaldo C, Trentz O, Wanner GA. Functional results of angular-stable plate fixation in displaced proximal humeral fractures. Injury Int J Care Inj. 2008;39(3):306.
377. Hanson B, Neidenbach P, de Boer P, Stengel D. Functional outcomes after nonoperative management of fractures of the proximal humerus. J Shoulder Elbow Surg. 2009;18(4):612.
378. Hardeman F, Bollars P, Donnelly M, Bellemans J, Nijs S. Predictive factors for functional outcome and failure in angular stable osteosynthesis of the proximal humerus. Injury. 2012;43(2):153.
379. Harnroongroj T, Vanadurongwan V. The reconstruction twisted wire-screws for internal fixation of two- and three-part fractures of the proximal humerus. J Med Assoc Thai. 1998;81(4):250.
380. Harrison AK, Flatow EL. Tuberosity fracture management: Surgical macrame. Semin Arthroplasty. 2010;21(3):152.
381. Harrison AK, Gruson KI, Zmistowski B, Keener J, Galatz L, Williams G, Parsons BO, Flatow EL. Intermediate Outcomes Following Percutaneous Fixation of Proximal Humeral Fractures. J Bone Joint Surg Am Vol. 2012;94A(13):1223.
382. Hartsock LA, Estes WJ, Murray CA, Friedman RJ. Shoulder hemiarthroplasty for proximal humeral fractures. Orthop Clin North Am. 1998;29(3):467.
383. Hasan SA, Rauls RB, Cordell CL, Heinzelmann AD, Siegel ER. Pectoralis major insertional ratio in proximal humerus fractures: a method to reconstruct humeral head height in arthroplasty. Orthopedics. 2009;32(10):729.
384. Hasan SS, Leith JM, Campbell B, Kapil R, Smith KL, Matsen FA. Characteristics of unsatisfactory shoulder arthroplasties. J Shoulder Elbow Surg. 2002;11(5):431.
385. Hatzidakis AM, Shevlin MJ, Fenton DL, Curran-Everett D, Nowinski RJ, Fehringer EV. Angular-stable locked intramedullary nailing of two-part surgical neck fractures of the proximal part of the humerus. A multicenter retrospective observational study. JBJS. 2011; 93(23):2172.
386. Hawkins RJ, Bell RH, Gurr K. The three-part fracture of the proximal part of the humerus. Operative treatment. JBJS. 1986; 68(9):1410.
387. Hawkins RJ, Switlyk P. Acute prosthetic replacement for severe fractures of the proximal humerus. Clin Orthop Relat Res. 1993;289:156.
388. Heers G, Torchia ME. Shoulder hemi-arthroplasty in proximal humeral fractures. Orthopade. 2001;30(6):386.
389. Helwig P, Bahrs C, Epple B, Oehm J, Eingartner C, Weise K. Does fixed-angle plate osteosynthesis solve the problems of a fractured proximal humerus? A prospective series of 87 patients. Acta Orthopaedica. 2009;80(1):92.
390. Hempfing A, Leunig M, Ballmer FT, Hertel R. Surgical landmarks to determine humeral head retrotorsion for hemiarthroplasty in fractures. J Shoulder Elbow Surg. 2001;10(5):460.
391. Henshaw DR, Murthi AM, Levine WN. Percutaneous pins versus ORIF, or is HHR better?. Curr Opin Orthop. 2001;12(4):307.
392. Hente R, Kampshoff J, Kinner B, Fuchtmeier B, Nerlich M. Treatment of dislocated 3- and 4-part fractures of the proximal humerus with an angle-stabilizing fixation plate. Unfallchirurg. 2004;107(9):769.
393. Hepp P, Lill H, Bail H, Korner J, Niederhagen M, Haas NP, Josten C, Duda GN. Where should implants be anchored in the humeral head? Clin Orthop Relat Res. 2003;415:139.
394. Hepp P, Josten C. Biology and biomechanics in osteosynthesis of proximal humerus fractures. Eur J Trauma Emerg Surg. 2007;33(4):337.
395. Hepp P, Theopold J, Osterhoff G, Marquass B, Voigt C, Josten C. Bone quality measured by the radiogrammetric parameter "cortical index" and reoperations after locking plate osteosynthesis in patients sustaining proximal humerus fractures. Arch Orthop Trauma Surg. 2009;129(9):1251.
396. Hepp P, Theopold J, Voigt C, Engel T, Josten C, Lill H. The surgical approach for locking plate osteosynthesis of displaced proximal humeral fractures influences the functional outcome. JShoulder Elbow Surg. 2008;17(1):21.
397. Hernigou P, Duparc F, Hernigou A. Determining humeral retroversion with computed tomography. JBJS. 2002;84-A(10):1753.
398. Hernigou P, Germany W. Unrecognized shoulder joint penetration during fixation of proximal fractures of the humerus. Acta Orthop Scand. 2002;73(2):140.
399. Herold F, Wirtz C, Popp AG. Pectoral head height and tuberosity fixation with a tubular plate to restore proximal anatomy in hemiarthroplasty for irreparable proximal humeral fractures. Technique and first clinical results. Swiss Med Wkly. 2009;139(23-24):41S.
400. Herscovici D, Saunders DT, Johnson MP, Sanders R, DiPasquale T. Percutaneous fixation of proximal humeral fractures. Clin Orthop Relat Res. 2000;375:97.
401. Hertel R, Hempfing A, Stiehler M, Leunig M. Predictors of humeral head ischemia after intracapsular fracture of the proximal humerus. J Shoulder Elbow Surg. 2004;13(4):427.
402. Hessler C, Schmucker U, Matthes G, Ekkernkamp A, Gutschow R, Eggers C. Results after treatment of instable fractures of the proximal humerus using a fixed-angle plate. Unfallchirurg. 2006;109(10):867.
403. Hessmann M, Baumgaertel F, Gehling H, Klingelhoeffer I, Gotzen L. Plate fixation of proximal humeral fractures with indirect reduction: surgical technique and results utilizing three shoulder scores. Injury. 1999;30(7):453.
404. Hessmann M, Gehling H, Gotzen L. Proximal humerus fracture in advanced age. Langenbecks Archiv fur Chirurgie. 1996;113:907.
405. Hessmann M, Gotzen L, Gehling H, Baumgaertel F, Klingelhoeffer I. Operative treatment of displaced proximal humeral fractures: two-year results in 99 cases. Acta Chir Belg. 1998;98(5):212.
406. Hessmann MH, Blum J, Hofmann A, Kuchle R, Rommens PM. Internal Fixation of Proximal Humeral Fractures: Current Concepts. Eur J Trauma. 2003;29(5):253.
407. Hessmann MH, Korner J, Hofmann A, Sternstein W, Rommens PM. Angle-fixed plate fixation or double-plate osteosynthesis in fractures of the proximal humerus: A biomechanical study. Biomed Tech. 2008;53(3):130.
408. Hessmann MH, Sternstein W, Blum J, Krummenauer F, Rommens PM. Osteosynthesis of proximal humeral fractures with angular-stable plate fixation. Aktuelle Traumatol. 2003;33(1):2.
409. Hessmann MH, Sternstein W, Krummenauer F, Hofmann A, Rommens PM. Internal fixation of proximal humerus fractures. Chirurg. 2005;76(2):167.
410. Hessmann MH, Hansen WSM, Krummenauer F, Pol TF, Rommens M. Locked plate fixation and intramedullary nailing for proximal humerus fractures: A biomechanical evaluation. J Trauma Injury Infect Crit Care. 2005;58(6):1194.
411. Hessmann MH, Sternstein W, Mehler D, Korner J, Hofmann A, Rommens PM. Are angle-fixed implants with elastic properties advantageous for the internal fixation of proximal humerus fractures? Biomed Tech. 2004;49(12):345.
412. Hettrich CM, Boraiah S, Dyke JP, Neviaser A, Helfet DL, Lorich DG. Quantitative assessment of the vascularity of the proximal part of the humerus. JBJS. 2010;92(4):943.
413. Hettrich CM, Neviaser A, Beamer BS, Paul O, Helfet DL, Lorich DG. Locked plating of the proximal humerus using an endosteal implant. J Orthop Trauma. 2012;26(4):212.
414. Hinsenkamp M, Burny F, Andrianne Y. External fixation of the fracture of the humerus. A review of 164 cases. Orthopedics. 1984;7(8):1309.
415. Hintermann B, Trouillier HH, Schafer D. Rigid internal fixation of fractures of the proximal humerus in older patients. JBJS (Br). 2000;82(8):1107.
416. Hirschmann MT, Fallegger B, Amsler F, Regazzoni P, Gross T. Clinical longer-term results after internal fixation of proximal humerus fractures with a locking compression plate (PHILOS). J Orthop Trauma. 2011;25(5):286.
417. Hirschmann MT, Quarz V, Audige L, Ludin D, Messmer P, Regazzoni P, Gross T. Internal fixation of unstable proximal humerus fractures with an anatomically preshaped interlocking plate: A clinical and radialogic evaluation. J Trauma Injury Infect Crit Care. 2007;63(6):1314.
418. Hockings M, Haines JF. Least possible fixation of fractures of the proximal humerus. Injury. 2003;34(6):443.
419. Hodgson S. Proximal humerus fracture rehabilitation. Clin Orthop Relat Res. 2006;442:131.
420. Hodgson SA, Mawson SJ, Saxton JM, Stanley D. Rehabilitation of two-part fractures of the neck of the humerus (two-year follow-up). J Shoulder Elbow Surg. 2007;16(2):143.
421. Hodgson SA, Mawson SJ, Stanley D. Rehabilitation after two-part fractures of the neck of the humerus. JBJS (Br). 2003; 85(3):419.
422. Hoellen IP, Bauer G, Holbein O. Prosthetic humeral head replacement in dislocated humerus multi-fragment fracture in the elderly--an alternative to minimal osteosynthesis? Zentralbl Chir. 1997;122(11):994.
423. Hoffmann R, Khodadadyan C, Raschke M, Melcher I, Maitino PD, Haas NP. Retrograde intramedullary wire-fixation of proximal humerus fractures in the elderly. Results of a prospectively documented study. Zentralbl Chir. 1998;123(11):1232.
424. Hoffmeister E. Cannulated screws in percutaneous fixation of severe proximal humeral fractures: is technique safe and effective? Lippincotts Bone Joint Newsl. 2009;15(7):73.
425. Hoffmeister E. Outcomes examined in IM nailing of proximal humeral fractures. Lippincotts Bone Joint Newsl. 2009;15(9):97.
426. Hoffmeyer P. The operative management of displaced fractures of the proximal humerus. JBJS (Br). 2002;84(4):469.
427. Holbein O, Bauer G, Hoellen I, Keppler P, Hehl G, Kinzl L. Is primary endoprosthetic replacement of the humeral head an alternative treatment for comminuted fractures of the proximal humerus in elderly patients? Osteosynthese International. 1999;7(Suppl 2):207.
428. Holbein O, Hehl G, Keppler P, Kinzl L. Treatment of proximal humerus multiple fracture in old and very old people. Hefte zur der Unfallchirurg. 2000;275:207.
429. Hong-ran D, Xin S. Humeral head replacement versus locking plate in treating proximal humeral fractures. JCRTER. 2010;14(39):7390.
430. Horak J, Nilsson BE. Epidemiology of fracture of the upper end of the humerus. Clin Orthop Relat Res. 1975;112:250.
431. Horn J, Gueorguiev B, Brianza S, Steen H, Schwieger K. Biomechanical evaluation of two-part surgical neck fractures of the humerus fixed by an angular stable locked intramedullary nail. J Orthop Trauma. 2011;25(7):406.
432. Hoxie SC, Sperling JW, Cofield RH. Pulmonary embolism after operative treatment of proximal humeral fractures. J Shoulder Elbow Surg. 2007;16(6):782.
433. Hromadka R, Kubena AA, Pokorny D, Popelka S, Jahoda D, Sosna A. Attachments of muscles as landmarks for implantation of shoulder hemiarthoplasty in fractures. J Shoulder Elbow Surg. 2010;19(1):130.
434. Huang Q, Jiang X, Geng X, Wang M. Operative treatment of displaced proximal humeral fractures: follow-up and analysis of 31 patients'. Chung Hua Wai Ko Tsa Chih. 2000;38(10):728.
435. Huffman GR, Itamura JA, McGarry MH, Duong L, Gililland J, Tibone JE, Lee TQ. Neer Award 2006: Biomechanical assessment of inferior tuberosity placement during hemiarthroplasty for four-part proximal humeral fractures. J Shoulder Elbow Surg. 2008;17(2):189.
436. Iacobellis C, Fountzoulas K, Aldegheri R. Plating of proximal fracture of the humerus: a study of 30 cases. Musculoskeletal Surgery. 2011;95(Suppl 1):S43.
437. Iacobellis C, Serafini D, Aldegheri R. PHN for treatment of proximal humerus fractures: evaluation of 80 cases. Chirurgia Degli Organi di Movimento. 2009;93(2):47.
438. Ilchmann T, Ochsner PE, Wingstrand H, Jonsson K. Non-operative treatment versus tension-band osteosynthesis in three- and four-part proximal humeral fractures. A retrospective study of 34 fractures from two different trauma centers. Int Orthop. 1998;22(5):316.
439. Instrum K, Fennell C, Shrive N, Damson E, Sonnabend D, Hollinshead R. Semitubular blade plate fixation in proximal humeral fractures: a biomechanical study in a cadaveric model. J Shoulder Elbow Surg. 1998;7(5):462.
440. Itamura J, Dietrick T, Roidis N, Shean C, Chen F, Tibone J. Analysis of the bicipital groove as a landmark for humeral head replacement. J Shoulder Elbow Surg. 2002; 11(4): 322
441. Iulov VV, Golubev VG, Kkhir-Bek M. Miniinvasive technologies in treatment of intraarticular splittered fractures of the proximal shoulder. Khirurgiia. 2011; 43(9):39
442. Iyengar JJ, Devcic Z, Sproul RC, Feeley BT. Nonoperative treatment of proximal humerus fractures: A systematic review. J Orthop Trauma. 2011; 25(10): 612
443. Jaberg H, Warner JJ, Jakob RP. Percutaneous stabilization of unstable fractures of the humerus. J Bone Joint Surg Am. 1992; 74(4):508
444. Jain NB, Higgins LD, Losina E, Pietrobon R, Katz JN. Racial differences in type of surgical procedure performed for proximal humerus fractures. Eur J Orthop Surg Traumatol. 2011; 21(8):569
445. Jain NB, Warner JJ. Factors associated with cost and variation in surgical treatment of proximal humeral fractures. PM and R.Conference. 2011;3(10 SUPPL. 1):S216.
446. Jakob RP, Ganz R. Proximal humerus fractures. Helv Chir Acta. 1982; 48(5):595
447. Jakob RP, Miniaci A, Anson PS, Jaberg H, Osterwalder A, Ganz R. Four-part valgus impacted fractures of the proximal humerus. J Bone Joint Surg Br. 1991; 73(2):295
448. Jameson SS, James P, Howcroft DW, Serrano-Pedraza I, Rangan A, Reed MR, Candal-Couto J. Venous thromboembolic events are rare after shoulder surgery: analysis of a national database. J Shoulder Elbow Surg . 2011; 20(5):764
449. Jayankura M, Qui Du Phan D, Spinato L, Remy P, Cermak K, Schuind F. Treatment of severe proximal humeral fractures by proximal nailing (Telegraph). A prospective preliminary study. Rev Med Brux. 2011;32(6 SUPPL):S58.
450. Jerosch J. Hemireplacement after proximal humerus fractures. Physik Med Rehabilitationsmed Kurort. 2005; 15(2):105
451. Jiang CY, Huang Q, Geng XS, Wang MY, Rong GW. Percutaneous pinning for the treatment of proximal humerus fractures. Chung Hua Wai Ko Tsa Chih. 2004; 42(12):725
452. Jiang CY, Wang MY, Rong GW. Biomechanical study of percutaneous pinning as the treatment of proximal humerus fractures. Chung Hua Wai Ko Tsa Chih. 2004; 42(6):343
453. Jiang CY, Wang MY, Rong GW. Humeral head replacement for the complex treatment of proximal humerus fractures. Chung Hua Wai Ko Tsa Chih. 2003; 41(9):649
454. Jiang CY, Zhu YM, Wang MY, Rong GW. Biomechanical comparison of different pin configurations during percutaneous pinning for the treatment of proximal humeral fractures. J Shoulder Elbow Surg. 2007;16(2):235
455. Jiang P, Wei P, Chen L, Xia X. Minimally invasive percutaneous plate osteosynthesis technique for treatment of Neer II, III parts fracture of proximal humerus. Chung-Kuo Hsiu Fu Chung Chien Wai Ko Tsa Chih. 2011; 25(11):1330
456. Jobin CM, Galatz LM. Proximal Humerus Fractures: Pin, Plate, or Replace? Semin Arthroplasty. 2012;23(2):74
457. Jockel JA, Brunner A, Thormann S, Babst R. Elastic stabilisation of proximal humeral fractures with a new percutaneous angular stable fixation device (ButtonFix([REGISTERED])): a preliminary report. Arch Orthop Trauma Surg. 2010; 130(11):1397
458. Jones CB, Sietsema DL, Williams DK. Locked Plating of Proximal Humeral Fractures: Is Function Affected by Age, Time, and Fracture Patterns? Clin Orthop. 2011;469(12):3307
459. Jurik AG, Albrechtsen J. The use of computed tomography with two- and three-dimensional reconstructions in the diagnosis of three- and four-part fractures of the proximal humerus. Clin Radiol. 1994;49(11):800
460. Kabir K, Burger C, Fischer P, Weber O, Florczyk A, Goost H, Rangger C. Health status as an important outcome factor after hemiarthroplasty. J Shoulder Elbow Surg. 2009;18(1):75
461. Kachramanoglou C, Chidambaram R, Mok D. Four-part proximal humeral fractures: diagnosis with the 'sunset' sign on anteroposterior radiograph Ann R Coll Surg Engl. 2010; 92(7):599
462. Kamineni S, Ankem H, Sanghavi S. Anatomical considerations for percutaneous proximal humeral fracture fixation. Injury-Int J Care Inj. 2004; 35(11): 1133
463. Kannus P, Palvanen M, Niemi S, Parkkari J, Jarvinen M, Vuori I. Osteoporotic fractures of the proximal humerus in elderly Finnish persons: sharp increase in 1970-1998 and alarming projections for the new millennium. Acta Orthop Scand. 2000; 71(5):465
464. Kannus P, Palvanen M, Niemi S, Parkkari J, Jarvinen M, Vuori I. Increasing number and incidence of osteoporotic fractures of the proximal humerus in elderly people. BMJ. 1996;313(7064):1051
465. Kannus P, Palvanen M, Niemi S, Sievanen H, Parkkari J. Rate of proximal humeral fractures in older Finnish women between 1970 and 2007. Bone. 2009;44(4):656
466. Kapandji A. Osteosynthesis using the "palm-tree" nail technic in fractures of the surgical neck of the humerus. Ann Chir Main. 1989; 8(1):39
467. Karatosun V, Alekberov C, Baran O, Serin E, Alici E, Balci C. Open fractures of the proximal humerus treated with the Ilizarov method: 12 patients followed 3-8 years. Acta Orthop Scand. 2002; 73(4):460
468. Kasperczyk WJ, Engel M, Tscherne H. 4-fragment fracture of the proximal upper arm. Unfallchirurg. 1993; 96(8):422
469. Kay SP, Amstutz HC. Shoulder hemiarthroplasty at UCLA. Clin Orthop Relat Res. 1988; (228):42
470. Kayalar M, Toros T, Bal E, Ozaksar K, Gurbuz Y, Ademoglu Y. The importance of patient selection for the treatment of proximal humerus fractures with percutaneous technique. Acta Orthop Traumatol Turc. 2009;43(1):35
471. Kazakos K, Lyras DN, Galanis V, Verettas D, Psillakis I, Chatzipappas CH, Xarchas K. Internal fixation of proximal humerus fractures using the Polarus intramedullary nail. Arch Orthop Trauma Surg. 2007; 127(7):503
472. Kazitskii VM, Beider IL. A soft bandage for immobilizing the proximal part of the humerus. Ortop Travmatol Protez. 1988;(8):64
473. Kedgley AE, DeLude JA, Drosdowech DS, Johnson JA, Bicknell RT. Humeral head translation during glenohumeral abduction following computer-assisted shoulder hemiarthroplasty. J Bone Joint Surg Br. 2008;90(9):1256
474. Keene JS, Huizenga RE, Engber WD, Rogers SC. Proximal humeral fractures. A correlation of residual deformity with long-term function. Orthopedics. 1983: 6(2):173
475. Keser S, Bolukbasi S, Bayar A, Kanatli U, Meray J, Ozdemir H. Proximal humeral fractures with minimal displacement treated conservatively Int Orthop. 2004; 28(4):231
476. Kettler M, Biberthaler P, Braunstein V, Zeiler C, Kroetz M, Mutschler W. Treatment of proximal humeral fractures with the PHILOS angular stable plate. Presentation of 225 cases of dislocated fractures. Unfallchirurg. 2006;109(12):1032
477. Khan LA, Robinson CM, Will E, Whittaker R . Assessment of axillary nerve function and functional outcome after fixation of complex proximal humeral fractures using the extended deltoid-splitting approach. Injury. 2009;40(2):181
478. Khan SA. Helix wire osteosynthesis of proximal humerus fractures: Unacceptably high rate of failure. Acta Orthop Belg. 2008;74(1):13
479. Khodadadyan-Klostermann C, Raschke M, Fontes R, Melcher I, Sossan A, Bagchi K, Haas N. Treatment of complex proximal humeral fractures with minimally invasive fixation of the humeral head combined with flexible intramedullary wire fixation - introduction of a new treatment concept. Langenbecks Arch Surg. 2002;387(3-4):153
480. Khodadadyan-Klostermann C, Raschke M, Steenlage E, Von Seebach M, Melcher I, Haas N. Intramedullary wiring of proximal humerus fractures: Results of a minimally invasive treatment concept. European Journal of Trauma. 2002;28(4):234
481. Kiaer T, Larsen CF, Blicher J. Proximal fractures of the humerus. An epidemiological and descriptive study of fractures. Ugeskr Laeger. 1986;148(30):1894
482. Kienapfel H, Wilke A, Dorner P, Jurgensen R, Prinz H, Hettel A, Swain R, Griss P. Systemic effect of recombinant factor XIII and factor XIII concentrate on implant fixation strength and bone ingrowth. Z Orthop Ihre. 1995;133(5):394
483. Kilcoyne RF, Shuman WP, Matsen FA, Morris M, Rockwood CA. The Neer classification of displaced proximal humeral fractures: spectrum of findings on plain radiographs and CT scans. AJR. 1990;154(5):1029
484. Kilic B, Uysal M, Cinar BM, Ozkoc G, Demirors H, Akpinar S. Early results of treatment of proximal humerus fractures with the PHILOS locking plate. Acta Orthopaedica et Traumatologica Turcica 2008;42(3):149
485. Kim KC, Rhee KJ, Shin HD. Tension band sutures using a washer for a proximal humerus fracture. J Trauma-Injury Infect. 2008;64(4):1136
486. Kim SH, Lee YH, Chung SW, Shin SH, Jang WY, Gong HS, Baek GH. Outcomes for four-part proximal humerus fractures treated with a locking compression plate and an autologous iliac bone impaction graft. Injury. 2012;43(10): 1724
487. Kinzl L, Eisele R, Liener UC. The treatment of proximal humerus fractures with intramedullary nails. Aktuelle Traumatol. 2005;35(3):108
488. Kitson J, Booth G, Day R. A biomechanical comparison of locking plate and locking nail implants used for fractures of the proximal humerus. J Shoulder Elbow Surg. 2007;16(3):362
489. Klein M, Juschka M, Hinkenjann B, Scherger B, Ostermann PA. Treatment of comminuted fractures of the proximal humerus in elderly patients with the Delta III reverse shoulder prosthesis. J Orthop Trauma. 2008;22(10):698
490. Klepps SJ, Miller SL, Lin J, Gladstone J, Flatow EL. Determination of radiographic guidelines for percutaneous fixation of proximal humerus fractures using a cadaveric model. Orthopedics. 2007;30(8):636
491. Klitscher D, Blum J, Andreas D, Hessmann M, Kuechle R, du Prel JB, Rommens PM. Osteosynthesis of proximal humeral fractures with the fixed angle PHILOS-plate. Eur J Trauma Emerg Surg. 2008; 34(1):29
492. Ko JY, Yamamoto R. Surgical treatment of complex fracture of the proximal humerus. Clin Orthop Relat Res. 1996;(327):225
493. Kocialkowski A, Wallace WA. Closed percutaneous K-wire stabilization for displaced fractures of the surgical neck of the humerus. Injury. 1990;21(4):209
494. Koenigshausen M, Kuebler L, Godry H, Citak M, Schildhauer TA, Seybold D. Clinical outcome and complications using a polyaxial locking plate in the treatment of displaced proximal humerus fractures. A reliable system? Injury-Int J Care Inj. 2012; 43(2):223
495. Koesling C. Consequences of falls: upper arm fractures and shoulder joint replacement [German]. ERGOTHERAPIE REHABIL. 2004; 43(9):15
496. Kohler A, Simmen HP, Duff C, Kach K, Trentz O. Unconventional applied implants in osteosynthesis of subcapital humerus fractures. Swiss Surgery. 1995;1(2):114
497. Kohler A, Simmen HP, Duff C, Kossmann T, Trentz O. Osteosynthesis of proximal humerus fractures with unconventional implants. Helv Chir Acta. 1993; 59(4): 679
498. Kohnlein HE, Hellerer O, Schreiber JR. Indication for surgery in closed humeral fractures in the proximal and middle third. Munch Med Wochenschr. 1985; 127(25):645
499. Koike Y, Komatsuda T, Sato K. Internal fixation of proximal humeral fractures with a Polarus humeral nail. Eur J Orthop Surg. 2008; 9(3):135
500. Kollig E, Kutscha-Lissberg F, Roetman B, Dielenschneider D, Muhr G. Complex fractures of the humeral head: Which long-term results can be expected? Zentralbl Chir. 2003; 128(2):111
501. Kong LY, Liu RB, Zhou HD. Treatment of proximal fracture of humerus by percutaneous antegrade Kirschner pinning. Zhongguo gu shang. 2008; 21(6):453
502. Konrad G, Bayer J, Hepp P, Voigt C, Oestern H, Kaab M, Luo C, Plecko M, Wendt K, Kostler W, Sudkamp N. Open reduction and internal fixation of proximal humeral fractures with use of the locking proximal humerus plate: Surgical technique. JBJS. 2010;92(SUPPL. 1 PART 1):85.
503. Konrad G, Audige L, Lambert S, Hertel R, Suedkamp NP. Similar Outcomes for Nail versus Plate Fixation of Three-part Proximal Humeral Fractures. Clin Orthop. 2012;470(2):602
504. Konrad G, Hirschmueller A, Audige L, Lambert S, Hertel R, Suedkamp NP. Comparison of two different locking plates for two-, three- and four-part proximal humeral fractures-results of an international multicentre study. Int Orthop. 2012; 36(5):1051
505. Kontakis G, Koutras C, Tosounidis T, Giannoudis P. Early management of proximal humeral fractures with hemiarthroplasty A SYSTEMATIC REVIEW J.Bone Joint Surg Br. 2008; 90B(11):1407
506. Kontakis G, Tosounidis T, Galanakis I, Megas P. Prosthetic replacement for proximal humeral fractures. Injury. 2008; 39(12):1345
507. Kontakis GM, Tosounidis TI, Christoforakis Z, Hadjipavlou AG. Early management of complex proximal humeral fractures using the Aequalis fracture prosthesis A TWO- TO FIVE-YEAR FOLLOW-UP REPORT. J Bone Joint Surg Br. 2009; 91B(10):1335
508. Kopchev I, Kanev K. Thread fixation of bone fragments after Girgolav in fractures of the upper humeral epimetaphysis. Ortopediya i Travmatologiya. 1977; 14(3):189
509. Korkmaz MF, Aksu N, Goegus A, Debre M, Kara AN, Isiklar ZU. The results of internal fixation of proximal humeral fractures with the PHILOS locking plate. Acta Orthop Traumatol Turc. 2008;42(2):97
510. Kosaka T, Yamamoto K. Long-term results after treatment of humeral neck fractures using modified Hackethal bundle nailing. West Indian Med J. 2011;60(1):82
511. Kostler W, Strohm PC, Sudkamp NP. Proximal humerus fracture in advanced age - Treatment with fixed angle plate osteosynthesis Chirurg. 2003;74(11):985
512. Koukakis A, Apostolou CD, Taneja T, Korres DS, Amini A. Fixation of proximal humerus fractures using the PHILOS plate. Clin Orthop. 2006;(442):115
513. Koval KJ, Blair B, Takei R, Kummer FJ, Zuckerman JD. Surgical neck fractures of the proximal humerus: a laboratory evaluation of ten fixation techniques. J Trauma-Injury Infect. 1996; 40(5):778
514. Koval KJ, Gallagher MA, Marsicano JG, Cuomo F, McShinawy A, Zuckerman JD. Functional outcome after minimally displaced fractures of the proximal part of the humerus. J Bone Joint Surg Am. 1997; 79(2):203
515. Kovalev PV, Dubrovin GM, Gorban EV. Biomechanical rationale of the tense screw-pin osteosynthesis of proximal humerus fractures. Voen Med. 2011; 332(4):47
516. Krakovic M, Schmidt A, El Sigai A. Indication for surgery in shoulder-joint proximal fractures of the humerus and results. Monatsschr Unfallheilkd. 1975; 78(7):326
517. Kralinger F, Gschwentner M, Wambacher M, Smekal V, Haid C. Proximal humeral fractures: what is semi-rigid? Biomechanical properties of semi-rigid implants, a biomechanical cadaver based evaluation. Arch Orthop Trauma Surg. 2008; 128(2):205
518. Kralinger F, Irenberger A, Lechner C, Wambacher M, Golser K, Sperner G. Comparison of open versus percutaneous treatment for humeral head fracture. Unfallchirurg. 2006; 109(5):406
519. Kralinger F, Unger S, Wambacher M, Smekal V, Schmoelz W. The medial periosteal hinge, a key structure in fractures of the proximal humerus: a biomechanical cadaver study of its mechanical properties. J Bone Joint Surg Br. 2009; 91(7):973
520. Krappinger D, Roth T, Gschwentner M, Suckert A, Blauth M, Hengg C, Kralinger F. Preoperative assessment of the cancellous bone mineral density of the proximal humerus using CT data. Skeletal Radiol. 2012; 41(3):299
521. Krappinger D, Bizzotto N, Riedmann S, Kammerlander C, Hengg C, Kralinger FS. Predicting failure after surgical fixation of proximal humerus fractures. Injury-Int J Care Inj. 2011; 42(11):1283
522. Krause FB, Gathmann S, Gorschewski O. The use of intramedullary Helix wire for the treatment of proximal humerus fractures. J Orthop Trauma. 2008; 22(2):96
523. Krause FG, Huebschle L, Hertel R. Reattachment of the tuberosities with cable wires and bone graft in hemiarthroplasties done for proximal humeral fractures with cable wire and bone graft: 58 patients with a 22-month minimum follow-up. J Orthop Trauma. 2007; 21(10):682
524. Krishnan SG, Lin KC, Burkhead WZ. 'Pins, plates, and prostheses': Current concepts in treatment of fractures of the proximal humerus. Curr Opin Orthop. 2007; 18(4):380
525. Krishnan SG, Pennington SD, Burkhead WZ, Boileau P. Shoulder arthroplasty for fracture: Restoration of the "gothic arch". Tech Should Elb Surg. 2005; 6(2):57
526. Krishnan SG, Reineck JR, Bennion PD, Feher L, Burkhead WZ. Shoulder arthroplasty for fracture: does a fracture-specific stem make a difference? Clin Orthop Relat Res. 2011; 469(12):3317
527. Krishnan SG, Bennion PW, Reineck JR, Burkhead WZ. Hemiarthroplasty for Proximal Humeral Fracture: Restoration of the Gothic Arch. Orthop Clin North Am. 2008; 39(4):441
528. Kristiansen B. Treatment of displaced fractures of the proximal humerus: transcutaneous reduction and Hoffmann's external fixation. Injury. 1989; 20(4):195
529. Kristiansen B. External fixation of proximal humerus fracture. Clinical and cadaver study of pinning technique. Acta Orthop Scand. 1987; 58(6):645
530. Kristiansen B, Andersen UL, Olsen CA, Varmarken JE. The Neer classification of fractures of the proximal humerus. An assessment of interobserver variation. Skeletal Radiol. 1988; 17(6):420
531. Kristiansen B, Angermann P, Larsen TK. Functional results following fractures of the proximal humerus. A controlled clinical study comparing two periods of immobilization. Arch Orthop Trauma Surg. 1989; 108(6):339
532. Kristiansen B, Barfod G, Bredesen J, Erin-Madsen J, Grum B, Horsnaes MW, Aalberg JR. Epidemiology of proximal humeral fractures. Acta Orthop Scand. 1987; 58(1):75
533. Kristiansen B, Christensen S. Fractures of the proximal end of the humerus caused by convulsive seizures. Injury. 1984; 16(2):108
534. Kristiansen B, Christensen SW. Proximal humeral fractures. Late results in relation to classification and treatment. Acta Orthop Scand. 1987; 58(2):124
535. Kristiansen B, Christensen SW. Plate fixation of proximal humeral fractures. Acta Orthop Scand. 1986; 57(4):320
536. Kristiansen B, Kofoed H. Transcutaneous reduction and external fixation of displaced fractures of the proximal humerus. A controlled clinical trial. J Bone Joint Surg Br. 1988; 70(5):821
537. Kristiansen B, Kofoed H. Displaced proximal humeral fractures treated by external fixation. Rev Chir Orthop Reparatrice Appar Mot. 1988; 74(Suppl 2):290
538. Kristiansen B, Kofoed H. External fixation of displaced fractures of the proximal humerus. Technique and preliminary results. J Bone Joint Surg Br. 1987; 69(4): 643
539. Kristiansen B, Pallesen P, Torholm C. Hemi-alloplasty in the treatment of proximal humeral fractures. Ugeskr Laeger. 1994; 156(45):6683
540. Krivohlavek M, Lukas R, Taller S, Sram J. Use of angle-stable implants for proximal humeral fractures: Prospective study. Acta Chir Orthop Traumatol Cech. 2008; 75(3):212
541. Kuchle R, Hofmann A, Hessmann M, Rommens PM. The cloverleaf plate for osteosythesis of humeral head fractures. Definition of the current position. Unfallchirurg. 2006; 109(12):1017
542. Kuhlmann T, Hofmann T, Seibert O, Gundlach G, Schmidt-Horlohe K, Hoffmann R. Operative treatment of proximal humeral four-part fractures in elderly patients: comparison of two angular-stable implant systems. Z Orthop Unfall. 2012; 150(2):149
543. Kumar V, Datir S, Venkateswaran B. Intramedullary nailing for displaced proximal humeral fractures. J Orthop Surg Res. 2010; 18(3):324
544. Kwon BK, Goertzen DJ, O'Brien PJ, Broekhuyse HM, Oxland TR. Biomechanical evaluation of proximal humeral fracture fixation supplemented with calcium phosphate cement. J Bone Joint Surg Am. 2002; 84-A(6):951
545. Kwon YW, Zuckerman JD. Outcome after treatment of proximal humeral fractures with humeral head replacement. Instr Course Lect. 2005; 54:363
546. Laflamme GY, Rouleau DM, Berry GK, Beaumont PH, Reindl R, Harvey EJ. Percutaneous humeral plating of fractures of the proximal humerus: results of a prospective multicenter clinical trial. J Orthop Trauma. 2008; 22(3):153
547. Lahm A, Roesgen M. T-plate osteosynthesis in combination with tension band for proximal humerus fractures. Aktuelle Traumatol. 1997; 27(4):144
548. Lahm A, Roesgen M. Minimal osteosynthesis with tension band for proximal humerus fractures. Aktuelle Traumatol. 1996; 26(1):22
549. Laminger KA. Minimally invasive internal fixation of proximal humeral fractures with a helix wire. Oper Orthop Traumatol. 2004; 16(3):253
550. Laminger KA, Traxler H. Osteosynthesis of subcapital fractures of the humerus. Treatment of proximal humerus fractures by using a semirigid titanium helix. Unfallchirurgie. 1999; 25(3-4):154-164
551. Lanting B, MacDermid J, Drosdowech D, Faber KJ. Proximal humeral fractures: a systematic review of treatment modalities J Shoulder Elbow Surg. 2008; 17(1):42
552. Lau TW, Leung F, Chan CF, Chow SP. Minimally invasive plate osteosynthesis in the treatment of proximal humeral fracture. Int Orthop. 2007; 31(5): 657
553. Law TW, Leung FK, Lu Y. Application of minimally invasive locking compression plate in treatment of proximal humeral fractures. Zhongguo Xiu Fu Chong Jian Wai Ke Za Zhi. 2009; 23(11):1282
554. Le Bellec Y, Masmejean E, Cottias P, Alnot JY, Huten D. Internal fixation of proximal humerus fracture by "palm tree" pinning. Rev Chir Orthop Reparatrice Appar Mot. 2002; 88(4):342
555. Le Huec JC, Liquois F, Schaeverbeke T, Chauveaux D, Le Rebeller A. Intramedullary nailing osteosynthesis with epiphyseal fixation of fractures of the proximal humerus in adults 1 to 3-year follow-up. Acta Orthop Belg. 1992; 58(2):170
556. Lee CK, Hansen HR. Post-traumatic avascular necrosis of the humeral head in displaced proximal humeral fractures. J Trauma-Injury Infect Crit Care. 1981; 21(9):788
557. Lee CW, Shin SJ. Prognostic factors for unstable proximal humeral fractures treated with locking-plate fixation. J Shoulder Elbow Surg. 2009; 18(1):83
558. Lee SH, Dargent-Molina P, Breart G, Group EPIDOS, Study Epidemiologie de l'Osteoporose. Risk factors for fractures of the proximal humerus: results from the EPIDOS prospective study. J Bone Miner Res. 2002; 17(5):817
559. Lefevre-Colau MM, Babinet A, Fayad F, Fermanian J, Anract P, Roren A, Kansao J, Revel M, Poiraudeau S. Immediate mobilization compared with conventional immobilization for the impacted nonoperatively treated proximal humeral fracture - A randomized controlled trial. J Bone Joint Surg Am. 2007; 89A(12):2582
560. Leigheb M, De Falco G, Ceffa R, Grassi FA. Fixation of proximal humerus fractures with Polarus intramedullary nail. J Orthop Surg Res. 2011; 12:S8
561. Lenarz C, Shishani Y, McCrum C, Nowinski RJ, Edwards TB, Gobezie R. Is reverse shoulder arthroplasty appropriate for the treatment of fractures in the older patient? Early observations. Clin Orthop Relat Res. 2011; 469(12):3324
562. Lentz W, Meuser P. The treatment of fractures of the proximal humerus. Arch Orthop Trauma Surg. 1980; 96(4):283
563. Leonard M, Mokotedi L, Alao U, Glynn A, Dolan M, Fleming P. The use of locking plates in proximal humeral fractures: Comparison of outcome by patient age and fracture pattern. Int J Shoulder Surg. 2009; 3(4):85
564. Lervick GN, Carroll RM, Levine WN. Complications after hemiarthroplasty for fractures of the proximal humerus. Instr Course Lect. 2003; 52:3
565. Lescheid J, Zdero R, Shah S, Kuzyk PRT, Schemitsch EH. The Biomechanics of Locked Plating for Repairing Proximal Humerus Fractures With or Without Medial Cortical Support. J Trauma-Injury Infect Crit Care. 2010; 69(5):1235
566. Lever JP, Aksenov SA, Zdero R, Ahn H, Mckee MD, Schemitsch EH. Biomechanical analysis of plate osteosynthesis systems for proximal humerus fractures. J Orthop Trauma. 2008; 22(1):23
567. Levine WN, Connor PM, Yamaguchi K, Self EB, Arroyo JS, Pollock RG, Flatow EL, Bigliani LU. Humeral head replacement for proximal humeral fractures. Orthopedics. 1998; 21(1):68
568. Levy JC. Reverse shoulder replacement compared with hemiarthroplasty for proximal humeral fractures. Curr Orthop Pract. 2010; 21(5):443
569. Leyshon RL. Closed treatment of fractures of the proximal humerus. Acta Orthop Scand. 1984; 55(1):48
570. Li J, Chen GB, Xie ZJ, Wang HT. Treatment of humerus surgical neck fracture (Neer IV) with proximal humeral anatomical plate. Journal of Dalian Medical University. 2010; 32(2):201
571. Li L, Huang GP, Xiang Z, Huang FG, Cen SQ, Zhong G, Zhang SQ, Yang TF, Wang GL. A medium-term analysis on of therapeutic effects of locking proximal humerus plate for the treatment of comminuted fractures of proximal humerus. Zhongguo gu shang. 2010; 23(9):661
572. Li SM, Yang KJ, Huang J. Percutaneous plate fixation of three and four-part proximal humerus fractures in elderly patients. Zhongguo gu shang. 2010; 23(4):298
573. Liao C, Wang P, Xie Y, Fan T, Li P, Liang W. Different surgical methods for treatment of senile osteoporotic comminuted proximal humerus fracture. Zhongguo Xiu Fu Chong Jian Wai Ke Za Zhi. 2009; 23(12):1443
574. Liberati MA, Crainz-Fossati E, Di Bisceglie F, Savoini M, Pagliantini C, Tigani D. Minimal invasive osteosynthesis for proximal humerus fractures with polarus humeral nail. J Orthop Traumatol. 2011; 12:S18
575. Liborio Menniti E, Brasil Filho R, Filardi Filho CS,Vinicius Baptista M, Salim Daher S. New technique for fixation of two and three-part proximal humerus fracture tension-band with two screws: Parachuting system. Rev Bras Ortop. 1997; 32(9):713
576. Liew AS, Johnson JA, Patterson SD, King GJ, Chess DG. Effect of screw placement on fixation in the humeral head. J Shoulder Elbow Surg. 2000; 9(5):423
577. Lill H, Bewer A, Korner J, Verheyden P, Hepp P, Krautheim I, Josten CH. Conservative treatment of displaced proximal humeral fractures. Zentralbl Chir. 2001; 126(3):205
578. Lill H, Hepp P, Gowin W, Oestmann JW, Korner J, Haas NP, Josten C, Duda GN. Age- and gender-related distribution of bone mineral density and mechanical properties of the proximal humerus. RoFo Fortschritte auf dem Gebiet der Rontgenstrahlen und der Bildgebenden Verfahren. 2002; 174(12):1544
579. Lill H, Hepp P, Korner J, Kassi JP, Verheyden AP, Josten C, Duda GN. Proximal humeral fractures: how stiff should an implant be? A comparative mechanical study with new implants in human specimens. Arch Othop Trauma Surg. 2003; 123(2-3):74
580. Lill H, Hepp P, Rose T, Konig K, Josten C. The angle stable Locking-Proximal-Humerus-Plate (LPHP (R)) for proximal humeral fractures using a small anterior-lateral-deltoid-splitting-approach - Technique and first results. Zentralbl Chir. 2004; 129(1):43
581. Lill H, Korner J, Glasmacher S, Hepp P, Just A, Verheyden P, Josten C. Crossed screw osteosynthesis of proximal humerus fractures. Unfallchirurg. 2001; 104(9):852
582. Lill H, Lange K, Prasse-Badde J, Schmidt A, Verheyden P, Echtermeyer V, Kuner EH. The T-plate for dislocated fractures of the proximal humerus. Unfallchirurgie. 1997; 23(5):183
583. Lill H, Katthagen C, Hertel A, Gille J, Voigt C. All-arthroscopic intramedullary nailing of 2-and 3-part proximal humeral fractures: a new arthroscopic technique and preliminary results. Arch Orthop Trauma Surg. 2012; 132(5):641
584. Lim TE, Ochsner PE, Marti RK, Holscher AA. The results of treatment of comminuted fractures and fracture dislocations of the proximal humerus. Neth J Surg. 1983; 35(4):139
585. Lin J. Effectiveness of locked nailing for displaced three-part proximal humeral fractures. J Trauma Injury Infect Crit Care. 2006; 61(2):363.
586. Lin J, Hou SH, Hang YS. Locked nailing for displaced surgical neck fractures of the humerus. J Trauma Injury Infect Crit Care. 1998;45(6):1051.
587. Lind T, Kroner K, Jensen J. The epidemiology of fractures of the proximal humerus. Arch Orthop Trauma Surg. 1989;108(5):285.
588. Linhart W, Ueblacker P, GroÃŸterlinden L, Kschowak P, Briem D, Janssen A, Hassunizadeh B, Schinke M, Windolf J, Rueger JM. Antegrade nailing of humeral head fractures with captured interlocking screws. J Orthop Trauma. 2008;22(3):S46.
589. Liu J, Li SH, Cai ZD, Lou LM, Wu X, Zhu YC, Wu WP. Outcomes, and factors affecting outcomes, following shoulder hemiarthroplasty for proximal humeral fracture repair. J Orthop Sci. 2011;16(5):565.
590. Liu KY, Chen TH, Shyu JF, Wang ST, Liu JY, Chou PH. Anatomic study of the axillary nerve in a Chinese cadaveric population: correlation of the course of the nerve with proximal humeral fixation with intramedullary nail or external skeletal fixation. Arch Orthop Trauma Surg. 2011;131(5):669.
591. Liu XW, Fu QG, Xu SG, Zhang CC, Su JC, Wang PF, Zhao DF, Li YH. Application of PHILOS plate with injectable artificial bone for the treatment of proximal humeral fractures in elderly patients. Zhongguo Gushang. 2010;23(3):180.
592. Liu Y, Yu GR, Gu JF. A two-year follow-up on the recovery outcomes of shoulder joint function in 18 patients following artificial shoulder replacement. Chin J Clin Rehab. 2005;9(22):8.
593. Liu YW, Kuang Y, Gu XF, Zheng YX, Li ZQ, Wei XE, Lu WD, Zhan HS, Shi YY.A study of proximal humerus fractures using close reduction and percutaneous minimally invasive fixation. Zhongguo Gushang. 2011;24(11):949.
594. Liu ZZ, Zhang GM, Ge T. Use of a proximal humeral internal locking system enhanced by injectable graft for minimally invasive treatment of osteoporotic proximal humeral fractures in elderly patients. Orthopaedic Audio-Synopsis Continuing Medical Education. 2011;3(4):253.
595. Loebenberg MI, Jones DA, Zuckerman JD. The effect of greater tuberosity placement on active range of motion after hemiarthroplasty for acute fractures of the proximal humerus. Bull Hosp Joint Dis. 2005;62(3-4):90.
596. Loew M, Heitkemper S, Parsch D, Schneider S, Rickert M. Influence of the design of the prosthesis on the outcome after hemiarthroplasty of the shoulder in displaced fractures of the head of the humerus. JBJS (Br). 2006;88B(3):345.
597. Logroscino G, Milano G, Ciriello V, De Ieso C, Izzo V, Fabbriciani C. "Univers" shoulder prosthesis in proximal humerus fractures. J Orthop Trauma (Conf.) 2011;12:S17.
598. Logroscino G, Milano G, Donati F, Deriu L, Fabbriciani C. Percutaneous fixation of proximal humerus fractures using a new fixation system. J Orthop Trauma (Conf.). 2011;12:S8.
599. Loitz D, Konnecker H, Illgner A, Reilmann H. Retrograde intramedullary nailing of humeral fractures with new implants. Analysis of 120 consecutive cases. Unfallchirurg. 1998;101(7):543.
600. Lu CC, Chang MW, Lin GT. Intramedullary pinning with tension-band wiring for surgical neck fractures of the proximal humerus in elderly patients. Kaohsiung J Med Sci. 2004;20(11):538.
601. Lu Y, Jiang CY, Zhu YM, Wang MY. Clinical research on proximal humeral fractures treated by locking proximal humeral plate. Chung-Hua Wai Ko Tsa Chih. 2007;45(20):1375.
602. Lu Y, Wang MY, Zhu YM, Jiang CY. Complications of the locking plate for displaced proximal humeral fractures. Chin Med J. 2010;123(19):2671.
603. Lubbeke A, Stern R, Grab B, Herrmann F, Michel JP, Hoffmeyer P. Upper extremity fractures in the elderly: consequences on utilization of rehabilitation care. Aging Clin Exp Res. 2005;17(4):276.
604. Lundberg BJ, Svenungson-Hartwig E, Wikmark R. Independent exercises versus physiotherapy in nondisplaced proximal humeral fractures. Scand J Rehabil Med. 1979;11(3):133.
605. Lungershausen W, Bach O, Lorenz CO. Locking plate osteosynthesis for fractures of the proximal humerus. Zentralbl Chir. 2003;128(1):28.
606. Lupo R, Rapisarda SA, Lauria S, Palmisciano G. Plates with angular stability: our personal experience in surgical treatment of fractures of the proximal extremity of the humerus. Chirurgia Degli Organi di Movimento. 2008; 91(2):97.
607. Luppino D, Santangelo G, Vicenzi G, Innao V, Capelli A. Fractures of the proximal end of the humerus of surgical importance (study of 40 cases). Chirurgia Degli Organi di Movimento. 1981;67(4):373.
608. Machani B, Sinopidis C, Brownson P, Papadopoulos P, Gibson J, Frostick SP. Mid term results of PlantTan plate in the treatment of proximal humerus fractures. Injury Int J Care Inj. 2006;37(3):269.
609. Mahadeva D, Dias RG, Deshpande SV, Datta A, Dhillon SS, Simons AW. The reliability and reproducibility of the Neer classification system--digital radiography (PACS) improves agreement. Injury. 2011;42(4):339.
610. Mahadeva D, Mackay DC, Turner SM, Drew S, Costa ML. Reliability of the Neer classification system in proximal humeral fractures: a systematic review of the literature. Eur J Orthop Surg Traumatol. 2008;18(6):415.
611. Majed A, Macleod I, Bull AMJ, Zyto K, Resch H, Hertel R, Reilly P, Emery RJH. Proximal humeral fracture classification systems revisited. J Shoulder Elbow Surg. 2011;20(7):1125.
612. Maldonado ZM, Seebeck J, Heller MOW, Brandt D, Hepp P, Lill H, Duda GN. Straining of the intact and fractured proximal humerus under physiological-like loading. J Biomech. 2003;36(12):1865.
613. Mallick A, Hearth M, Singh S, Pandey R. Modified rush pin technique for two- or three-part proximal humeral fractures. J Orthop Surg. 2008;16(3):285.
614. Manetti G, De Luca L, Sangiovanni M, Cardini M, Rossi U. Pinnig as an alternative treatment for proximal humerus fractures. J Orthop Trauma (Conf.) 2011;12:S7.
615. Maravic M, Taupin P, Landais P, Roux C. Proximal Humerus Fractures in Men. Epidemiology and Costs. J Bone Miner Res. 2008;23:S324.
616. Marco F, FrancZs A, Gallego P, De Francisco B, Otero R. Displaced fractures of the proximal humerus: Fixation or replacement? JBJS. 2004;86-B(SUPP_III):307.
617. Mariotti U, Bertignone L, Milano P, Sabra M. Non invasive treatment of proximal humeral fractures: Critical review of our results. Minerva Ortopedica e Traumatologica. 2001;52(4-5):211
618. Mariotti U, Motta P, Vassoney P, Grillo P, Caranzano F. Transcutaneous osteosyntesis: Screws, wires, fixers. Gazzetta Medica Italiana Archivio per le Scienze Mediche. 2007;166(3):105.
619. Martin C, Guillen M, Lopez G. Treatment of 2- and 3-part fractures of the proximal humerus using external fixation: a retrospective evaluation of 62 patients. Acta Orthopaedica. 2006;77(2):275.
620. Martinez AA, Cuenca J, Herrera A. Philos plate fixation for proximal humeral fractures. J Orthop Surg. 2009;17(1):10.
621. Martin-Hernandez C, Guillen-Soriano M, Ballester-Gimenez JJ, Acevedo-Medina GL, Fernandez-Esteve F. Treatment by external fixation of 2-part and 3-part proximal humeral fractures. Revista de Ortopedia y Traumatologia. 2006;50(5):335.
622. Martini M. The sub-deltoid approach to the metaphyseal region of the humerus. JBJS. 1976;58(3):377.
623. Matassi F, Angeloni R, Carulli C, Civinini R, Di Bella L, Redl B, Innocenti M. Locking plate and fibular allograft augmentation in unstable fractures of proximal humerus. Injury. 2012;43(11):1939.
624. Mathews J, Lobenhoffer P. The Targon PH nail as an internal fixator for unstable fractures of the proximal humerus. Operative Orthopadie und Traumatologie. 2007;19(3):55.
625. Mathews J, Lobenhoffer P. Results of intramedullary nailing of unstable proximal humeral fractures in geriatric patients with a new antegrade nail system. Unfallchirurg. 2004;107(5):372.
626. Mathison C, Chaudhary R, Beaupre L, Reynolds M, Adeeb S, Bouliane M. Biomechanical analysis of proximal humeral fixation using locking plate fixation with an intramedullary fibular allograft. Clin Biomech. 2010;25(7):642.
627. Matziolis D, Kaeaeb M, Zandi SS, Perka C, Greiner S. Surgical treatment of two-part fractures of the proximal humerus: comparison of fixed-angle plate osteosynthesis and Zifko nails. Injury. 2010;41(10):1041.
628. Mehlhorn AT, Schmal H, Sudkamp NP. Clinical evaluation of a new custom offset shoulder prosthesis for treatment of complex fractures of the proximal humerus. Acta Orthop Belg. 2006;72(4):387.
629. Meier RA, Messmer P, Regazzoni P, Rothfischer W, Gross T. Unexpected high complication rate following internal fixation of unstable proximal humerus fractures with an angled blade plate. J Orthop Trauma. 2006;20(4):253.
630. Mestdagh H, Butruille Y, Tillie B, Bocquet F. Results of the treatment of proximal humeral fractures by percutaneous nailing. Apropos of 142 cases. Ann Chir. 1984;38(1):5.
631. Meyer C, Alt V, Hassanin H, Heiss C, Stahl JP, Giebel G, Koebke J, Schnettler R. The arteries of the humeral head and their relevance in fracture treatment. Surg Radiol Anat. 2005;27(3):232.
632. Mica L, Frohlich T, Schwaller A, Vollenweider A, Rancan M. The Targon-PH Nail as an internal fixator for unstable capital humeral fractures in the elderly patient: A retrospective study of 39 patients. Acta Chirurgica Austriaca (Conf.). 2011;43:131.
633. Mighell MA, Jennings WB, Frankle MA. Technique for Unstable Two-Part Surgical Neck Proximal Humeral Fractures Utilizing an Intramedullary Staple Device: The Evan's Staple. J Shoulder Elbow Surg. 2003;4(2):84.
634. Mighell MA, Kolm GP, Collinge CA, Frankle MA. Outcomes of hemiarthroplasty for fractures of the proximal humerus. J Shoulder Elbow Surg. 2003;12(6):569.
635. Mihara K, Tsutsui H, Suzuki K, Makiuchi D, Nishinaka N, Yamaguchi K. New intramedullary nail for the surgical neck fracture of the proximal humerus in elderly patients. J Orthop Sci. 2008;13(1):56.
636. Mills HJ, Horne G. Fractures of the proximal humerus in adults. J Trauma Injury Infect Crit Care. 1985;25(8):801.
637. Minaev AN, Gorodnichenko AI, Uskov ON.The transosseous osteosynthesis of proximal humeral metaepiphyseal fractures in elderly patients. Khirurgiia. 2010;1:50.
638. Mittlmeie TWF, Stedtfeld HW, Ewert A, Beck M, Frosch B, Gradl G. Stabilization of proximal humeral fractures with an angular and sliding stable antegrade locking nail (Targon PH). J Bone Joint Surg Am Vol. 2003;85A:136.
639. Mo XF, He ZJ, Zhuo XM, Liu XY, Zeng XM, Guo JE, Zhou XM.Clinical analysis of 19 elderly patients with displaced proximal humeral two- and three-part fractures. Di Yi Junyi Daxue Xuebao. 2004;24(9):1080.
640. Moda SK, Chadha NS, Sangwan SS, Khurana DK, Dahiya AS, Siwach RC. Open reduction and fixation of proximal humeral fractures and fracture-dislocations. JBJS (Br). 1990;72(6):1050.
641. Moeckel BH, Dines DM, Warren RF, Altchek DW. Modular hemiarthroplasty for fractures of the proximal part of the humerus. JBJS. 1992;74(6):884.
642. Moen TC, Bigliani LU. Hemiarthroplasty for Four-Part Fractures of the Proximal Humerus. Oper Tech Orthop. 2011;21(1):94.
643. Monga P, Verma R, Sharma VK. Closed reduction and external fixation for displaced proximal humeral fractures. J Orthop Surg. 2009;17(2):142.
644. Monin S, van Innis F. Fractures of the proximal end of the humerus treated by the Kapandji centro-medullary nailing technic. A review of 21 cases. Acta Orthop Belg. 1999;65(2):176.
645. Monteiro GC, Ejnisman B, Andreoli CV, de Castro Pochini A, Olympio E. Results of Treatment of Proximal Humerus Fractures using Locking Plates. Acta Ortop Bras. 2011;19(2):69.
646. Moonot P, Ashwood N, Hamlet M. Early results for treatment of three- and four-part fractures of the proximal humerus using the PHILOS plate system. JBJS (Br). 2007;89(9):1206.
647. Guix JMM, Gonzalez AS, Brugalla JV, Carril EC, Banos FG. Proposed protocol for reading images of humeral head fractures. Clin Orthop Relat Res. 2006;448:225.
648. Guix JMM, Pedros JS, Serrano AC. Updated classification system for proximal humeral fractures. Clin Med Res. 2009;7(1-2):32.
649. Morelli RSS, de Souza Travizanuto RE. Proximal Humerus Fractures: Comparative Study of Two Different Fixation Methods. Acta Ortop Bras. 2010;18(2):79.
650. Morici D, Castellani GC, Biccire F. Four-part displaced proximal humeral fractures: treatment with hemiarthroplasty. Chirurgia Degli Organi di Movimento. 2003;88(4):411.
651. Moschinski D, Franke R, Moschinski A. Treatment of the displaced three- and four-segment fracture of the proximal humerus. Aktuel Chir. 1995;30(6):352.
652. Mourad L. Neer classification of fractures of the proximal humerus. Orthop Nur. 1997;16(2):76.
653. Mouradian WH. Displaced proximal humeral fractures. Seven years' experience with a modified Zickel supracondylar device. Clin Orthop Relat Res. 1986;212:209.
654. Movin T, Sjoden GO, Ahrengart L. Poor function after shoulder replacement in fracture patients. A retrospective evaluation of 29 patients followed for 2-12 years. Acta Orthop Scand. 1998;69(4):392.
655. Muckter H, Herzog L, Becker M, Vogel W, Meeder PJ, Buchholz J. Angle- and rotation-stable internal fixation of proximal humerus fractures with the humerus fixator plate. Early clinical experience with a newly developed implant. Chirurg. 2001;72(11):1327.
656. Mueller HA, Koudsi F. Osteosynthesis of fractures of the proximal end of the humerus and the results. Aktuelle Traumatol. 1978;8(2):143.
657. Muller B, Bonnaire F, Kuner EH. Dislocated fractures of the proximal humerus: Treatment concept, technique and results. Aktuelle Traumatol. 1998;28(2):61.
658. Muller F, Voithenleitner R, Schuster C, Angele P, Weigel B. Operative treatment of proximal humeral fractures with helix wire. Unfallchirurg. 2006;109(12):1041.
659. Munst P, Kuner EH. Osteosynthesis in dislocated fractures of the humerus head. Orthopade. 1992;21(2):121.
660. Murachovsky J, Ikemoto RY, Nascimento LG, Fujiki EN, Milani C, Warner JJ. Pectoralis major tendon reference (PMT): a new method for accurate restoration of humeral length with hemiarthroplasty for fracture. J Shoulder Elbow Surg. 2006;15(6):675.
661. Murena L, Turino S, Boscarino SV, Grassi FA, Cherubino P. Proximal humeral fracture treatment with locking plate and polyaxial screws. J Orthop Trauma (Conf.). 2011;12:S7.
662. Naidu SH, Bixler B, Capo JT, Moulton MJ, Radin A. Percutaneous pinning of proximal humerus fractures: a biomechanical study. Orthopedics. 1997;20(11):1073.
663. Nalbone L, Adelfio R, Butera M, D'Arienzo M. Shoulder replacement in the treatment of pluri-fragmentary and displaced fractures of the proximal humerus: Our experience. J Orthop Trauma (Conf.). 2011;12:S19.
664. Nanda R, Goodchild L, Gamble A, Campbell RS, Rangan A. Does the presence of a full-thickness rotator cuff tear influence outcome after proximal humeral fractures?. J Trauma Injury Infect Crit Care. 2007;62(6):1436.
665. Naraen A, Faraj AA, Livesley PJ, Hambridge JE. A preliminary clinical experience with polarus nail for proximal humeral fractures. A pilot study on ten patients. Eur J Orthop Surg Traumatol. 2001;11(3):191.
666. Narvani A, Levy O. Minimal invasive flexible fixation for fractures of the proximal humerus. Ann Roll Surg Engl. 2010;92(8):635.
667. Neer CS. Four-segment classification of proximal humeral fractures: purpose and reliable use. J Shoulder Elbow Surg. 2002;11(4):389.
668. Neer CS. Displaced proximal humeral fractures. II. Treatment of three-part and four-part displacement. JBJS. 1970;52(6):1090.
669. Neer CS. Displaced proximal humeral fractures. I. Classification and evaluation. JBJS. 1970;52(6):1077.
670. Nerot C, Thoreux P, Hannouche D. Conservative treatment of proximal humeral fractures: Interobserver classification. Rev Chir Orthop Reparatrice Appar Mot. 1998;84(SUPPL. 1):129.
671. Neubauer T, Wagner M. Minimally invasive plate osteosynthesis on the proximal upper limb. Instructions for surgery. Unfallchirurg. 2009;112(11):997.
672. Neumann K, Muhr G, Breitfuss H. Primary humerus head replacement in dislocated proximal humeral fracture. Indications, technique, results. Orthopade. 1992;21(2):140.
673. Neumann K, Muhr G, Breitfuss H. Endoprosthesis in comminuted fractures of the proximal humerus. An acceptable alternative. Unfallchirurg. 1988;91(10):451.
674. Neverov VA, Khromov AA, Cherniaev SN, Egorov KS. Potentialities of blocking osteosynthesis for metaphyseal fractures of the humeral bone. Vestnik Khirurgii Imeni i - i – Grekova. 2008;167(6):52.
675. Neviaser AS, Hettrich CM, Beamer BS, Dines JS, Lorich DG. Endosteal strut augment reduces complications associated with proximal humeral locking plates. Clin Orthop Relat Res. 2011;469(12):3300.
676. Neviaser AS, Hettrich CM, Dines JS, Lorich DG. Rate of avascular necrosis following proximal humerus fractures treated with a lateral locking plate and endosteal implant. Arch Orthop Trauma Surg. 2011;131(12):1617.
677. Nijs S, Broos P. Outcome of shoulder hemiarthroplasty in acute proximal humeral fractures: a frustrating meta-analysis experience. Acta Orthop Belg. 2009;75(4):445.
678. Nijs S, Reuther F, Broos P. Primary fracture arthroplasty of the proximal humerus using a new and freely adjustable modular prosthesis combined with compression osteosynthesis of the tuberosities. Operative Orthopadie und Traumatologie. 2011;23(1):21.
679. Nijs S, Sermon A, Broos P. Intramedullary fixation of proximal humerus fractures: do locking bolts endanger the axillary nerve or the ascending branch of the anterior circumflex artery? A cadaveric study. Patient Saf Surg. 2008;2(1):33.
680. Noda M, Saegusa Y, Maeda T. Does the location of the entry point affect the reduction of proximal humeral fractures? A cadaveric study. Injury. 2011;42(Suppl 4):S35.
681. Nolan BM, Kippe MA, Wiater JM, Nowinski GP. Surgical treatment of displaced proximal humerus fractures with a short intramedullary nail. J Shoulder Elbow Surg. 2011;20(8):1241.
682. Norouzi M, Naderi MN, Komasi MH, Sharifzadeh SR, Shahrezaei M, Eajazi A. Clinical results of using the proximal humeral internal locking system plate for internal fixation of displaced proximal humeral fractures. Am J Orthop. 2012;41(5):E64.
683. Noyes MP, Kleinhenz B, Markert RJ, Crosby LA. Functional and radiographic long-term outcomes of hemiarthroplasty for proximal humeral fractures. J Shoulder Elbow Surg. 2011;20(3):372.
684. Obrebski M, Rapala K, Milecki M. Neer classification of the proximal humerus fractures based on our own material. Chir Narzadow Ruchu Ortop Pol. 2006;71(1):43.
685. Ochsner PE, Ilchmann T. Tension band osteosynthesis with absorbable cords in proximal comminuted fractures of the humerus. Unfallchirurg. 1991;94(10):508.
686. Ockert B, Braunstein V, Kirchhoff C, Korner M, Kirchhoff S, Kehr K, Mutschler W, Biberthaler P. Monoaxial versus polyaxial screw insertion in angular stable plate fixation of proximal humeral fractures: radiographic analysis of a prospective randomized study. J Trauma Injury Infect Crit Care. 2010;69(6):1545.
687. Ogawa K, Kobayashi S, Ikegami H. Retrograde intramedullary multiple pinning through the deltoid "V" for valgus-impacted four-part fractures of the proximal humerus. J Trauma Injury Infect Crit Care. 2011;71(1):238.
688. Olerud P, Ahrengart L, Ponzer S, Saving J, Tidermark J. Hemiarthroplasty versus nonoperative treatment of displaced 4-part proximal humeral fractures in elderly patients: a randomized controlled trial. J Shoulder Elbow Surg. 2011;20(7):1025.
689. Olerud P, Ahrengart L, Ponzer S, Saving J, Tidermark J. Internal fixation versus nonoperative treatment of displaced 3-part proximal humeral fractures in elderly patients: a randomized controlled trial. J Shoulder Elbow Surg. 2011;20(5):747.
690. Olerud P, Ahrengart L, Soderqvist A, Saving J, Tidermark J. Quality of life and functional outcome after a 2-part proximal humeral fracture: A prospective cohort study on 50 patients treated with a locking plate. J Shoulder Elbow Surg. 2010;19(6):814.
691. Olerud P, Tidermark J, Ponzer S, Ahrengart L, Bergstrom G. Responsiveness of the EQ-5D in patients with proximal humeral fractures. J Shoulder Elbow Surg. 2011;20(8);1200.
692. Olsson C, Nordquist A, Petersson CJ. Long-term outcome of a proximal humerus fracture predicted after 1 year - A 13-year prospective population-based follow-up study of 47 patients. Acta Orthop. 2005;76(3):397.
693. Olsson C, Nordqvist A, Petersson CJ. Increased fragility in patients with fracture of the proximal humerus: a case control study. Bone. 2004;34(6):1072.
694. Olsson C, Petersson C, Nordquist A. Increased mortality after fracture of the surgical neck of the humerus: a case-control study of 253 patients with a 12-year follow-up. Acta Orthop Scand. 2003;74(6):714.
695. Olsson C, Petersson CJ. Clinical importance of comorbidity in patients with a proximal humerus fracture. Clin Orthop. 2006;442:93.
696. Omid R, Galatz LM. Percutaneous pinning of proximal humerus fractures: A technique. Semin Arthroplasty. 2011;22(1):2.
697. Onder U, Blauth M, Kralinger F, Schmoelz W. Shoulder joint abduction motion test bench: A new shoulder test bench for in vitro experiments with active muscle force simulation. Biomed Tech. 2012;0(0):1.
698. Ong C, Bechtel C, Walsh M, Zuckerman JD, Egol KA. Three- and four-part fractures have poorer function than one-part proximal humerus fractures. Clin Orthop Relat Res. 2011;469(12):3292.
699. Orlowski J, Pomianowski S, Sawicki G. The management of patients over 65 years of age with proximal humerus fractures. Ortopedia Traumatologia Rehabilitacja. 2002;4(2):140.
700. Oster A, Pilgaard S. Proximal four-fragment fractures of the humerus. A follow-up investigation of 21 cases. Ugeskr Laeger. 1974;136(28):1567.
701. Osterhoff G, Hoch A, Wanner GA, H Simmen HP, Werner CM. Calcar comminution as prognostic factor of clinical outcome after locking plate fixation of proximal humeral fractures. Injury. 2012;43(10):1651.
702. Osterhoff G, Baumgartner D, Favre P, Wanner GA, Gerber H, Simmen HP, Werner CML. Medial support by fibula bone graft in angular stable plate fixation of proximal humeral fractures: an in vitro study with synthetic bone. J Shoulder Elbow Surg. 2011;20(5):740.
703. Osterhoff G, Diederichs G, Tami A, Theopold J, Josten C, Hepp P. Influence of trabecular microstructure and cortical index on the complexity of proximal humeral fractures. Arch Orthop Trauma Surg. 2012;132(4):509.
704. Osterhoff G, Ossendorf C, Wanner GA, Simmen HP, Werner CM. The calcar screw in angular stable plate fixation of proximal humeral fractures - a case study. J Orthop Surg Res. 2011;6:50.
705. Owsley KC, Gorczyca JT. Fracture displacement and screw cutout after open reduction and locked plate fixation of proximal humeral fractures [corrected]. JBJS. 2008;90(2):233.
706. Paavolainen P, Bjorkenheim JM, Slatis P, Paukku P. Operative treatment of severe proximal humeral fractures. Acta Orthop Scand. 1983;54(3):374.
707. Padua R, Padua L, Galluzzo M, Ceccarelli E, Alviti F, Castagna A. Position of shoulder arthroplasty and clinical outcome in proximal humerus fractures. Musculoskelet Surg. 2011;95(Suppl 1):S55.
708. Padua R, Bondi R, Ceccarelli E, Compi A, Padua L. Health-related quality of life and subjective outcome after shoulder replacement for proximal humeral fractures. J Shoulder Elbow Surg. 2008;17(2):261.
709. Palvanen M, Kannus P, Niemi S, Parkkari J. Update in the epidemiology of proximal humeral fractures. Clin Orthop. 2006;442:87.
710. Pan ZX, Qu LJ, Yang XM, Zhang HX, Cui Y, Sun C. Technical points of artificial humeral head replacement for the treatment of comminuted fracture of the proximal humerus in elderly patients. CRTER. 2010;14(35):6620.
711. Panagopoulos AM, Dimakopoulos P, Tyllianakis M, Karnabatidis D, Siablis D, Papadopoulos AX, Lambiris E, Kraniotis P, Sakellaropoulos G. Valgus impacted proximal humeral fractures and their blood supply after transosseous suturing. Int Orthop. 2004;28(6):333.
712. Papadopoulos P, Karataglis D, Stavridis SI, Petsatodis G, Christodoulou A. Mid-term results of internal fixation of proximal humeral fractures with the Philos plate. Injury Int Care Inj. 2009;40(12):1292.
713. Pappalardo S, Braidotti P, Ceretti M, Manauzzi E. Proximal Humerus Fractures: Clinical and Radiographical Results with Different Ostheosynthesis Techniques. Minerva Ortop Traumatol. 2009;60(2):99.
714. Park JY, An JW, Oh JH. Open intramedullary nailing with tension band and locking sutures for proximal humeral fracture: Hot air balloon technique. J Shoulder Elbow Surg. 2006;15(5):594.
715. Park JY, Kim JH, Lhee SH, Lee SJ. The importance of inferomedial support in the hot air balloon technique for treatment of 3-part proximal humeral fractures. J Shoulder Elbow Surg. 2012; 21(9):1152.
716. Park MC, Murthi AM, Roth NS, Blaine TA, Levine WN, Bigliani LU. Two-part and three-part fractures of the proximal humerus treated with suture fixation. J Orthop Trauma. 2003;17(5):319.
717. Parmaksizoglu AS, Sokucu S, Ozkaya U, Kabukcuoglu Y, Gul M. Locking plate fixation of three- and four-part proximal humeral fractures. Acta Orthop Traumatol Turc. 2010;44(2):97.
718. Parnes N, Jupiter JB. Fixed-angle locking plating of displaced proximal humerus fractures. Instr Course Lect. 2010;59:539.
719. Parnes N, Pritsch T, Mozes G. Is surgery the best choice in the treatment of complex fractures of proximal humerus? Preliminary study on 50 cases. JBJS. 2005;87-B(SUPP_III):384.
720. Parsons M, O'Brien RJ, Hughes JS. Locked intramedullary nailing for displaced and unstable proximal humerus fractures. Tech Should Elb Surg. 2005;6(2):75.
721. Paulechova M. Optimalization of rehabilitation programm after fracture of proximal end and diaphysis humeri. Rehabilitacia. 2001; 34(2):77.
722. Pavlopoulos, DA Badras LS, Georgiou CS, Skretas EF, Malizos KN. Hemiarthroplasty for three- and four- part displaced fractures of the proximal humerus in patients over 65 years of age. Acta Orthop Belg. 2007;73(3):306.
723. Pawaskar AC, Lee KW, Kim JM, Park JW, Aminata IW, Jung HJ, Chun JM, Jeon IH. Locking plate for proximal humeral fracture in the elderly population: serial change of neck shaft angle. Clin Orthop Surg. 2012;4(3):209.
724. Peng CH, Wu WT, Yu TC, Chen LC, Hsu SH, Kwong ST, Yao TK, Wu KC, Shao PC, Wang JH, Chen IH.Surgical treatment for proximal humeral fracture in elderly patients with emphasis on the use of intramedullary strut allografts. Tzu Chi Medical Journal. 2012;24(3):131.
725. Perez-Sanz JR, Durudogan H. A method to facilitate open reduction and internal fixation of surgical neck fractures. Orthopedics. 2003;26(6):619.
726. Petit CJ, Millett PJ, Endres NK, Diller D, Harris MB, Warner JJ. Management of proximal humeral fractures: surgeons don't agree. J Shoulder Elbow Surg. 2010;19(3):446.
727. Phipatanakul WP, Norris TR. Indications for prosthetic replacement in proximal humeral fractures. Instr Course Lect. 2005;54:357.
728. Pietu G, Deluzarches P, Gouin F, Letenneur J. Complex injuries of the proximal humerus treated with humeral head prosthesis. Apropos of 21 cases reviewed after a median 4-year follow-up. Acta Orthop Belg. 1992;58(2):159.
729. Pijls BG, Werner PH, Eggen PJ. Primary uncemented hemiarthroplasty for severe fractures of the proximal humerus. J Orthop Trauma. 2011;25(5):279.
730. Pijls BG, Werner PH, Eggen PJ. Alternative humeral tubercle fixation in shoulder hemiarthroplasty for fractures of the proximal humerus. J Shoulder Elbow Surg. 2010;19(2):282.
731. Pilgaard S. Classification of proximal fractures of the humerus (Danish). Ugeskr Laeger. 1974;136(28):1565.
732. Plausinis D, Kwon YW, Zuckerman JD. Complications of humeral head replacement for proximal humeral fractures. Instr Course Lect. 2005;54:371.
733. Plecko M, Kraus A. Internal fixation of proximal humerus fractures using the locking proximal humerus plate. Operative Orthopadie und Traumatologie. 2005;17(1):25.
734. Poeze M, Lenssen AF, Van Empel JM, Verbruggen JP. Conservative management of proximal humeral fractures: Can poor functional outcome be related to standard transscapular radiographic evaluation? J Shoulder Elbow Surg. 2010;19(2):273.
735. Poigenfurst J, Reiler T. Conservative therapy and treatment results in proximal humeral fractures. Hefte Unfallheilkd. 1982;160:123.
736. Pokorny D, Sosba A, Vavrik P, Jahoda D. Shoulder arthroplasty in traumatological indications, long-term results. Acta Chir Orthop Traumatol Cech. 2004;71(5):272.
737. Polendakov D, Tzatchev N, Tzonkov K, Assiov S. Percutaneous fixation of comminuted fractures of the proximal humerus. Ortopediya i Travmatologiya. 1999;35(3):192.
738. Popescu D, Fernandez-Valencia JA, Rios M, Cune J, Domingo A, Prat S. Internal fixation of proximal humerus fractures using the T2-proximal humeral nail. Arch Orthop Trauma Surg. 2009;129(9):1239.
739. Porcellini G, Campi F, Baccarani G, Galassi R. Hemiarthroplasty of the shoulder. Clinical experience in 18 cases treated by the Neer monoblock prosthesis. Chirurgia Degli Organi di Movimento. 1999;84(1):49.
740. Pospula W, Noor TA. Hackethal bundle nailing with intramedullary elastic nails in the treatment of two- and three-part fractures of the proximal humerus: initial experience at Al Razi Hospital, Kuwait. Med Princ Pract. 2009;18(4):284.
741. Prakash U, McGurty DW, Dent JA. Hemiarthroplasty for severe fractures of the proximal humerus. J Shoulder Elbow Surg. 2002;11(5):428.
742. Montana JRP, Ugarte CL, Sastre AL,Garcia FJG, Muniz JAA. Complicated proximal fractures of the humerus, treated with prosthesis. Revista de Ortopedia y Traumatologia. 1997;41(3):252.
743. Prokop A, Skouras E, Jubel A, Helling KJ, Rehm KE. New stable-angle plate systems. Preliminary experience with use of the locking compression plate. Chirurgische Praxis. 2002;60(4):665.
744. Putz P, Arias C, Bremen J, Delvaux D, Simons M. Treatment of epiphyseal fractures of the proximal humerus using Hackethal's bundled wires. Apropos of 136 cases. Acta Orthop Belg. 1987;53(1):80.
745. Qian QR, Wu HS, Li XH, Wu YL, Zhu YL. Artificial shoulder arthroplasty for non-tumor shoulder injury. CRTER. 2007;11(45):9204.
746. Qian QR, Wu HS, Zhou WJ, Li XH, Wu YL. Proximal humeral fractures treated with arthroplasty. Chin J Traumatol. 2005;8(5):283.
747. Qidwai SA. Treatment of proximal humeral fractures by intramedullary Kirschner wires. J Trauma Injury Infect Crit Care. 2001;50(6):1090.
748. Quarz V, Ludin D, Styger S, Wullschleger C, Regazzoni P, Gross T. Proximal humerus fractures in elderly: claim and reality of an anatomical fixation system with angular stability (PHILOS). Br J Surg. 2004;91(7):914.
749. Rader CP, Keller HW, Rehm KE. Operative treatment of three- and four-segment fractures of the proximal humerus. Unfallchirurg. 1992;95(12):613.
750. Raissadat K, Struben PJ, van Loon CJM. Helix wire osteosynthesis for proximal humeral fractures: unacceptable nonunion rate in two- and three-part fractures. Arch Orthop Trauma Surg. 2004;124(3):166.
751. Rajasekhar C, Ray PS, Bhamra MS. Fixation of proximal humeral fractures with the Polarus nail. J Shoulder Elbow Surg. 2001;10(1):7.
752. Rajesh MB. The effect of bone quality on the intramedullary fixation of proximal humeral fractures. Bone. 2003;32(5):S165.
753. Randelli M. Proximal fractures of the humerus: Classification criteria. Minerva Ortop. 1985;36(11):825.
754. Rapala K, Obrebski M. Evaluation of various methods treatment of proximal humerus fractures. Ortopedia Traumatologia Rehabilitacja. 2003;5(1):15.
755. Rasmussen S, Hvass I, Dalsgaard J, Christensen BS, Holstad E. Displaced proximal humeral fractures: results of conservative treatment. Injury. 1992;23(1):41.
756. Heyde RL. Occupational therapy interventions for shoulder conditions: a systematic review. Am J Occup Ther. 2011;65(1):16.
757. Reitman RD, Kerzhner E. Reverse shoulder arthoplasty as treatment for comminuted proximal humeral fractures in elderly patients. Am J Orthop. 2011;40(9):458.
758. Resch H, Povacz P, Frohlich R, Wambacher M. Percutaneous fixation of three- and four-part fractures of the proximal humerus. JBJS (Br). 1997;79(2):295.
759. Ribeyre JP, Rabourdin JP. Rehabilitation following trauma in the elderly. Revue de Medecine. 1981;22(27-28):1775.
760. Ricchetti ET, Warrender WJ, Abboud JA. Use of locking plates in the treatment of proximal humerus fractures. J Shoulder Elbow Surg. 2010;19(2 Suppl):66.
761. Ricchetti ET, DeMola PM, Roman D, Abboud JA. The Use of Precontoured Humeral Locking Plates in the Management of Displaced Proximal Humerus Fracture. J Am Acad Orthop Surg. 2009;17(9):582.
762. Ricci WM. Locked plate fixation for fractures of the proximal humerus. Tech Orthop. 2007;22(4):192.
763. Riedel K, Bischof J. Active functional treatment of proximal fractures of the humerus in surgical ambulatory care. Zeitschrift fur Arztliche Fortbildung . 1986;80(12):491.
764. Rietveld AB, Daanen HA, Rozing PM, Obermann WR. The lever arm in glenohumeral abduction after hemiarthroplasty. JBJS (Br). 1988;70(4):561.
765. Ring D. Current concepts in plate and screw fixation of osteoporotic proximal humerus fractures. Injury Int J Care Inj. 2007;38:59.
766. Roberts VI, Komarasamy B, Pandey R. Modification of the Resch procedure: A new technique and its results in managing three- and four-part proximal humeral fractures. JBJS (Br). 2012;94(10):1409.
767. Robinson BC, Athwal GS. Sanchez-Sotelo J, Rispoli DM. Classification and imaging of proximal humerus fractures. Orthop Clin North Am. 2008;39(4):393.
768. Robinson CM, Christie J. The two-part proximal humeral fracture: a review of operative treatment using two techniques. Injury. 1993;24(2):123.
769. Robinson CM, Inman D, Phillips SA. The plate-joystick technique to reduce proximal humeral fractures and nonunions with a varus deformity through the extended deltoid-splitting approach. J Orthop Trauma. 2011;25(10):634.
770. Robinson CM, Khan L, Akhtar A, Whittaker R. Innovations in practice. The extended deltoid-splitting approach to the proximal humerus. Curr orthop Pract. 2008;19(3):308.
771. Robinson CM, Murray IR. The extended deltoid-splitting approach to the proximal humerus: variations and extensions. JBJS (Br). 2011;93(3):387.
772. Robinson CM, Page RS. Severely impacted valgus proximal humeral fractures. JBJS. 2004;86-A(Suppl 1(Pt 2):143.
773. Robinson CM, Stone OD, Murray IR. Proximal humeral fractures due to blunt trauma producing skin compromise. JBJS (Br). 2011;93B(12):1632.
774. Robinson CM, Wylie JR, Ray AG, Dempster NJ, Olabi B, Seah KTM, Akhtar MA. Proximal humeral fractures with a severe varus deformity treated by fixation with a locking plate. JBJS. 2010;92(5):672.
775. Robinson CM, Amin AK, Godley KC, Murray IR, White TO. Modern Perspectives of Open Reduction and Plate Fixation of Proximal Humerus Fractures. J Orthop Trauma. 2011;25(10):618.
776. Robinson CM, Page RS. Severely impacted valgus proximal humeral fractures - Results of operative treatment. JBJS. 2003;85A(9):1647.
777. obinson CM, Page RS, Hill RMF, Sanders DL, Court-Brown CM, Wakefield AE. Primary hemiarthroplasty for treatment of proximal humeral fractures. JBJS. 2003;85A(7):1215.
778. RÃ¶derer G, Erhardt J, Graf M, Kinzl L, Gebhard F. Clinical results for minimally invasive locked plating of proximal humerus fractures. J Orthop Trauma. 2010;24(7):400.
779. Roderer G, Abouelsoud M, Gebhard F, Bockers TM, Kinzl L. Minimally invasive application of the non-contact-bridging (NCB) plate to the proximal humerus: an anatomical study. J Orthop Trauma. 2007;21(9):621.
780. Roderer G, AbouElsoud M, Gebhard F, Claes L, Aschoff AJ, Kinzl L. Biomechanical investigation of fixed-angle plate osteosynthesis of the proximal humerus. Unfallchirurg. 2010;113(2):133.
781. Roderer G, Erhardt J, Kuster M, Vegt P, Bahrs C, Kinzl L, Gebhard F. Second generation locked plating of proximal humerus fractures--a prospective multicentre observational study. Int Orthop. 2011;35(3):425.
782. Roderer G, Gebhard F, Erhardt J, Al-Agha S, AbouElsoud M, Kinzl L. The Non-Contact Bridging Plate. A new fixed-angle device for the minimally-invasive treatment of fractures of the proximal humerus--technique and preliminary results. Unfallchirurg. 2007;110(6):505.
783. Roderer G, Sperfeld AD, Hansen P, Krischak G, Gebhard F, Kassubek J. Electrophysiological assessment of the deltoid muscle after minimally invasive treatment of proximal humerus fractures - a clinical observation. Open Orthop J. 2011;5:223.
784. Rodop O, Kiral A, Akmaz I, Arpacioglu MO, Sanel S, Kaplan H. Early results of primary hemiarthroplasty for the intraarticular displaced proximal humeral fractures in the early patients. Artroplasti Artroskopik Cerrahi. 2002;13(2):78.
785. Roederer G, Gebhard F, Krischak G, Wilke HJ, Claes L. Biomechanical in vitro assessment of fixed angle plating using a new concept of locking for the treatment of osteoporotic proximal humerus fractures. Int Orthop. 2011;35(4):535.
786. Rogister G, Vanguers I. Treatment of fractures of the proximal 3d of the humerus. Rev Med Liege. 1966;21(6):140.
787. Rojer DE, Yu RS, Barron OA. Blood transfusion in proximal humerus fractures. Am J Orthop. 2002;31(9):513.
788. Rommens PM, Heyvaert G. Conservative treatment of subcapital humerus fractures. A comparative study of the classical Desault bandage and the new Gilchrist bandage. Unfallchirurgie. 1993;19(2):114.
789. Rose PS, Adams CR, Torchia ME, Jacofsky DJ, Haidukewych GG, Steinmann SP. Locking plate fixation for proximal humeral fractures: Initial results with a new implant. J Shoulder Elbow Surg. 2007;16(2):202.
790. Rota A, De Santis P. Transdeltoid approach for plate osteosynthesis with angular stability in proximal humeral fractures. J Orthop Trauma (Conf.). 2011;12:S9.
791. Rothstock S, Plecko M, Kloub M, Schiuma D, Windolf M, Gueorguiev B. Biomechanical evaluation of two intramedullary nailing techniques with different locking options in a three-part fracture proximal humerus model. Clin Biomech. 2012;27(7):686.
792. Roux A, Decroocq L, El Batti S, Bonnevialle N, Moineau G, Trojani C, Boileau P, de Peretti F. Epidemiology of proximal humerus fractures managed in a trauma center. OTSR. 2012;98(6):715.
793. Rowles DJ, McGrory JE. Percutaneous pinning of the proximal part of the humerus. An anatomic study. JBJS. 2011; 83-A(11):1695.
794. Ruch DS, Glisson RR, Marr AW, Russell GB, Nunley JA. Fixation of three-part proximal humeral fractures: a biomechanical evaluation. J Orthop Trauma. 2000;14(1):36.
795. Ruchholtz S, Hauk C, Lewan U, Franz D, Kuhne C, Zettl R. Minimally invasive polyaxial locking plate fixation of proximal humeral fractures: a prospective study. J Trauma Injury Infect Crit Care. 2011;71(6):1737.
796. Rueger JM, Ruecker A, Briem D, Grossterlinden L, Linhart W. Proximal humeral fractures: Nailing. Eur J Trauma Emerg Surg. 2007;33(4):357.
797. Russo R, Cautiero F, Della Rotonda G. The classification of complex 4-part humeral fractures revisited: the missing fifth fragment and indications for surgery. Musculoskelet Surg. 2012;96(Suppl 1):S13.
798. Russo R, Cautiero F, Lombardi LV, Visconti V. Telegraph antegrade nailing in the treatment of humeral fractures with rotator interval split technique. Chirurgia Degli Organi di Movimento. 2009;93(Suppl 1):S7.
799. Russo R, Lombardi LV, Ciccarelli M, Giudice G, Cautiero F. A new osteosynthesis device for the treatment of proximal humerus fractures. Description of the technique and preliminary results. Chirurgia Degli Organi di Movimento. 2008;91(1):27.
800. Russo R, Lombardi LV, Cautiero F, Giudice G, Ciccarelli M. Medial reconstruction technique in the treatment of complex fractures of humeral proximal epiphysis with SMR prosthetic modular system. Chirurgia Degli Organi di Movimento. 2008;91(2):117.
801. Russo R, Visconti V, Lombardi LV, Ciccarelli M, Cautiero F. Da Vinci System: clinical experience with complex proximal humerus fractures. Musculoskelet Surg. 2010;94(Suppl 1):S57.
802. Russo R, Visconti V, Lombardi LV, Ciccarelli M, Giudice G. The block-bridge system: A new concept and surgical technique to reconstruct articular surfaces and tuberosities in complex proximal humeral fractures. J Shoulder Elbow Surg. 2008;17(1):29.
803. Rutten MJ, Jager GJ, de Waal Malefijt MC, Blickman JG. Double line sign: a helpful sonographic sign to detect occult fractures of the proximal humerus. Eur Radiol. 2007;17(3):762.
804. Sadowski C, Riand N, Stern R, Hoffmeyer P. Fixation of fractures of the proximal humerus with the PlantTan Humerus Fixator Plate: early experience with a new implant. J Shoulder Elbow Surg. 2003;12(2):148.
805. Sahu RL. Philos Locking plates in proximal humerus fractures -- literature review. Internet J Health. 2010;11(1):8p.
806. Saitoh S, Nakatsuchi Y. Osteoporosis of the proximal humerus: Comparison of bone-mineral density and mechanical strength with the proximal femur. J Shoulder Elbow Surg. 1993;2(2):78.
807. Saitoh S, Nakatsuchi Y, Latta L, Milne E. Distribution of bone mineral density and bone strength of the proximal humerus. J Shoulder Elbow Surg. 1994;3(4):234.
808. Sallay PI, Pedowitz RA, Mallon WJ, Vandemark RM, Dalton JD, Speer KP. Reliability and reproducibility of radiographic interpretation of proximal humeral fracture pathoanatomy. J Shoulder Elbow Surg. 1997;6(1):60.
809. Monzo CS, Velazco OV, Guillen DVC, Sancho FG. Risk Factors for Proximal Humerus Fractures. Osteoporosis Int. 2010;21:275.
810. Sanders BS, Bullington AB, McGillivary GR, Hutton WC. Biomechanical evaluation of locked plating in proximal humeral fractures. J Shoulder Elbow Surg. 2007;16(2):229.
811. Sanders RJ, Thissen LG, Teepen JC, van Kampen A, Jaarsma RL. Locking plate versus nonsurgical treatment for proximal humeral fractures: better midterm outcome with nonsurgical treatment. J Shoulder Elbow Surg. 2011;20(7):1118.
812. Reig JS, Tufanisco CB, Utrilla AL, Gomez RC. Percutaneous fixation of two and three part displaced fractures of the proximal humerus. Revista de Ortopedia y Traumatologia. 2003; 47(1):26.
813. Saran N, Bergeron SG, Benoit B, Reindl R, Harvey EJ, Berry GK. Risk of axillary nerve injury during percutaneous proximal humerus locking plate insertion using an external aiming guide. Injury. 2010;41(10):1037.
814. Sartori E, Fusi M, Gaudenzi A, Cantalamessa G. Long-term results of conservatively treated fractures of the upper end of the humerus. Archivio Putti di Chirurgia Degli Organi di Movimento. 1989;37(2):389.
815. Savarese A, Bellini C, Cossandi GC, Bonetti L, Pasquali F. Role of the rotator cuff anatomic reconstruction in the prosthesis of proximal humeral fractures. Minerva Ortopedica e Traumatologica. 2005;56(5):411.
816. Savoie FH, Geissler WB, Vander Griend RA. Open reduction and internal fixation of three-part fractures of the proximal humerus. Orthopedics. 1989;12(1):65.
817. Schai P, Imhoff A, Staubli AE. Differential diagnosis and therapy of multi-fragment humeral head fracture--an analysis of three clinical studies. Zeitschrift fur Unfallchirurgie und Versicherungsmedizin. 1993;86(1):27.
818. Schai P, von Flue M, Staubli AE, Vogt B. Functional results of Neer II type hemi-arthroplasty joint replacement of multi-fragment humerus head fractures. Helv Chir Acta. 1991;57(5):799.
819. Schai PA, Hintermann B, Koris MJ. Preoperative arthroscopic assessment of fractures about the shoulder. Arthroscopy. 1999;15(8):827.
820. Scheibel M. Humeral head fracture and cuff. Unfallchirurg. 2011;114(12):1075.
821. Schiffern SC, Krishnan SG, Burkhead JR. Percutaneous Pinning for Surgical Neck Fracture: Method of Choice-Opposes. Semin Arthroplasty. 2007;18(1):23.
822. Schittko A, Braun W, Ruter A. Experiences with the ortra-prosthesis in primary prosthetic replacement of fractures of the humeral head - Indication, technique and results. Zentralbl Chir. 2003;128(1):12.
823. Schittko A, Ruter A. Proximal humeral fracture in the elderly. Primary head replacement as one alternative. Chirurg. 2003;74(11):990.
824. Schlachetzki J. Functional treatment of proximal upper arm fractures] Chirurg. 1966;37(7):297.
825. Schliemann B, Siemoneit J, Theisen CH, Kosters C, Weimann A, Raschke MJ. Complex fractures of the proximal humerus in the elderly--outcome and complications after locking plate fixation. Musculoskelet Surg. 2012;96(Suppl 1):S3.
826. Schmal H, Klemt C, Sudkamp NP. Evaluation of shoulder arthroplasty in treatment of four-fragment fractures of the proximal humerus. Unfallchirurg. 2004;107(7):575.
827. Schmelzeisen H, Furche A. Effect of the rotation cuff on proximal and distal humeral fractures. Hefte Unfallheilkd. 1975;126:35.
828. Schmidt J, Calmez D, Bak P. Comparison of different methods of osteosynthesis from proximal humerus fractures and there treatment. Physik Med Rehabilitationsmed Kurort. 2005;15(2):99.
829. Schnabel M, Bahrs C, Walthers E, Gotzen L. Marburg shoulder radiography splint (MSR splint) for standardized and high quality plain film radiography in fractures of the proximal humerus. Unfallchirurg. 2004;107(11):1099.
830. Schober HC, Basgen K, Rattey V, Wiedbusch J, Haar P, Westphal T, Mittlmeier T. Rate of proximal humerus fractures in a defined urban population. Osteoporosis Int (Conf.). 2011;22:S351.
831. Schulte LM, Matteini LE, Neviaser RJ. Proximal periarticular locking plates in proximal humeral fractures: functional outcomes. J Shoulder Elbow Surg. 2011;20(8):1234.
832. Schumer RA, Muckley KL, Markert RJ, Prayson MJ, Heflin J, Konstantakos EK, Goswami T. Biomechanical comparison of a proximal humeral locking plate using two methods of head fixation. J Shoulder Elbow Surg. 2010;19(4):495.
833. Schwartz C, Cuny C. Fractures of the proximal humerus: A prospective review of 188 cases. Eur J Orthop Surg Traumatol. 2003;13(1):1.
834. Schwarz N, Hocker K, Jung I. The measurements of shoulder mobility - Is there still a place for the SFTR-method? Acta Chir Austriaca. 2000;32(1):31.
835. Seemann WR, Siebler G, Rupp HG. A new classification of proximal humeral fractures. Eur J Radiol. 1986;6(3):163.
836. Sehr JR, Szabo RM. Semitubular blade plate for fixation in the proximal humerus. J Orthop Trauma. 1988;2(4):327.
837. Seide K, Triebe J, Faschingbauer M, Schulz AP, Puschel K, Mehrtens G, Jurgens CH. Locked vs. unlocked plate osteosynthesis of the proximal humerus -- a biomechanical study. Clin Biomech. 2007;22(2):176.
838. Serin E, Karatosun V, Balci C, Koseoglu HC, Ersoy HH. Two-prong splint in the treatment of proximal humeral fracture. Arch Orthop Trauma Surg. 1999;119(7-8):368.
839. Iammarrone CS, Lotito FM, Carotenuto F. Treatment of displaced fractures of the proximal third of the humerus based on our personal experience. Chirurgia Degli Organi di Movimento. 1986;71(4):355.
840. Seyhan M, Kocaoglu B, Nalbantoglu U, Aydin N, Guven O. Technique of Kirschner wire reduction and fixation of displaced two-part valgus angulated proximal humerus fractures at the surgical neck. J Orthop Trauma. 2012;26(6):e46.
841. Seyr V, Heel S, Dunser M. Intramedullary claw for stabilization of proximal humerus fractures: a comparison with other semirigid techniques. Chirurg. 2010;81(8):728.
842. Sforzo CR, Wright TW. Treatment of acute proximal humerus fractures with a polarus nail. J Surg Orthop Adv. 2009;18(1):28.
843. Shabat S, Gepstein R, Mann G, Kish B, Stern A, Fredman B, Nyska M. Does the combination of a simultaneous subcapital fracture of humerus and hip fracture in elderly patients carry a prognostic value?. Arch Gerontol Geriatr. 2002;35(1):71.
844. Shah N, Iqbal HJ, Brookes-Fazakerley S, Sinopidis C. Shoulder hemiarthroplasty for the treatment of three and four part fractures of the proximal humerus using Comprehensive[REGISTERED] Fracture stem. Int Orthop. 2011;35(6):861.
845. Shahid R, Mushtaq A, Northover J, Maqsood M. Outcome of proximal humerus fractures treated by PHILOS plate internal fixation. Experience of a district general hospital. Acta Orthop Belg. 2008;74(5):602.
846. Sharafeldin, KN, Quinlan JF, Corrigan J, Kelly IP. Functional follow-up of locking plate fixation of fractures of the proximal humerus. Eur J Orthop Surg Traumatol. 2008;18(2):87.
847. Shen SM, Mao BY, Wang C, Huang T, Jia XW, Gao LS, Chen YJ. [Replacement of humeral head prosthesis for four-part proximal humeral fractures or fracture-dislocations. Zhongguo Gushang. 2008;21(5):387.
848. Sheng ZJ, Ma YH, Tian SQ, Gu JY. Comparative study on the clinical results of locking proximal humerus plate (LPHP) and traditional plates in the treatment of proximal humerus fractures in the young and middle-aged patients. Zhongguo Gushang. 2008;21(9):684.
849. Shi HF, Xiong J, Chen YX, Wang JF, Wang SF, Chen ZJ, Qiu Y. Management of proximal humeral fractures in elderly patients with uni- or polyaxial locking osteosynthesis system. Arch Orthop Trauma Surg. 2011;131(4):541.
850. Shrader MW, Sanchez-Sotelo L, Sperling JW, Rowland CM, Cofield RH. Understanding proximal humerus fractures: Image analysis, classification, and treatment. J Shoulder Elbow Surg. 2005;14(5):497.
851. Shved SI, Sysenko IM. Treatment of fractures of the proximal end of the humerus in middle-aged and elderly patients by the Ilizarov method. Vestnik khirurgii imeni I.I.Grekova. 1984;132(5):80.
852. Sidor ML, Zuckerman JD, Lyon T, Koval K, Cuomo F, Schoenberg N. The Neer classification system for proximal humeral fractures. An assessment of interobserver reliability and intraobserver reproducibility. JBJS. 1993;75(12):1745.
853. Sidor ML, Zuckerman JD, Lyon T, Koval K, Schoenberg N. Classification of proximal humerus fractures: The contribution of the scapular lateral and axillary radiographs. J Shoulder Elbow Surg. 1994;3(1):24.
854. Siebenrock KA, Gerber C. The reproducibility of classification of fractures of the proximal end of the humerus. JBJS. 1993;75(12):1751.
855. Siebenrock KA, Gerber C. Classification of fractures and problems in proximal humeral fractures. Orthopade. 1992;21(2):98.
856. Siebler G, Kuner EH. Late results following the surgical treatment of proximal humerus fractures in adults. Unfallchirurgie. 1985;11(3):119.
857. Siebler G, Walz H, Kuner EH. Minimal osteosynthesis of fractures of the head of the humerus. Indications, technic, results. Unfallchirurg. 1989;92(4):169.
858. Siffri PC, Peindl RD, Coley ER, Norton J, Connor PM, Kellam JE. Biomechanical analysis of blade plate versus locking plate fixation for a proximal humerus fracture: Comparison using cadaveric and synthetic humeri. J Orthop Trauma. 2006;20(8):547.
859. Simon JA, Puopolo SM, Capla EL, Egol KA, Zuckerman JD, Koval KJ. Accuracy of the axillary projection to determine fracture angulation of the proximal humerus. Orthopedics. 2004;27(2):205.
860. Sinha S, Kelly CP. Fixed angle locking plates for proximal humeral fracture fixation. Ann R Coll Surg Engl. 2010;92(8):631.
861. Sirveaux F, Roche O, Mole D. Shoulder arthroplasty for acute proximal humerus fracture. Orthop Traumatol Surg Res. 2010;96(6):683.
862. Siuni E, Planta M, Orgiano F, Pusceddu I, Orru B. The treatment of proximal humeral fractures with antegrade locked intramedullary nail. J Orthop Trauma (Conf.) 2011;12:S7.
863. Siwach R, Singh R, Rohilla RK, Kadian VS, Sangwan SS, Dhanda M. Internal fixation of proximal humeral fractures with locking proximal humeral plate (LPHP) in elderly patients with osteoporosis. J Orthop Surg Traumatol. 2008; 9(3):149
864. Sjoden GOJ, Movin T, Guntner P, Aspelin P, Ahrengart L, Ersmark H, Sperber A. Poor reproducibility of classification of proximal humeral fractures. Additional CT of minor value. Acta Orthop Scand. 1997; 68(3):239
865. Sjoden GOJ, Movin T, Aspelin P, Guntner P, Shalabi A. 3D-radiographic analysis does not improve the Neer and AO classifications of proximal humeral fractures. Acta Orthop Scand. 1999; 70(4):325
866. Skutek M, Fremerey RW, Bosch U. Level of physical activity in elderly patients after hemiarthroplasty for three- and four-part fractures of the proximal humerus. Arch Orthop Traum Su. 1998; 117(4-5):252
867. Slobogean GP, Noonan VK, Famuyide A, O'Brien PJ. Does objective shoulder impairment explain patient-reported functional outcome? A study of proximal humerus fractures. J Shoulder Elbow Surg. 2011; 20(2):267
868. Slobogean GP, Noonan VK, O'Brien PJ. The reliability and validity of the Disabilities of Arm, Shoulder, and Hand, EuroQol-5D, Health Utilities Index, and Short Form-6D outcome instruments in patients with proximal humeral fractures. J Shoulder Elbow Surg. 2010; 19(3):342
869. Smejkal K, Dedek T, Zvak I, Trlica J, Folvarsky J, Pocepcov I, Zahradnicek J, Holecek T, Koci J, Frank M. Surgical management of proximal humeral fractures using the Hackethal (Zifko) method--a case review. Rozhl Chir. 2008; 87(2):101
870. Smejkal K, Didek T, Zvak I, Trlica J, Folvarsky J. Operation treatment of 2-3 fragments proximal humeral fractures using Zifko versus Philos. Eur J Trauma Emerg Surg. 2008; 34(Suppl.1):44
871. Smejkal K, Lochman P, Dedek T, Trlica J, Koci J, Zvak I. Surgical Treatment for Proximal Humerus Fracture. Acta Chir Orthop Traumatol Cech. 2011; 78(4):321
872. Smith AM, Mardones RM, Sperling JW, Cofield RH. Early complications of operatively treated proximal humeral fractures. J Shoulder Elbow Surg. 2007; 16(1):14
873. Smith AM, Sperling JW, Cofield RH. Complications of operative fixation of proximal humeral fractures in patients with rheumatoid arthritis. J Shoulder Elbow Surg. 2005; 14(6):559
874. Smith J, Berry G, Laflamme Y, Blain-Pare E, Reindl R, Harvey E. Percutaneous insertion of a proximal humeral locking plate: an anatomic study. Injury. 2007; 38(2):206
875. Smith M, Jacobs L, Banks L, Warner J, Sylvester B, Bale R. Internal fixation of fractures of the proximal humerus with a humeral fixator plate (PlantTan Plate): a two year follow-up. Acta Orthop Belg. 2008; 74(6):735
876. Soete PJ, Clayson PE, Costenoble VH. Transitory percutaneous pinning in fractures of the proximal humerus. J Shoulder Elbow Surg. 1999; 8(6):569
877. Solberg BD, Moon CN, Franco DP, Paiement GD. Locked plating of 3- and 4-part proximal humerus fractures in older patients: the effect of initial fracture pattern on outcome. J Orthop Trauma. 2009; 23(2):113
878. Solberg BD, Moon CN, Franco DP, Paiement GD. Surgical Treatment of Three and Four-Part Proximal Humeral Fractures. J Bone Joint Surg Am Vol. 2009; 91A(7):1689
879. Solem-Bertoft E, Lundh I, Westerberg CE. Pain is a major determinant of impaired performance in standardized active motor tests. A study in patients with fracture of the proximal humerus. Scand J Rehabil Med. 1996; 28(2):71
880. Sonderegger J, Simmen HP. Epidemiology, treatment and results of proximal humeral fractures: experience of a district hospital in a sports- and tourism area. Zentralbl Chir. 2003; 128(2):119
881. Sosef N, van Leerdam R, Ott P, Meylaerts S, Rhemrev S. Minimal invasive fixation of proximal humeral fractures with an intramedullary nail: good results in elderly patients. Arch Orthop Trauma Su. 2010; 130(5):605
882. Sosef N, Stobbe I, Hogervorst M, Mommers L, Verbruggen J, van der Elst M, Rhemrev S. The Polarus intramedullary nail for proximal humeral fractures - Outcome in 28 patients followed for 1 year. Acta Orthop. 2007; 78(3):436
883. Sosna A, Cech O. The reconstruction of fragmented fractures of the upper humerus. A personal surgical technique. Acta Chir Orthop Traumatol Cech. 1979; 46(3):248
884. Sosna A, Fric V. Endoprosthesis of the shoulder joint designed by the authors - surgical technique. Acta Chir Orthop Traumatol Cech. 1996; 63(5):261
885. Sosna A, Fric V. Results of reconstructive surgery in fractures of the proximal humerus. Acta Chir Orthop Traumatol Cech. 1993; 60(4):209
886. Sosna A, Pokorny D, Hromadka R, Jahoda D, Bartak V, Pinskerova V. A new technique for reconstruction of the proximal humerus after three- and four-part fractures. J Bone Joint Surg Br. 2008; 90(2):194
887. Sosna A, Pokorny D, Vavrik P, Jahoda D. Shoulder arthroplasty in traumatological indications, surgical technique. Acta Chir Orthop Traumatol Cech. 2004; 71(5):265
888. Speck M, Lang FJ, Regazzoni P. Proximal humeral multiple fragment fractures--failures after T-plate osteosynthesis. Swiss Surgery. 1996; (2):51
889. Speck M, Regazzoni P. 4-fragment fractures of the proximal humerus. Alternative strategies for surgical treatment. Unfallchirurg. 1997; 100(5):349
890. Sperling JW, Cuomo F, Hill JD, Hertel R, Chuinard C, Boileau P. The difficult proximal humerus fracture: tips and techniques to avoid complications and improve results. Instr Course Lect. 2007; 56:45
891. Spina N, Mus L, Basile G. Intramedullary bundle wiring in the humeral proximal fractures. Tecnique details. Minerva Ortop Traumatol. 1997; 48(9):349
892. Spross C, Platz A, Rufibach K, Lattmann T, Forberger J, Dietrich M. The PHILOS plate for proximal humeral fractures-Risk factors for complications at one year. J Trauma Acute Care Surg. 2012; 72(3):783
893. Spross C, Platz A, Erschbamer M, Lattmann T, Dietrich M. Surgical Treatment of Neer Group VI Proximal Humeral Fractures Retrospective Comparison of PHILOS (R) and Hemiarthroplasty. Clin.Orthop. 2012; 470(7):2035
894. Sproul RC, Iyengar JJ, Devcic Z, Feeley BT. A systematic review of locking plate fixation of proximal humerus fractures. Injury. 2011; 42(4):408
895. Sram J, Lukas, R Krivohlavek M, Taller S. Application of the Targon PH long nail in storey fractures and metaphyseal fractures of the proximal humerus. Rozhl Chir. 2007; 86(5):254
896. Stecco C, Gagliano G, Lancerotto L, Tiengo C, Macchi V, Porzionato A, De Caro R, Aldegheri R. Surgical anatomy of the axillary nerve and its implication in the transdeltoid approaches to the shoulder. J Shoulder Elbow Surg. 2010; 19(8):1166
897. Stedtfeld HW, Attmanspacher W, Thaler K, Frosch B. Fixation of humeral head fractures with antegrade intramedullary nailing. Zentralbl Chir. 2003; 128(1):6
898. Stedtfeld HW, Morlock M, Sellenschloh K, Puschel K, Mittlmeier T, Gradl G. Locking plate fixation of humeral head fractures with a telescoping screw. A comparative biomechanical study versus a standard plate. Injury. 2012; 43(6):734
899. Stedtfeld HW, Mittlmeier T. Fixation of proximal humeral fractures with an intramedullary nail: Tipps and tricks. Eur J Trauma Emerg Surg. 2007; 33(4):367
900. Stein J, Galatz LM. Reverse Shoulder Replacement: Forward Thinking in Acute Fracture-Affirms. Semin Arthroplasty. 2008; 19(1):2
901. Stephanie A, Kecht M, Oberleitner G, Fialka CH, Vecsei V. Non-surgical treatment of three- and four-part proximal humeral fractures in elderly patients. Scripta Medica Facultatis Medicae Universitatis Brunensis Masarykianae. 2010; 83(2):169
902. Stevanovic M, Slavkovic S, Banovic D, Stojsavljevic J, Apostolovic M, Basic V, Dokic R, Krneta O. Dislocated fractures of the proximal humerus. Acta Orthopaedica Iugoslavica. 1997; 28(2):149
903. Strohm PC, Helwig P, Konrad G, Suedkamp NP. Locking plates in proximal humerus fractures. Acta Chir Orthop Traumatol Cech. 2007; 74(6):410
904. Strohm PC, Kostler W, Sudkamp NP. Locking plate fixation of proximal humerus fractures. Tech Shoulder Elb Surg. 2005; 6(1):8
905. Sturzenegger M, Fornaro E, Jakob RP. Results of surgical treatment of multifragmented fractures of the humeral head. Arch Orthop Trauma Surg. 1982; 100(4):249
906. SuarezSuarez M, FernandezVillian M, VarelaEgocheaga JR, GonzalezSastre V, MurciaMazon A. Internal fixation of proximal humeral fractures using two different locking plates Eur J Trauma Emerg Surg. 2008; 34(Suppl.1):134
907. Sudkamp NP, Bayer J, Hepp R, Voigt C, Oestern H, KÃ¤Ã¤b M, Luo C, Plecko M, Wendt K, KÃ¶stler W, Konrad G. Open reduction and internal fixation of proximal humeral fractures with use of the locking proximal humerus plate. Results of a prospective, multicenter, observational study. J Bone Joint Surg Am. 2009; 1(6):1320
908. Sudkamp NP, Audige L, Lambert S, Hertel R, Konrad G. Path analysis of factors for functional outcome at one year in 463 proximal humeral fractures. J Shoulder Elbow Surg. 2011; 20(8):1207
909. Sudkamp NP, Strohm PC. Locking plates for fractures of the proximal humerus. Aktuelle Traumatol. 2005; 35(3):113
910. Svend-Hansen H. Displaced proximal humeral fractures. A review of 49 patients. Acta Orthop Scand. 1974; 45(3):359
911. Szyszkowitz P, Seggl W, Schleifer P, Cundy PJ. Proximal humeral fractures. Management techniques and expected results Clin Orthop Relat Res. 1993;( 292):13
912. Tabak AY, Aktekin C, Ucaner A, Tasbas BA, Bicimoglu A, Gunel U. Hemiarthroplasty in poximal humeral fractures. Artroplasti Artroskopik Cerrahi. 2001; 12(1):1
913. Takase K.Therapeutic results of the proximal humeral neck fractures. Clinical Calcium. 2003; 13(10):1317
914. Takeuchi R, Koshino T, Nakazawa A, Numazaki S, Sato R, Saito T. Minimally invasive fixation for unstable two-part proximal humeral fractures: Surgical techniques and clinical results using J-nails. J Orthop Trauma. 2002; 16(6):403
915. Taller S, Krivohlavek M, Lukas R, Sram I, Kral M. Hemiarthroplasty for management of proximal humeral fractures. Acta Chir Orthop Traumatol Cech. 2007; 74(4):262
916. Tamai K, Hamada J, Ohno W, Saotome K. Surgical anatomy of multipart fractures of the proximal humerus. J Shoulder Elbow Surg. 2002; 11(5):421
917. Tamai K, Ohno W, Takemura M, Mashitori H, Hamada J, Saotome K. Treatment of proximal humeral fractures with a new intramedullary nail. J Orthop Sci. 2005; 10(2):180
918. Tamai K, Ishige N, Kuroda S, Ohno W,Itoh H, Hashiguchi H, Iizawa N, Mikasa M. Four-segment classification of proximal humeral fractures revisited: A multicenter study on 509 cases. J Shoulder Elbow Surg. 2009; 18(6):845
919. Tan ZY, Zhang SC, Hu WK, Huang CS, Zhu HW. Factors affecting shoulder joint functions following artificial humeral head replacement: 22 cases analysis. Journal of Clinical Rehabilitative Tissue Engineering Research. 2009; 13(48):9550
920. Tanner MW, Cofield RH. Prosthetic arthroplasty for fractures and fracture-dislocations of the proximal humerus. Clin Orthop Relat Res. 1983; (179):116
921. Telwani NC, Liporace F, Walsh M, France MA, Zuckerman JD, Egol KA. Functional outcome following one-part proximal humeral fractures: A prospective study. J Shoulder Elbow Surg. 2008; 17(2):216
922. Terragnoli F, Zattoni G, Damiani L, Cabrioli A, Bassi G. Treatment of proximal humeral fractures with reverse prostheses in elderly patients. J Orthop Trauma. 2007; 8(2):71
923. Thalhammer G, Platzer P, Oberleitner G, Fialka C, Greitbauer M, Vecsei V. Angular Stable Fixation of Proximal Humeral Fractures. J Trauma-Injury Infect Crit Care. 2009; 66(1):204
924. Thanasas C, Kontakis G, Angoules A, Limb D, Giannoudis P. Treatment of proximal humerus fractures with locking plates: a systematic review. J Shoulder Elbow Surg. 2009; 18(6):837
925. Thomazeau H, Richou J, Benkalfalte T, Kerhousse G, Le Nen D, Veillard D. Is it worth fixing proximal humeral fractures at increased vascular risk? Orthop Traumatol Surg Res. 2012; 98(4):383
926. Thyagarajan DS, Haridas SJ, Jones D, Dent C, Evans R, Williams R. Functional outcome following proximal humeral interlocking system plating for displaced proximal humeral fractures. Int J Shoulder Surg. 2009; 3(3):57
927. Tingart M, Bathis H, Lefering R, Bouillon B, Tiling T. Constant Score and Neer Score. A comparison of score results and subjective patient satisfaction. Unfallchirurg. 2001; 104(11):1048
928. Tingart MJ, Lehtinen J, Zurakowski D, Warner JJP, Apreleva M. Proximal humeral fractures: Regional differences in bone mineral density of the humeral head affect the fixation strength of cancellous screws. J Shoulder Elbow Surg. 2006; 15(5):620
929. Torchia ME. Technical tips for fixation of proximal humeral fractures in elderly patients. Instr Course Lect. 2010; 59:553
930. Tornkvist H, Ahrengart L. Tension band wiring vs non-operative treatment of displaced proximal humerus fractures. A prospective randomized study [Abstract] Orthopaedic Transactions. 1997; 21(2):592
931. Torrens C, Corrales M, Melendo E, Solano A, Rodriguez-Baeza A, Caceres E. The pectoralis major tendon as a reference for restoring humeral length and retroversion with hemiarthroplasty for fracture. J Shoulder Elbow Surg. 2008; 17(6):947
932. Torrens C, Corrales M, Vila G, Santana F, Caceres E. Functional and Quality-of-Life Results of Displaced and Nondisplaced Proximal Humeral Fractures Treated Conservatively. J Orthop Trauma. 2011; 25(10):581
933. Towfigh H, Buhl W, Obertacke U. Treatment results after conservative and surgical treatment of proximal upper arm fractures. Aktuelle Traumatol. 1993; 23(8):354
934. Trappey G, Edwards T. Reverse Total Shoulder for Four-Part Proximal Humeral Fractures. Oper Techniq Orthop. 2011; 21(1):79
935. Trentani L, Mazzucco A, Rossi S, Gadaleta MA, Merlo M. Osteosynthesis of fractures of the proximal humerus plate with angular stability and mini-invasive technique: A review of our series. J Orthop Trauma (Conf). 2011; 12:S20
936. Trepat AD, Popescu D, Fernandez-Valencia JA, Cune J, Rios M, Prat S. Comparative study between locking plates versus proximal humeral nail for the treatment of 2-part proximal humeral fractures. Eur J Orthop Surg Traumatol. 2012; 22(5):373
937. Trupka A, Wiedemann E, Ruchholtz S, Brunner U, Habermeyer P, Schweiberer L. Displaced three- and four-part fractures of the humeral head. Does the dislocation of the articular fragment influence outcome? Unfallchirurg. 1997; 100(2):105
938. Twardosz W, Siwinski D. Surgical treatment for fracture of the proximal humerus. Chir Narzadow Ruchu Ortop Pol. 1996; 61(4):357
939. Unger S, Erhart S, Kralinger F, Blauth M, Schmoelz W. The effect of in situ augmentation on implant anchorage in proximal humeral head fractures. Injury. 2012; 43(10):1759
940. Urgelli S, Crainz E, Maniscalco P. Conservative treatment vs prosthetic replacement surgery to treat 3- and 4-fragment fractures of the proximal epiphysis of humerus in the elderly patient. Chirurgia Degli Organi di Movimento. 2005; 90(4):345
941. Valenti P, Katz D, Kilinc A, Elkholti K, Gasiunas V. Mid-term outcome of reverse shoulder prostheses in complex proximal humeral fractures. Acta Orthop Belg. 2012; 78(4):442
942. Valentini R, Martinelli B. The "Helix Wire": our experience in the treatment of humeral neck fractures. Chirurgia Degli Organi di Movimento. 2009; 93(Suppl 1):S15
943. van de Water AT, Shields N, Taylor NF. Outcome measures in the management of proximal humeral fractures: a systematic review of their use and psychometric properties J Shoulder Elbow Surg. 2011; 20(2):333
944. van den Broek CM, van den Besselaar M, Coenen JMF, Vegt PA. Displaced proximal humeral fractures: intramedullary nailing versus conservative treatment. Arch Orthop Trauma Surg. 2007; 127(6):459
945. Van Seymortier P, Stoffelen D, Fortems Y, Reynders R. The reverse shoulder prosthesis (Delta III) in acute shoulder fractures: technical considerations with respect to stability. Acta Orthop Belg. 2006; 72(4):474
946. Verbeek PA, van den Akker-Scheek I, Wendt KW, Diercks RL. Hemiarthroplasty versus angle-stable locking compression plate osteosynthesis in the treatment of three- and four-part fractures of the proximal humerus in the elderly: design of a randomized controlled trial. BMC Musculoskeletal Disorders. 2012; 13:16
947. Garcia EV, Canovas CT, Camps XC, Navarro MM. Review of the surgical treatment of proximal fractures of the humerus by transosseous suture. Revista de Ortopedia y Traumatologia. 2001; 45(5):398
948. Vespasiani A. Percutaneous reduction and fixation of fractures--dislocations and epiphysiolyses of the proximal end of the humerus. Arch Ortop. 1968; 81(2):89
949. Vichard PH, Bellanger P, Laurain JM. Ascending bipolar pinning with elastic pin for unimpacted fractures of the proximal end of the humerus. Chirurgie - Memoires de l. 1982; 108(5):381
950. Villiger KJ. Treatment of proximal humeral fractures using the fork-splint. Chirurg. 1971; 42(11):523
951. Visna P, Beitl E, Jaganjac E, Kalvach J, Smidl Z. Treatment of proximal humeral fractures by angular stabile, antegrade, interlocking intramedullar nail. Rozhl Chir. 2007; 86(4):194
952. Visser CP, Coene LN, Brand R, Tavy DL. Nerve lesions in proximal humeral fractures. J Shoulder Elbow Surg. 2001; 10(5):421
953. Visser CP, Tavy DL, Coene LN, Brand R. Electromyographic findings in shoulder dislocations and fractures of the proximal humerus: comparison with clinical neurological examination. Clin Neurol Neurosurg. 1999; 101(2):86
954. Vogel G, Chechick A, Perry MP, Brosh T. Fixation of humeral surgical neck fracture using contoured pins versus straight pins: a mechanical study. Int Orthop. 2007; 31(6):811
955. Voigt C, Boehme S, Katthagen C, Lill H. Comparison of Results after Primary and Secondary Shoulder Arthroplasty for Proximal Humeral Fractures. Z Orthop Unfallchir. 2012; 150(2):156
956. Voigt C, Bohme S, Katthagen C, Lill H. Comparison of results after primary and secondary shoulder arthroplasty for proximal humeral fractures. Zeitschrift fur Orthopadie & Unfallchirurgie. 2012; 150(2):156
957. Voigt C, Ewig M, Vosshenrich R, Lill H. Value of MRI in preoperative diagnostics of proximal humeral fractures compared to CT and conventional radiography. Unfallchirurg. 2010; 113(5):378
958. Voigt C, Geisler A, Hepp P, Schulz AP, Lill H. Are polyaxially locked screws advantageous in the plate osteosynthesis of proximal humeral fractures in the elderly? A prospective randomized clinical observational study. J Orthop Trauma. 2011; 25(10):596
959. Voigt C, Hurschler C, Althainz J, Vosshenrich R, Lill H. Effect of additive fiber cerclage of the rotator cuff in angle-stable plate-stabilized proximal humeral fractures. A biomechanical study of human shoulder specimens. Unfallchirurg. 2008; 111(7):514
960. Voigt C, Hurschler C, Rechi L, Vosshenrich R, Lill H. Additive fiber-cerclages in proximal humeral fractures stabilized by locking plates: no effect on fracture stabilization and rotator cuff function in human shoulder specimens. Acta Orthopaedica. 2009; 80(4):465
961. Voigt C, Kreienborg S, Megatli O, Schulz AP, Lill H, Hurschler C. How does a varus deformity of the humeral head affect elevation forces and shoulder function? A biomechanical study with human shoulder specimens. J Orthop Trauma. 2011; 25(7):399
962. Voigt C, Lill H. Primary hemiarthroplasty in proximal humerus fractures. Orthopade. 2007; 36(11):1002
963. Voos JE, Dines JS, Dines DM. Arthroplasty for fractures of the proximal part of the humerus. Instr Course Lect. 2011; 60:105
964. Vrancken Peeters MJTFD, Kastelein GW, Breslau PJ. Proximal humerus fractures: A prospective study of the functional outcome after conservative treatment. Eur J Trauma. 2001; 27(3):133
965. Vundelinckx BJ, Dierickx CA, Bruckers L, Dierickx CH. Functional and radiographic medium-term outcome evaluation of the Humerus Block, a minimally invasive operative technique for proximal humeral fractures. J Shoulder Elbow Surg. 2012; 21(9):1197
966. Wachtl SW, Marti CB, Hoogewoud HM, Jakob RP, Gautier E. Treatment of proximal humerus fracture using multiple intramedullary flexible nails. Arch Orthop Trama Surg. 2000; 120(3-4):171
967. Walch G, Boileau P, Noel E. Shoulder arthroplasty: evolving techniques and indications. Joint, Bone, Spine: Revue du Rhumatisme. 2010; 77(6):501
968. Wall B, Walch G. Reverse shoulder arthroplasty for the treatment of proximal humeral fractures. Hand Clin. 2007; 23(4):425
969. Wallace MJ, Bledsoe G, Moed BR, Israel HA, Kaar SG. Relationship of cortical thickness of the proximal humerus and pullout strength of a locked plate and screw construct. J Orthop Trauma. 2012; 26(4):222
970. Walz H, Siebler G, Kuner EH. Possibilities of minimal osteosynthesis in multiple segment fractures of the proximal humerus. Z Unfallchir Versicherungsmed Berufskr. 1988; 81(2):133
971. Walz M, Kolbow B, Esmer E. Is functional outcome of operatively treated proximal humeral fractures in elderly patients influenced by patient's capability? Aktuelle Traumatol. 2007; 37(1):51
972. Wan Y, Xu L, Lu X, Zhang Z, Ge J, Chen G, Tan M. Effectiveness analysis of artificial humeral head replacement for 18 cases of complicated fractures of the proximal humerus. Chung-Kuo Hsiu Fu Chung Chien Wai Ko Tsa Chih. 2012; 26(2):205
973. Wang L, Zhuang CY, Zhang WB, Yang QM. Analysis of the results of hemiarthroplasty in the treatment of proximal humeral fractures. Chung-Hua Wai Ko Tsa Chih. 2007; 45(20):1389
974. Wanner GA, Romero J, Hersche O, Smekal A, Ertel W. Dislocated proximal humerus fracture--results after stabilization with a double plate. Langenbecks Archiv fur Chirurgie - Supplement – Kongressband. 1998; 115:1211
975. Wanner GA, Wanner-Schmid E, Romero J, Hersche O, von Smekal A, Trentz O, Ertel W. Internal fixation of displaced proximal humeral fractures with two one-third tubular plates. J Trauma-Injury Infect Crit Care. 2003; 54(3):536
976. Warnecke J, Jansen T, Oestern HJ. Surgical Treatment of proximal humerus fractures. Deutsche Gesellschaft fur Chirurgie. 1999; Suppl Kongressband II:1021
977. Warrender WJ, Oppenheimer S, Abboud JA. Nerve monitoring during proximal humeral fracture fixation: what have we learned?. Clin Orthop Relat Res. 2011; 469(9):2631
978. Watford KE, Jazrawi LM, Eglseder JR. Percutaneous fixation of unstable proximal humeral fractures with cannulated screws. Orthopedics. 2009; 32(3):166
979. Webb M, Funk L. An anterosuperior approach for proximal humeral fractures. Tech Shoulder Elbow Surg. 2006; 7(2):77
980. Weber E, Matter P. Surgical treatment of proximal humerus fractures--international multicenter study. Swiss Surgery. 1998; 4(2):95
981. Weber J, Westphal J, Schoen M. Proximal humeral fractures: Discrepancies between a predetermined clinical pathway and the actual clinical decision. Injury (Conf). 2012; 43:S12
982. Wei W, Zhuang Y, Zhang K, Lu DG. Minimally invasive percutaneous plate osteosynthesis for treatment of proximal humerus fractures with PHILOS plate. Nan Fang Yi Ke Da Xue Xue Bao. 2010; 30(11):2553
983. Weigand H, Muller HA, Gutjahr G, Ritter G. Classification of fractures of the proximal end of the humerus following prognostic and therapeutic aspects. Unfallchirurgie. 1984; 10(5):221
984. Weinstein DM, Bratton DR, Ciccone WJ, Elias JJ. Locking plates improve torsional resistance in the stabilization of three-part proximal humeral fractures. J Shoulder Elbow Surg. 2006; 15(2):239
985. Weise K, Meeder PJ, Wentzensen A. Indications and technique of osteosynthesis in dislocated fractures of the humeral head in adults. Langenbecks Arch Chir. 1980; 351(2):91
986. Weller S. Proximal humerus fracture. Z Orthop Unfallchir. 2008; 146(3):409
987. Werner A, Bohm D, Ilg A, Gohlke F. Intramedullary wire fixation of proximal humeral fractures - Modified Kapandji technique. Unfallchirurg. 2002; 105(4):332
988. Weseley MS, Barenfeld PA, Eisenstein AL. Rush pin intramedullary fixation for fractures of the proximal humerus. J Trauma-Injury Infect Crit Care. 1977; 17(1):29
989. Wheeler DL, Colville MR. Biomechanical comparison of intramedullary and percutaneous pin fixation for proximal humeral fracture fixation. J Orthop Trauma. 1997; 11(5):363
990. Widmer BJ, Bassora R, Warrender WJ, Abboud JA. Thromboembolic events are uncommon after open treatment of proximal humerus fractures using aspirin and compression devices. Clin Orthop Relat Res. 2011; 469(12):3332
991. Wiedemann E, Brunner U, Hauptmann S, Mutschler W. Hemiarthroplasty for humeral head fractures. Oper Orthop Traumatol. 2004; 16(1):1
992. Wiedemann E, Schweiberer L. Closed treatment of fractures of the humeral head. Indications, technique, limits. Orthopade. 1992; 21(2):106
993. Wijgman AJ, Roolker W, Patt TW, Raaymakers EL, Marti RK. Open reduction and internal fixation of three and four-part fractures of the proximal part of the humerus. J Bone Joint Surg Am. 2002; 84-A(11):1919
994. Wild J, Demers A, French R, Shipps M, Bergin P, Musapatika D, Jelen B. Functional Outcomes for Surgically Treated 3- and 4-part Proximal Humerus Fractures. Orthopedics. 2011; 34(10):768
995. Willems WJ, Lim TE. Neer arthroplasty for humeral fracture. Acta Orthop Scand. 1985; 56(5):394
996. Williams GR, Copley LA, Iannotti JP, Lisser SP. The influence of intramedullary fixation on figure-of-eight wiring for surgical neck fractures of the proximal humerus: a biomechanical comparison. J Bone Joint Surg. 1997; 6(5):423
997. Williams GR, Wong KL. Two-part and three-part fractures: open reduction and internal fixation versus closed reduction and percutaneous pinning. Orthop Clin North Am. 2000; 31(1):1
998. Wilson J, Bonner TJ, Head M, Fordham J, Brealey S, Rangan A. Variation in bone mineral density by anatomical site in patients with proximal humeral fractures. J Bone Joint Surg Br. 2009; 91B(6):772
999. Wirbel RJ, Knorr V, Mutschler W. Minimal invasive therapy in dislocated proximal humerus fractures. Influence of post-operative immobilisation on the function outcome Hefte zur der Unfallchirurg. 1997; 268:678
1000. Worsdorfer O. Classification of proximal humeral fractures. Hefte Unfallheilkd. 1982; 160:117
1001. Worsdorfer O, Magerl F. Surgical treatment of proximal humeral fractures. Hefte Unfallheilkd. 1982; 160:136
1002. Wretenberg P, Ekelund A. Acute hemiarthroplasty after proximal humerus fracture in old patients. A retrospective evaluation of 18 patients followed for 2-7 years. Acta Orthop Scand. 1997; 68(2):121
1003. Wu CH, Ma CH, Yeh JJH, Yen CY, Yu SW, Tu YK. Locked plating for proximal humeral fractures: Differences between the deltopectoral and deltoid-splitting approaches. J Trauma-Injury Infect Crit Care. 2011; 71(5):1364
1004. Wu G. The comparison of therapeutic effects between two operative methods in treating proximal humeral fractures. Modern Journal of Integrated Traditional Chinese and Western Medicine. 2008; 17(6):862,871
1005. Wu H, Cha ZG, Lin HS, Hou HG, Feng YH, Li JR. A comparative study between humeral head prosthesis replacement and internal fixation for treatment of comminuted proximal humeral fractures. Nan Fang Yi Ke Da Xue Xue Bao. 2010; 30(3):560
1006. Wu X, Lou LM, Chen ZR, Zhang GJ. Reconstruction and balance of soft tissue in hemi-shoulder replacement for patients with four-part fracture of the proximal humerus. Chung-Hua Wai Ko Tsa Chih. 2008; 46(19):1490
1007. Wychowanski M, Obrebski M, Rapala K, Wit A, Gajewski J, Marczak K. Strength of proximal humeral fraction fixation employing implants of various types - A study of porcine bones. Acta Bioeng Biomech. 2008; 10(3):29
1008. M. Xiang M, Chen H, Tang HC, Xie J. Treatment of two-part proximal humeral fracture with closed or mini-open assisted reduction and percutaneous pinning. Zhongguo Gushang. 2008; 21(12):919
1009. Xu W, Chen QZ, Gao H, Ji GL. Application of artificial shoulder prosthesis in proximal humeral fracture. Journal of Clinical Rehabilitative Tissue Engineering Research . 2010; 14(17):3159
1010. Yamada M, Briot J, Pedrono A, Sans N, Mansat P, Mansat M, Swider P. Age- and gender-related distribution of bone tissue of osteoporotic humeral head using computed tomography. J Shoulder Elbow Surg. 2007; 16(5):596
1011. Yamano Y. Comminuted fractures of the proximal humerus treated with hook plate. Arch Orthop Trauma Surg. 1986; 105(6):359
1012. Yan D, Soon Y. Comparative study of T-plates and locking plates in the management of displaced proximal humeral fractures. Curr Orthop Pract. 2012; 23(4):351
1013. Yang H, Li Z, Zhou F, Wang D, Zhong B. A prospective clinical study of proximal humerus fractures treated with a locking proximal humerus plate. J Orthop Trauma. 2011; 25(1):11
1014. Yang KH. Helical plate fixation for treatment of comminuted fractures of the proximal and middle one-third of the humerus. Injury. 2005; 36(1):75
1015. Yang L, Li B, Pan XY, Li C, Huang JW, Wang ZW, Chen H, Zhao YM, Chi YL. Percutaneous reduction and fixation of osteoporotic fractures for the proximal humerus in a geriatric population. Chung-Hua Wai Ko Tsa Chih. 2006; 44(12):830
1016. Yang SH, Wang J, Xu WH, Li J, Liu GH, Yang C, Ye SN, Ye ZW, Liu Y. Shoulder hemiarthroplasty for the treatment of complex proximal humeral fractures. Chin J Traumatol. 2009; 12(1):14
1017. Yeap JS, Noor Zehan AR, Ezlan S, Borhan Tan A, Harwant S. Functional outcome of proximal humeral fractures. Med J Malaysia 2001; 56(Suppl C):13
1018. Yildiz C, Kurklu M, Ozkan H, Bilgic S, Sehirlioglu A, Yurttas Y, Baykal B, Komurcu M, Basbozkurt M. AO tension band technique application in proximal humerus fractures. Eklem Hast Cerrahisi. 2010; 21(2):62
1019. Young AA, Hughes JS. Locked Intramedullary Nailing for Treatment of Displaced Proximal Humerus Fractures. Orthop Clin North Am. 2008; 39(4):417
1020. Young SW, Segal BS, Turner PC, Poon PC. Comparison of functional outcomes of reverse shoulder arthroplasty versus hemiarthroplasty in the primary treatment of acute proximal humerus fracture. ANZ J Surg. 2010; 80(11):789
1021. Yu Z, Zheng L, Wang Y, Zhang Y, Zhang X, Ma B. Functional and Radiological Evaluations of Unstable Displaced Proximal Humeral Fractures Treated with Closed Reduction and Percutaneous Pinning Fixation. Eur Surg Res. 2010; 45(3-4):138
1022. Yuan FS, Zhang ZY, Hu S, Zhang J, An JL, Yang CX. Clinical internal fixation strategy for treatment of senile osteoporotic complicated fracture of proximal humerus. Journal of Jilin University Medicine Edition. 2010; 36(3):578
1023. Yuksel HY, Yimaz S, Aksahin E, Celebi L, Muratli HH, Bicimoglu A. The results of nonoperative treatment for three- and four-part fractures of the proximal humerus in low-demand patients. J Orthop Trauma. 2011; 25(10):588
1024. Zeitler H, Blank M, Haberle HJ, Tomczak R, Pfeifer T, Hehl G, Hollen I. Morphologic roentgen findings after surgical management of proximal humeral fractures and their clinical value. Rontgenpraxis. 1994; 47(5):139
1025. Zettl R, Mueller T, Topp T, Lewan U, Krueger A, Kuehne C, Ruchholtz S. Monoaxial versus polyaxial locking systems: a biomechanical analysis of different locking systems for the fixation of proximal humeral fractures. Int Orthop. 2011; 35(8):1245
1026. Zhai WT, Zhong B, Jiang Y. Factors affecting the outcome of humeral head replacement. Chinese Journal of Clinical Rehabilitation. 2002; 6(22):3388
1027. Zhang H, Ni W, Gao S, Liang X, Zhou A. Long PHILOS locking compression plate for treatment of proximal humerus and humeral shaft fractures. Chung-Kuo Hsiu Fu Chung Chien Wai Ko Tsa Chih. 2009; 23(4):419
1028. Zhang JH, Di ZL, He ZY, Feng JX, Xu RM. Comparison of humeral head replacement and internal fixation for the treatment of 3 parts and 4 parts fractures of proximal humerus in the elderly. Zhongguo Gushang. 2010; 23(6):435
1029. Zhang J, Ebraheim N, Lause GE. Surgical treatment of proximal humeral fracture with external fixator. J Shoulder Elbow Surg. 2012; 21(7):882
1030. Zhang L, Zheng J, Wang W, Lin G, Huang Y, Zheng J, Prince GAE, Yang G. The clinical benefit of medial support screws in locking plating of proximal humerus fractures: a prospective randomized study. Int Orthop. 2011; 35(11):1655
1031. Zhang ZJ, Niu SL, Chang ZX, Ning FY. Study of the treatment of comminuted fractures of proximal humerus with open reduction and internal fixation. Zhongguo Gushang. 2009; 22(11):824
1032. Zhao JP, Hu WK, Zhang QL, Lin J, Zhou Q, He JJ, Zhuge TY. Application of PHILOS plate through mini-open deltoid-splitting approach for the treatment of proximal humeral fractures. Zhongguo Gushang. 2012; 25(2):155
1033. Zhao Y, Jin A, Liu JG, Yu TC. Influence of age and gender on the healing of three- and four-part proximal humerus fractures following cloverleaf plate fixation. Journal of Clinical Rehabilitative Tissue Engineering Research. 2008; 12(30):5904
1034. Zhou GX, Hou ZQ, Tan PX, Ye GH, Yao HS, Huang ZS. Surgical anatomy of extended anterolateral acromial approach for internal fixation of proximal humeral fracturesm. Journal of Clinical Rehabilitative Tissue Engineering Research. 2011; 15(35):6461
1035. Zhou R. Locking steel plate therapy for complex fracture of the proximal humarus: Technique operation in 23 cases. Journal of Clinical Rehabilitative Tissue Engineering Research. 2007; 11(41):8372
1036. Zhou ZB, Gao YS, Tang MJ, Sun YQ, Zhang CQ. Minimally invasive percutaneous osteosynthesis for proximal humeral shaft fractures with the PHILOS through the deltopectoral approach. Int Orthop. 2012; 36(11):2341
1037. Zhu N, Chen Y. Biomechanical analysis of stability of internal fixator for proximal humeral fractures. Chung-Kuo Hsiu Fu Chung Chien Wai Ko Tsa Chih. 2010; 24(12):1459
1038. Zhu Y, Lu Y, Shen J, Zhang J, Jiang C. Locking intramedullary nails and locking plates in the treatment of two-part proximal humeral surgical neck fractures: a prospective randomized trial with a minimum of three years of follow-up. J Bone Joint Surg Am. 2011; 93(2):159
1039. Zhu YM, Jiang CY, Lu Y, Wang MY. Fixation of proximal humeral fracture with proximal humeral locking intramedullary nail. Chung-Hua Wai Ko Tsa Chih. 2007; 45(20):1385
1040. Zhu Y, Lu Y, Wang M, Jiang C. Treatment of proximal humeral fracture with a proximal humeral nail. J Shoulder Elbow Surg. 2010; 19(2):297
1041. Zifko B, Poigenfurst J. Treatment of unstable fractures of the proximal end of the humerus using elastic curved intramedullary wires. Unfallchirurgie. 1987; 13(2):72
1042. Zifko B, Poigenfurst J, Pezzei C. Intramedullary nailing of unstable proximal humeral fractures. Orthopade. 1992; 21(2):115
1043. Zifko B, Poigenfurst J, Pezzei C, Stockley I. Flexible intramedullary pins in the treatment of unstable proximal humeral fractures. Injury. 1991; 22(1):60
1044. Zimmermann J. Treatment physical training after upper extremity fractures with direction or proximal humerus. Rehabilitacia. 2003; 40(3):164
1045. Zingg U, Brunnschweiler D, Keller H, Metzger U. Percutaneous minimal osteosynthesis of fractures of the proximal humerus in elderly patients. Swiss Surgery. 2002; 8(1):11
1046. Zuckerman JD, Cuomo F, Koval KJ. Proximal humeral replacement for complex fractures: indications and surgical technique. Instr Course Lect. 1997; 46:7
1047. Zygas P. Incidence of osteonecrosis of the femoral head following fracture of the proximal humerus. Acta Orthop Belg. 1999; 65(SUPPL. 1):99
1048. Zyto K. Non-operative treatment of comminuted fractures of the proximal humerus in elderly patients. Injury. 1998; 29(5):349
1049. Zyto K, Ahrengart L, Sperber A, Tornkvist H. Treatment of displaced proximal humeral fractures in elderly patients. J Bone Joint Surg. 1997; 79(3):412
1050. Zyto K, Kronberg M, Brostrom LA. Shoulder function after displaced fractures of the proximal humerus. J Bone Joint Surg. 1995; 4(5):331
1051. Zyto K, Wallace WA, Frostick SP, Preston BJ. Outcome after hemiarthroplasty for three- and four-part fractures of the proximal humerus. J Bone Joint Surg. 1998; 7(2):85
